# Alteration of microbiota antibody‐mediated immune selection contributes to dysbiosis in inflammatory bowel diseases

**DOI:** 10.15252/emmm.202115386

**Published:** 2022-07-04

**Authors:** Eva Michaud, Louis Waeckel, Rémi Gayet, Roman Goguyer‐Deschaumes, Blandine Chanut, Fabienne Jospin, Katell Bathany, Magali Monnoye, Coraline Genet, Amelie Prier, Caroline Tokarski, Philippe Gérard, Xavier Roblin, Nicolas Rochereau, Stéphane Paul

**Affiliations:** ^1^ CIRI – Centre International de Recherche en Infectiologie, Team GIMAP, Univ Lyon, Université Claude Bernard Lyon 1, Inserm, U1111, CNRS, UMR530, CIC 1408 Vaccinology Saint‐Etienne France; ^2^ Chimie et Biologie des Membranes et des Nano‐objets (UMR 5248) Université de Bordeaux, CNRS, Bordeaux INP Pessac France; ^3^ Micalis Institute, INRAE, AgroParisTech Université Paris‐Saclay Jouy‐en‐Josas France; ^4^ Inserm UMR 1098 Right Université Bourgogne Franche‐Comté Besançon France

**Keywords:** SIgA, microbiota, glycosylation, IBD, immunity, Digestive System, Immunology, Microbiology, Virology & Host Pathogen Interaction

## Abstract

Human secretory immunoglobulins (SIg) A1 and SIgA2 guide mucosal responses toward tolerance or inflammation, notably through reverse‐transcytosis, the apical‐to‐basal transport of IgA2 immune complexes via M cells of gut Peyer's patches. As such, the maintenance of a diverse gut microbiota requires broad affinity IgA and glycan–glycan interaction. Here, we asked whether IgA1 and IgA2‐microbiota interactions might be involved in dysbiosis induction during inflammatory bowel diseases. Using stool HPLC‐purified IgA, we show that reverse‐transcytosis is abrogated in ulcerative colitis (UC) while it is extended to IgA1 in Crohn's disease (CD). 16S RNA sequencing of IgA‐bound microbiota in CD and UC showed distinct IgA1‐ and IgA2‐associated microbiota; the IgA1^+^ fraction of CD microbiota was notably enriched in beneficial commensals. These features were associated with increased IgA anti‐glycan reactivity in CD and an opposite loss of reactivity in UC. Our results highlight previously unknown pathogenic properties of IgA in IBD that could support dysbiosis.

## Graphical abstract

IBD (both CD and UC) is characterized by dysbiosis and altered immune pathways that lead to and sustain prolonged inflammation in the gut. As IgA are the main drivers of commensal selection in the healthy gut, this study aimed at assessing subclass‐related structure and functions of IgA in both CD and UC.

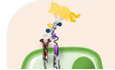

## Introduction

Mutualism between host immunity and microbial communities at the gut mucosal surface is critical for the education of the immune system and the processing of digested foods. Intestinal epithelial cells allow the transcytosis of secretory IgA (SIgA) to promote the maintenance of beneficial taxa. SIg are characterized by J‐chain‐mediated multimerization and secretory component (SC) binding, enabling their efficient trafficking to and from the mucosal surface (Brandtzaeg, 1981). SIgA promotes the establishment of bacterial networks (Mathias *et al*, 2014; Rios *et al*, 2016) through both glycan–glycan interactions (Perrier *et al*, 2006; Rollenske *et al*, 2018) and Fab binding (Sterlin *et al*, 2020), a process known as antibody‐mediated immune selection (AMIS; Donaldson *et al*, 2018; Hoces *et al*, 2020; Moor *et al*, 2017; Nagashima *et al*, 2017; Pabst, 2012; Rollenske *et al*, 2018). Both low‐affinity natural SIgA and high‐affinity SIgA contribute to AMIS and are thought to, respectively, promote commensal selection (T‐independent signals), and selectively identify fast‐replicating bacteria as pathogenic (T‐dependent signals; Bansept *et al*, 2019; Hoces *et al*, 2020; Moor *et al*, 2017; Neumann *et al*, 2019). Heavy glycosylation of SIgA favors mucus entrapment of SIgA‐bound bacteria and symbiosis within the microbiota (Donaldson *et al*, 2018; Rollenske *et al*, 2018; Steffen *et al*, 2020). Human IgA1 and IgA2 have distinct patterns of glycosylation as IgA1 is mostly O‐glycosylated (Novak *et al*, 2012; Ohyama *et al*, 2020), and IgA2 is mostly N‐glycosylated (Steffen *et al*, 2020). On their shared two sites of N‐glycosylation, sialylation of IgA1 appears to dampen its natural pro‐inflammatory effects (Steffen *et al*, 2020). Moreover, anti‐commensal IgA1 signaling tends to elicit tolerogenic signals such as T_reg_ induction and IL‐10 secretion (Sterlin *et al*, 2020). Sialylation and N‐glycosylation of SIgA2 enables reverse‐transcytosis (RT), a process by which antigen‐bound SIgA2 travels from the lumen toward the subepithelial dome of Peyer Patches by binding Dectin‐1 receptors on M‐cells (Rochereau *et al*, 2013).

Strong evidence supports that dysbiosis within the intestinal microbiota—that is, the alteration of resident commensal ecosystems in favor of non‐beneficial communities—is responsible for inflammatory bowel diseases (IBD) defects (Joossens *et al*, 2011; Ni *et al*, 2017). In both Crohn's disease (CD) and ulcerative colitis (UC), the *Firmicutes/Bacteroidetes ratio* decreases (Mariat *et al*, 2009; Fuentes *et al*, 2017; Ni *et al*, 2017; Pascal *et al*, 2017). In CD, this is mostly attributed to reduced diversity amongst *Firmicutes*, the resurgence of atypical species such as segmented filamentous bacteria (SFB) and the colonization of the mucosa by oropharyngeal species (Rengarajan *et al*, 2019; Yamada *et al*, 2019). As such, CD has been strongly and extensively associated with the loss of short‐chain fatty acids (SCFA) producers (Liu *et al*, 2016; Quévrain *et al*, 2016; Takahashi *et al*, 2016; Yamada *et al*, 2019; Yilmaz *et al*, 2019). Dysbiosis in UC is associated with the reduction in both *Firmicutes* and *Bacteroidetes* abundance, despite a higher diversity than in CD (Yilmaz *et al*, 2019), and decreased SCFA producers abundance (Machiels *et al*, 2014; Mirsepasi‐Lauridsen *et al*, 2018). A noticeably lower representation of the mucolytic *Akkermansia* species was also evidenced in UC patients compared with healthy controls (den Besten *et al*, 2013; Moor *et al*, 2017; Lopez‐Siles *et al*, 2018). In healthy humans, despite IgA1 binding to bacteria usually being concomitant to that of IgA2, IgA1 displays a preference toward *Actinobacteria* selection while IgA2 identification favors *Bacteroidetes* selection (Sterlin *et al*, 2020), suggesting dual contributions to microbiome diversity. Coincidentally, IBD patients' stools show increased levels of IgA‐bound bacteria (Rengarajan *et al*, 2019), either commensal or opportunistic, and shift from dimeric IgA secretion toward monomeric IgA secretion (MacDermott *et al*, 1986).

It is very likely that SIgA repertoires are affected over the course of IBD, resulting in aberrant responses to normally tolerated strains. This would leave room for opportunistic species to colonize niches they do not normally have access to. Current therapeutic strategies rely on anti‐inflammatory and immunosuppressive biological agents. Noticeably in CD, anti‐TNF failure is associated with low microbiota diversity and loss of short‐chain fatty acids (SCFA)‐producing taxa (Yilmaz *et al*, 2019). Other agents such as antibiotics, thiopurines, and corticosteroids enhance the effect of anti‐TNF therapies (Bernstein, 2015; Feuerstein & Cheifetz, 2017; Townsend *et al*, 2019), but most available drugs are associated with long lists of adverse effects that have yet to be overcome. Fecal microbiota transplantation (FMT) could limit the use of such aggressive therapies but has been met with heterogenous outcome and reproducibility rates in IBD studies; it mostly results in short‐term improvements in IBD patients, and sometimes triggers IBD flares (Shi *et al*, 2016; Allegretti *et al*, 2017; Bak *et al*, 2017; Benech & Sokol, 2020). We believe that the role of IgA and its selection properties in IBD might have been overlooked and could, at least in part, explain this mosaicism.

Here, the functional abilities of human secretory IgA1 and IgA2 in terms of receptor recognition, glycosylation pattern, trafficking, and microbial selection in a small cohort of CD and UC patients has been explored. Fecal SIgA in IBD are abnormally reactive to self and commensal glycans, and we describe novel specific IgA1‐associated and IgA2‐associated microbial profiles in CD and UC, respectively. Moreover, we show that while CD is concurrent with enriched microbial selection by SIgA1, UC occurrence is on the contrary associated with a striking loss in both IgA functionality and reactivity.

## Results

### Patients with IBD have altered SIgA1 and SIgA2 levels

**Figure 1:**
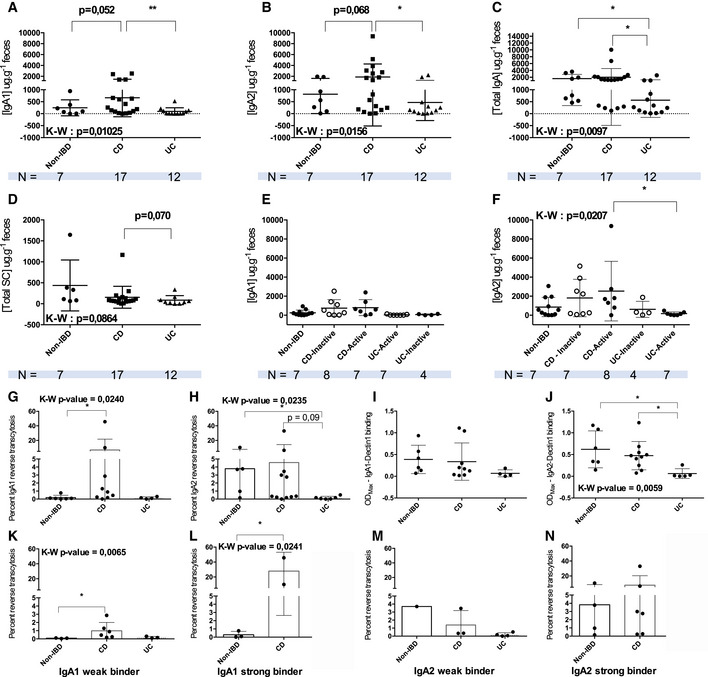
Purified fecal IgA1 and IgA2 from IBD patients have altered functionality related to Dectin‐1 binding A–DELISA assay of fecal IgA1 (A), IgA2 (B), total IgA (C), and secretory component (D) levels from non‐IBD (*n* = 7), CD (*n* = 18), and UC (*n* = 12) patients' stool.E, FData were further separated according to disease activity for IgA1 (E) and IgA2 (F).G, H
*In vitro* assay of purified IgA1 (G) and IgA2 (H) reverse‐transcytosis abilities on an inverted model of FAE from Caco2 and Raji cells co‐culture.I, J(I) ELISA assay of IgA1‐Dectin‐1 binding, at a rate of one receptor per 10 IgA; (J) ELISA assay of IgA2‐Dectin‐1 binding, at a rate of one receptor per 10 IgA.K–NPercent of IgA1 (K, L) and IgA2 (M, N) reverse‐transcytosis for weak (K, M) and strong (L, N) Dectin‐1 binding. Data information:  Data were analyzed using Kruskall–Wallis multiple comparisons with Dunn's correction, when possible, or a Mann–Whitney test. *P*‐values are as follows: (A) **P = 0.0098; (B) *P = 0.0406; (C) Non‐IBD vs. UC *P = 0.0479, CD vs. UC *P = 0.0166; (F) *P = 0.0167; (G) *P = 0.0441; (H) *P = 0.423; (J) Non‐IBD vs. UC *P = 0.0199, CD vs. UC *P = 0.0243; (K) *P = 0.0221; (L) *P = 0.0486. (G–N) For some patients, antibody purification did not yield a high enough concentration, so samples had to be excluded. *N* are thus as follows: IgA1: Non‐IBD: *n* = 7; CD: *n* = 8; UC: *n* = 4; IgA2: Non‐IBD: *n* = 7; CD: *n* = 8; UC: *n* = 5. All patient samples (biological replicates) have been tested in technical duplicates meaning *n* × 2.

To account for the homogeneity of the IBD spectrum and to avoid analysis bias as much as possible, we included patients based on their endoscopic scores at sample collection and treatment history but regardless of phenotype. To explore the biological significance of both human IgA subclasses during chronic mucosal inflammation, we first measured total IgA, IgA1, and IgA2 concentrations in stool homogenates in Non‐IBD, CD, and UC individuals. In CD, IgA1 and IgA2 levels only tended to increase in CD relative to non‐IBD (Fig 1A and B), while decreasing significantly in UC compared with CD patients (Fig 1C). UC samples also trended toward lower total SC levels (Fig 1D). Disease activity at the time of sample collection did not affect IgA1 and IgA2 concentrations in CD (Fig 1E and F), while a significant decrease in active UC IgA2 stool concentration was evidenced compared with both active CD disease (Fig 1F). Interestingly, in all assays, the distribution of data points hinted at subgroups within IBD groups and particularly in the CD cohort. These clusters of patients with high versus low IgA, IgA1, and IgA2 levels were not correlated with age, disease duration, phenotype, or sex (Appendix Table S2).

### SIgA retro‐transport is distinctly modified in CD and UC

As there was no evident change in IgA1 and IgA2 concentrations in patients' stool, we next asked whether IgA functionality remained intact relative to the non‐IBD cohort. Using a previously described *in vitro* model of FAE (Rochereau *et al*, 2013), the reverse‐transcytosis (RT) potential for stool‐purified IgA1 and IgA2 was evaluated. Of note, stool IgA have undergone digestion when they are purified and were probably damaged in the process. While this is unavoidable, it is probably why the efficiency of the antibody purification was lower than that of serum IgA.

In non‐IBD patients, we confirm that only IgA2 could perform RT (Fig 1H), as IgA1 percent RT was negligible (Fig 1G). In IBD groups, purified CD IgA1 but not UC IgA1 was also able to undergo RT (Fig 1G). Oppositely, no RT could be evidenced when using UC IgA2 (Fig 1H).

As IgA2 RT activity is associated with Dectin‐1 binding, we performed an ELISA‐based Dectin‐1 binding assay using IgAs from the same patients. Regardless of the group, IgA1 binding to Dectin‐1 was generally low and without significant differences between our non‐IBD, CD, and UC groups (Fig 1I). IgA1 percent RT was, however, correlated with Dectin‐1 binding, contrary to Non‐IBD or UC IgA1 (Appendix Table S2). This was surprising, given IgA1 from CD appeared to have gained RT functions (Fig 1G). We thus explored whether this discrepancy could originate from changes in binding affinity to the RT co‐receptor Siglec‐5. A similar ELISA‐based assay demonstrated there was no significant difference in binding affinities for Siglec‐5 in CD IgA1 (Appendix Fig S2A).

For IgA2, we observed a significantly lower affinity for Dectin‐1 compared with both non‐IBD and CD individuals in the UC group (Fig 1J), hinting that the lower levels of RT observed in UC could be attributed to defective Dectin‐1 recognition.

As we used IgAs from the same patients for both Dectin‐1 binding and RT experiments, strong and weak Dectin‐1 binders according to their level of RT were compared. Strong IgA2 binding of non‐IBD samples was associated with higher RT percentages (Appendix Fig S2B). For weak binding, our cohort only included one weak IgA2 binder in the non‐IBD group (Appendix Fig S2B). In CD, strong IgA1 binding to Dectin‐1 was associated with higher levels of RT than those of strong IgA2 binding (Appendix Fig S2C). Weak Dectin‐1 IgA1 binders in CD still retained a significantly higher RT levels than non‐IBD weak binding IgA1 (Fig 1K). This suggests that the regulation of IgA1 exclusion at the M‐cell apical membrane for the induction of RT might be dysregulated in those CD patients. Consistently, high levels of IgA1 RT were also correlated with strong Dectin‐1 binding in CD relative to non‐IBD IgA1 (Fig 1L and Appendix Table S3). Weak and strong IgA1 to Dectin1 binding affinity was confirmed by biolayer interferometry and representative association‐dissociation curves are depicted in Appendix Fig S2E. Oppositely, IgA2 binding strength was not discriminative for RT levels between non‐IBD nor CD IgA2 (Appendix Table S3) as non‐IBD and CD IgA2 had high levels of RT regardless of Dectin‐1 affinity (Fig 1M and N). There were no strong IgA1 or IgA2 binders in UC (Fig 1K–N and Appendix Fig S3D), which further suggest defective recognition for IgAs in UC. Overall, these results hint at a possible aberrant functions of IgA1 in CD and the loss of typical IgA2 functions in UC IgA2.

### Glycosylation profiles from stool‐purified IgA differ between CD and UC patients

Because interaction with Dectin‐1 and Siglec‐5 is highly dependent on IgA glycosylation recognition (Rochereau *et al*, 2013), we hypothesized that changes in retransport ability of both CD and UC patients' IgAs could originate from structural alterations. Particularly, we expected differential glycosylations profiles that would enhance or dampen receptor binding and could potentiate uptake by M cells, which we evaluated by mass spectrometry. IgA1 (UniProtKB P01876) has two N‐linked glycans located on N144 and N340 residues (enumerated starting from the N‐terminal residue of the heavy constant chain), and a hinge region including five serine and four threonine residues that are nine potential O‐glycosylation sites. The constant region of the heavy chain of IgA2 (UniProtKB P01877) is glycosylated on four asparagine residues: N92, N131, N205, and N327. Appendix Table S4 shows the N/O‐glycosylations carrying peptides of IgA1 and IgA2 consistently with our experimental design based on trypsin digestion.

#### N‐glycan microheterogeneity

**Figure 2:**
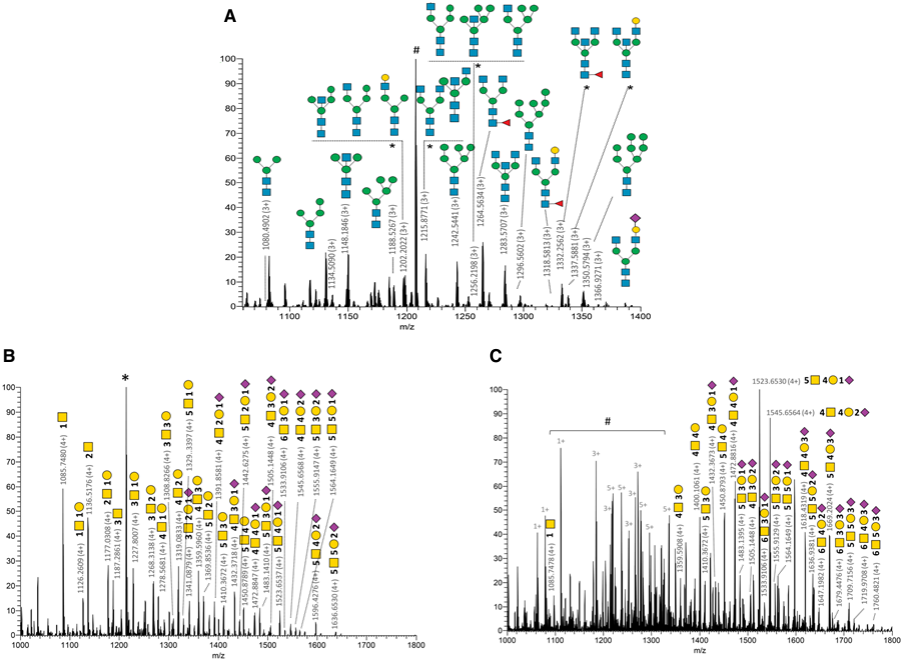
CD IgA1 N and O‐glycosylations do not recapitulate conventional IgA glycosylation patterns AFull MS spectrum of glycopeptides released from CD IgA1 trypsin digest. Main N340‐glycoforms of the IgA1 glycopeptide [332–353] (LAGKPTHVNVSVVMAEVDGTCY) are annotated using CFG nomenclature. Appendix Table S4 summarizes a list of N‐glycopeptides identified. # is the peptide [43–76] of immunoglobulin kappa constant chain (UniProtKB P01834). *Isobaric structures not differentiated by MS/MS experiments (not exhaustive N‐glycans illustrations). (HYTNPSQDVTVPCPVPSTPPTPSPSTPPTPSPSCCHPR).BO‐glycoforms identified for CD.CO‐glycoforms identified for UC. Data information: *corresponds to IgA1 peptide [264–273] (WLQGSQELPR). ^#^shows contamination by other multiply charge species covering the glycoforms signals. Details related to O‐glycoforms are given in Appendix Table S7. CD: *n* = 3; UC: *n* = 1 (biological replicates). All patient samples have been tested in technical duplicates.

We characterized N‐glycopeptides spanning the two N‐glycosylation sites of IgA1 and all the sites of IgA2 except the N327 C‐terminus site (Glycan heterogeneity listed in Appendix Table S5). The N‐glycosylation identified on N340 in CD IgA1 (73.5–74.5 and 80.0–81.0 min for sialo‐glycopeptides) is mainly composed of high mannose structures and bi‐antennary complex type N‐glycans mostly truncated complex structures with terminal GlcNAc (Fig 2A). Several single fucosylated glycans were identified as core fucosylated (Fig 2A). Surprisingly, these structures had a very low sialylation rate with only one N‐glycan carrying a terminal Neu5Ac sialylation. This is of particular interest given sialylation, especially on IgA1 (but not limited to) appears to have an immunosuppressive effect (Colucci *et al*, 2015; Steffen *et al*, 2020). Several of these forms were previously described for SIgA (Royle *et al*, 2003; Huang *et al*, 2015a) and serum IgA1 (Chandler *et al*, 2019) such as Man5 to Man8 structures and G0 (IgG glycans naming system) to G2 structures including bisecting GlcNAc (e.g., G0B, G0FB, and G1B). MS/MS spectra have discriminated several isobaric structures such as Man3B instead of G0‐N (*m/z* 1,148.1846 on Fig 2A) using glycopeptide fragment ions with consecutive loss of two hexose moieties. The presence of hypothetical hybrid and tri‐antennary structures was not confirmed by our MS/MS dataset. Despite good peptide sequence coverage, lower S/N spectra were obtained for UC IgA1 N‐glycan that prevent a confident structural identification on this sample subpopulation.

The identified N‐linked glycans of IgA2 heavy chain are localized as expected on N92, N131, and N205 residues. Similar structures were identified for CD and UC samples: G0F and G0FB‐like structures were identified on N92 site of the [89–102] peptide (HYTNSSQDVTVPCR) and high mannose and bi‐antennary complex type N‐glycans were identified on N131 and N205 (Appendix Table S6).

Whereas high mannose N‐glycans were similarly observed in IgA1 and IgA2 region of sequence identity, bi‐antennary and bisecting glycans with higher complexity were observed for IgA1 up to G2 structures (G0/G0B for IgA2).

#### O‐glycan microheterogeneity

The IgA1 hinge region contains up to nine potential O‐glycosylation sites resulting in a highly heterogeneous patterns of O‐glycoforms as shown in previous studies related to SIgA (Royle *et al*, 2003;Huang *et al*, 2015a; Plomp *et al*, 2018) or serum IgA1 (Tarelli *et al*, 2004; Renfrow *et al*, 2007; Takahashi *et al*, 2010; Franc *et al*, 2013; Ohyama *et al*, 2020; Steffen *et al*, 2020). Several of these nine sites were shown to be O‐glycosylated (see UniProtKB P01876 and (Renfrow *et al*, 2007; Wada *et al*, 2010) for details). Our experimental design based on the study of the glycopeptide [89–126] resulted in the identification of almost 60 O‐glycosylated forms including up to six GalNAc‐clustered O‐glycans (Appendix Table S7), several of them were referenced earlier (Royle *et al*, 2003; Huang *et al*, 2015a). For example, the literature (Huang *et al*, 2015a; Plomp *et al*, 2018) references 4.8 and 4.6 O‐glycans per heavy chain of secretory IgA, respectively, from human colostrum and saliva collected from healthy donors. 32% of the O‐glycoforms contained 6 GalNAc and 85% were sialylated (on average 2.4 sialic acid per peptide) with up to 5 Neu5Ac (49).

In our dataset, approximately 30 and 25% of identified O‐glycoforms contained, respectively, 5 GalNAc and 4 GalNAc in both CD and UC. We detected on average 3.8 and 4.4 O‐glycans per heavy chain, respectively, in CD and UC patients. 21% of CD IgA1 O‐glycoforms contain less than 3 GalNAc versus 9% for UC and only 10% of identified O‐glycoforms from the CD patients contained 6 GalNAc versus 23% for UC. Despite the lower S/N of UC spectra compared with CD, slightly higher number of sialylated glycopeptides were identified in UC with up to 3 Neu5Ac versus a maximum of two identified in CD samples (Fig 2B and C). Around 48% of the CD glycoforms were sialylated (on average 0.6 sialic acid per peptide) versus 67% for UC (on average 1.3 sialic acid per peptide). The relative intensity of Neu5Ac‐based moieties compared with asialo compounds tends to be higher for UC than CD. Our results show a deficiency in O‐glycosylation in CD IgA1 compared with UC IgA1, as well as differences in the glycan composition, such as the absence of terminal sialic acid.

### Anti‐glycan‐reactivity of SIgA from IBD patients differs from that of non‐IBD

Glycan–glycan interactions are the basis of antibody–microbiota interaction at mucosal surfaces (Moor *et al*, 2017; Nakajima *et al*, 2018; Yamada *et al*, 2019). On top of IgA‐borne glycosylations, it was thus also paramount to investigate IgA anti‐glycan reactivity. As we evidenced discrepancies in CD and UC IgA ability to undergo RT, we wanted to get a fuller picture of IgA‐antigen relationships in IBD to infer on the potential quality of antigens delivered to the mucosa via RT in an IBD context. This is specifically relevant knowing these antigens will mostly be bacterial and self‐glycans that can intensely polarize immune responses. Stool‐purified IgA from the same patients used in prior experiments of this study were thus tested in glycan arrays. In principal components analysis (PCA) combining results for both total IgA, IgA1, and IgA2 anti‐glycan reactivity, UC samples clustered separately from CD and non‐IBD (Appendix Fig S3A–C). Combining IgA1 and IgA2 datasets for glycan reactivity also highlighted changes in CD IgA reactivity patterns that mostly affected sialylated glycans. Indeed, CD IgA bound significantly less Neu5Ac‐2,3‐Gal‐1,4‐Glc‐Sp1 relative to non‐IBD IgA, while two others, SGP and Neu5Ac‐2,3‐Gal‐1,4‐(Fuc‐1,3)‐Glc‐[3‐Sialyl‐3‐fucosyllactose/F‐SL], were significantly more targeted (Appendix Fig S3D and Table S8). Strikingly, a marked loss of recognition for more than 45 glycans by UC IgAs compared with both non‐IBD and CD samples was observed (Appendix Fig S3E).

**Figure 3:**
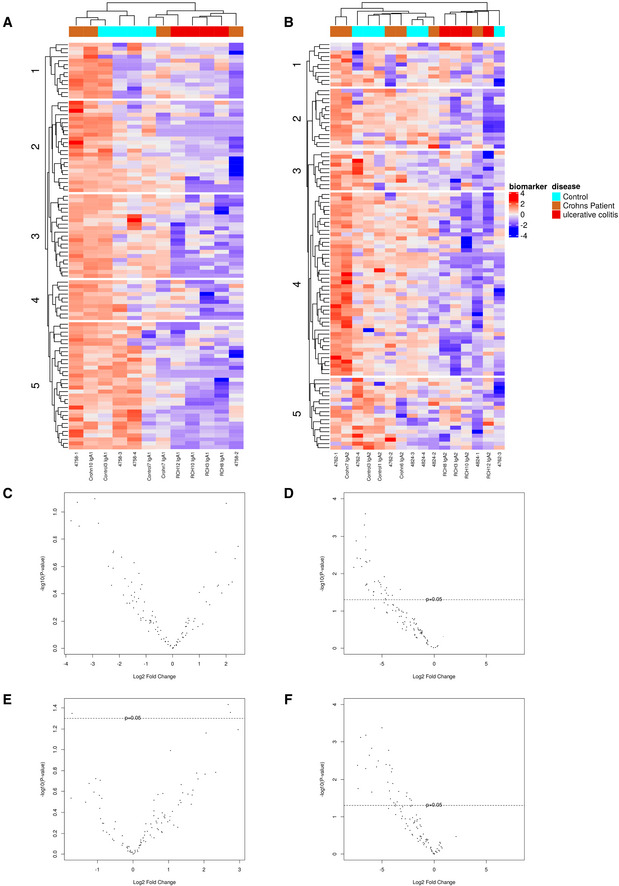
CD and UC IgA have distinct anti‐glycan reactivity profiles A, BHeatmaps displaying standardized log2‐transformed signal intensities per glycan for IgA1 (A) and IgA2 (B) in non‐IBD, CD and UC groups, plotted by hierarchical clustering of Euclidean distance.C–FVolcano plots of log2‐transformed fold changes for IgA1 anti‐glycan reactivity in CD (C) and UC (D) groups, and IgA2 anti‐glycan reactivity for CD (E) and UC (F) compared to non‐IBD IgA1 and IgA2. For CD, *P*‐values of differentially targeted glycan motifs are listed in Appendix Table S8 and those of UC in Appendix Tables S9 and S10. Significance threshold was placed at *P*‐value < 0.05 (indicated by the dotted line in C–F); For IgA1, Non‐IBD: *n* = 4; CD: *n* = 4; UC: *n* = 4. For IgA2, *n* = 6; CD: *n* = 6; UC: *n* = 6. IgA1 and IgA2 were taken matched for the same patient. All patient samples (biological replicates) have been tested in technical duplicates. A list of the glycans from each cluster is provided as Appendix Tables S11 and S12.

To get a general overview of anti‐glycan reactivity specifically associated with each IgA subclass, we first built heatmaps out of log2‐transformed relative fluorescence intensities per glycan (Fig 3A and B). Remarkably, data clustering recapitulated CD and UC groups for IgA1 (Fig 3A); however, CD and non‐IBD groups were not strictly separated and only appeared to differ for cluster 1 and cluster 2. These two clusters include mostly Gal‐branched glycans, antibiotics, and sialylated antigens (detailed list in Appendix Table S11), suggesting these structures might be interesting biomarkers for IBD diagnosis, providing validation on a larger number of subjects. Oppositely, UC IgA1 were clearly less bound to most glycans of this panel (Fig 3A). This suggests—as did the PCA—that UC IgA1 differs in terms of reactivity from its CD and non‐IBD counterparts. A similar observation can be made for IgA2 (Fig 3B and Appendix Table S12): UC IgA2 datasets clustered separately—and with lower fluorescence intensities—from their CD and non‐IBD counterparts, which were again quite closely clustered.

Importantly, there appeared to be non‐negligible intra‐group variability in both the CD and the UC groups that prompted us to look for statistically significant outliers. Statistical analysis of IgA1 anti‐glycan reactivity showed lower recognition of one disaccharide (4‐P‐GlcNac‐1,4‐Man‐Sp), one α‐Gal (Gal‐1,4‐Gal‐1,4‐Glc), and a higher affinity toward one sialylated oligosaccharide (Gal‐1,3‐(Neu5Ac‐2,6)‐GalNac, *P* < 0.1) by CD IgA1 relative to non‐IBD IgA1 (Fig 3C and Appendix Table S8). In contrast, UC IgA1 anti‐glycan reactivity was again significantly lessened for 34 glycans (Fig 3D).

As for IgA2, there was a significant increase in Neu5Ac‐2,3‐Gal‐1,4‐(Fuc‐1,3)‐Glc‐[3‐Sialyl‐3‐fucosyllactose/F‐SL] and maltotetraose recognition by CD IgA2 compared with non‐IBD IgA2 (Fig 3E and Appendix Table S8). Neu5Ac‐2,3‐Gal‐1,4‐Glc, was significantly less bound by CD IgA2 relative to non‐IBD IgA2 (Fig 3E and Appendix Table S8). As it was also less targeted by total CD IgAs, this suggests IgA2 was the main driving force behind the lower recognition of this glycan. For UC IgA2, we once more observed a loss in reactivity toward more that 40 glycans (Fig 3E and F).

Overall, these findings highlight potentially distinctive IgA subclass repertoires in CD and UC that will require a validation cohort to confirm our observations.

### IBD stool SIgA1 and SIgA2 coat similar proportions of the overall microbiota

**Figure 4:**
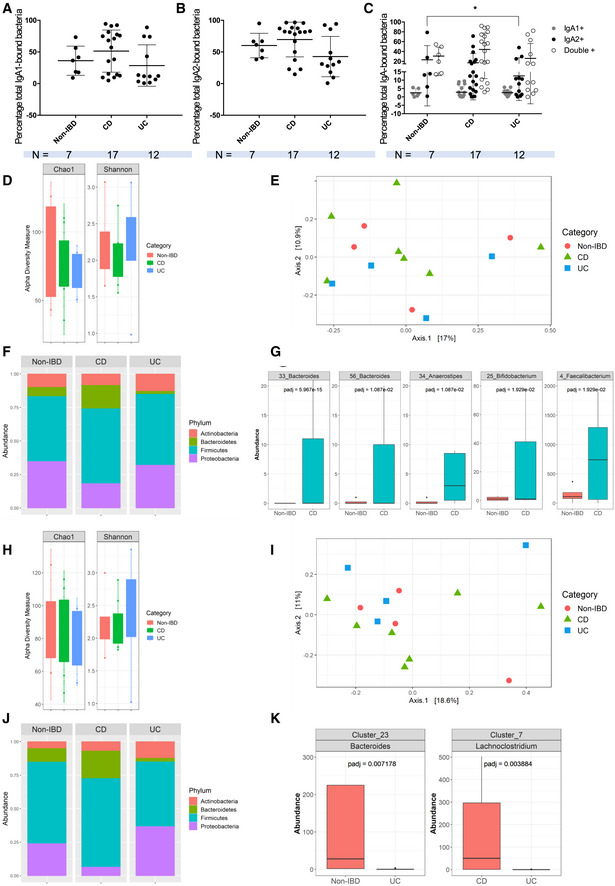
CD IgA1 have biased selection toward the microbiota A–CFlow cytometry analysis of total IgA1‐bound (A) and IgA2‐bound (B) fecal microbiota. (C) Flow cytometry analysis of stool IgA1^+^ IgA2^−^ bacteria (gray dots), IgA1^−^ IgA2^+^ bacteria (black dots) and IgA1^+^ IgA2^+^ bacteria (empty dots). (A–C) Data were analyzed with a Kruskall–Wallis multiple comparison test. In (C) *P = 0.0226. Non‐IBD: *n* = 7, CD: *n* = 18, and UC: *n* = 12.DShannon and Chao1 diversity indices of IgA1‐bound microbiota in non‐IBD, CD and UC.EPCA plot based on the Jaccard distance between samples.FPhylum‐level composition of IgA1‐bound microbiota in non‐IBD, CD and UC.GBoxplots of OTUs for which the abundance was significantly different between non‐IBD and CD.HShannon and Chao1 diversity indices of IgA2‐bound microbiota in non‐IBD, CD and UC.IPCA plot based on the Jaccard distance between samples.JPhylum‐level composition of IgA2‐bound microbiota in non‐IBD, CD and UC.KBoxplots of OTUs whose abundance was significantly different between CD and UC, and between non‐IBD and UC, respectively. OTUs present in less than 25% of samples or with read count lower than 50 were filtered out. Differential abundance was tested using negative binomial model implemented in DESeq2 and p‐values corrected with False Discovery Rate (FDR) procedure. Non‐IBD: *n* = 4; CD: *n* = 4; UC: *n* = 4 (two patients excluded for abundance in both IgA1 and IgA2 analyses). All patient samples have been tested in technical duplicates.

Given the distinct reactivity profiles of IgA1 and IgA2 in both our IBD groups, we supposed IgA‐mediated selection of the microbiota might be affected differently in both diseases and subclasses. We first evaluated the general level of stool microbiota bound to either IgA1, IgA2, or both in IBD by flow cytometry. There were no differences in the levels of total IgA1^+^‐or total IgA^+^‐bound stool microbiota (Fig 4A and B) between either group of IBD and non‐IBD individuals. Again, datasets in both IBD groups were grouped in two subclusters. Our first hypothesis was that disease status might affect bacteria binding levels, but subdivision of IBD groups into active versus inactive disease did not account for this clustering (Appendix Fig S4A–C). Hence, we next singled out single‐Ig‐positive and double‐positive bacteria (Fig 4C). While no differences between CD and non‐IBD groups were evidenced, IgA1^−^ IgA2^+^‐coated bacteria levels were lower in UC than in non‐IBD patients (Fig 4C) suggesting IgA2 coating of microbiota might be defective in UC patients. This analysis did not, however, get rid of these high and low levels of bound bacteria subgroups. Further correlations with clinical data showed that IgA1^+^ IgA2^−^ frequencies were strongly correlated to disease duration in CD (Appendix Fig S4E). No such association could be evidenced for the UC cohort, which again highlights IgA1‐related oddities in CD that have not been previously described. Of note, the known heterogeneity of IBD, which was voluntarily preserved in our IBD cohorts, could also be a consistent explanation for those subgroups.

### CD is characterized by IgA1‐driven microbiota selection defects

As no clear trend could be evidenced from our flow cytometry analysis, we performed more in‐depth analysis of 16S rDNA sequencing on magnetically separated IgA1‐ and IgA2‐bound fractions of the stool microbiota in non‐IBD, CD and UC subjects. Phylum composition of whole stool samples across groups is given in Appendix Fig S4F. For the IgA1‐bound fraction, ChaO1 and Shannon diversity indices did not differ between CD, UC, and non‐IBD groups, indicating similar bacterial richness (Fig 4D). Likewise, principal coordinate analysis (PCA) of the Jaccard distances did not separate CD, UC, and non‐IBD microbial profiles, indicating similar global community structure (Fig 4E). We also used Bray–Curtis index which came to the same conclusions. Nevertheless, at the phylum level, microbial composition in CD patients compared with the two other groups showed a higher proportion of *Bacteroidetes* and a lower proportion of *Proteobacteria* bound by IgA1 (Fig 4F). Family level analysis showed that reduced *Proteobacteria* was attributable to lower proportions of *Enterobacteriaceae* (Fig 4G and Appendix Fig S4H) which are known to include pathobionts and opportunistic species such as *Escherichia*, *Enterobacter*, and *Shigella* species normally present at very low levels in a healthy gut microbiota. Concurrently, increased *Bacteroidetes* levels were mainly due to higher *Bacteroidaceae* abundance in the IgA1 fraction (Fig 4F and Appendix Fig S4G).

Thirteen species were found differentially abundant between CD and Non‐IBD groups, and included *Bacteroides*, *Lachnoclostridium*, *Anaerostipes*, *Blautia*, and *Bifidobacterium* species (Appendix Table S13). Within these, two unknown *Bacteroides*, an *Anaerostipes*, a *Bifidobacterium* and a *Faecalibacterium* species, all known SCFA producers, were significantly more abundant in the IgA1^+^ fraction of CD patients' microbiota relative to non‐IBD (Fig 4H). In contrast, no specific enrichment was observed in the IgA1^+^ fraction of UC patients' microbiota compared with non‐IBD individuals. The UC IgA1^+^ fraction was, however, enriched in three *Ruminicoccaceae* species, two unknown *Anaerotruncus* and *Ruminiclostridium* species and *Massilioclostridium coli* compared with CD patients but these species did not pass our statistical abundance filters. It could be suspected that IgA1 might be detrimental in CD, possibly by binding species known to be beneficial.

### IgA2‐coated microbiota is partially preserved in IBD

A similar analysis in the IgA2^+^ fraction of non‐IBD and IBD microbiota again showed that ChaO1 and Shannon diversity indices of the IgA2^+^ fraction were not different between CD, UC, and non‐IBD (Fig 4H). PCA of the Jaccard distances did not illustrate any atypical clustering either (Fig 4I). At the phylum level, IgA2^+^ higher proportions of *Bacteroidetes* and lower proportions of *Proteobacteria* in CD patients were again observed (Fig 4J).

Opposite to IgA1, there were no significantly enriched *taxa* in the IgA2^+^ fraction of the stool microbiota in CD patients compared with non‐IBD individuals. In UC, however, one unknown species of *Bacteroides* was less abundant in the IgA2^+^ fraction relative to non‐IBD individuals (Fig 4K). Moreover, one *Lachnospiraceae* species was also less abundant in UC IgA2^+^ fraction relative to CD individuals (Fig 4K). While these observations concerned taxa known to include SCFA producers, IgA2 selection of the microbiota appears to be more robustly maintained in IBD than that of IgA1. Hence, while similar modification in the IgA1^+^ and IgA2^+^ fractions of IBD patients are observed at the phylum level (Fig 4F and J), IgA1^+^ bacteria appear more discriminating for CD versus non‐IBD comparisons.

### Neutralizing functions of IgA against pathogenic bacteria are reduced in IBD patients

**Figure 5:**
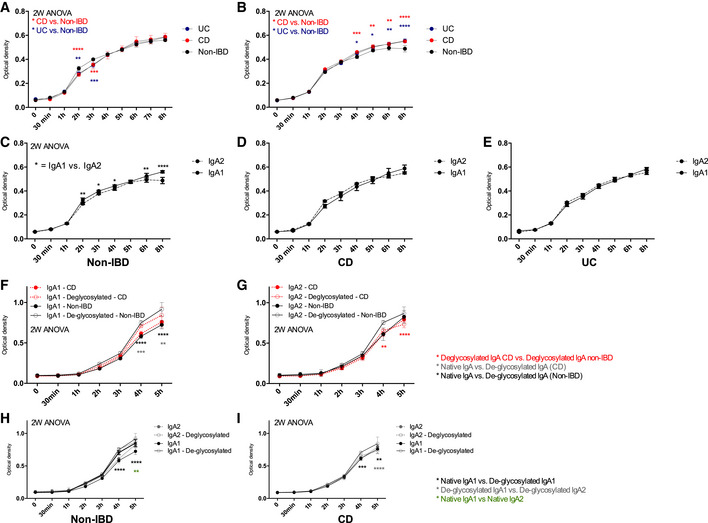
IBD IgA are less neutralizing than non‐IBD IgAs against pathogenic bacteria A–EOptical density (600 nm) variations during *in vitro* growth assay of *Salmonella enterica Typhimurium* (SL1344) co‐cultured with stool‐purified IgA1 (A) or IgA2 (B) from non‐IBD (*n* = 4), CD (*n* = 6) and UC (*n* = 3). Comparison between IgA1 and IgA2 coculture in non‐IBD (C), CD (D), and UC (E).F–IOptical density (600 nm) during *in vitro* growth assay of *Escherichia coli* (25,922 strain from ATCC) cocultured with stool‐purified IgA1 (F) or IgA2 (G) from non‐IBD (*n* = 2) and CD (*n* = 2). All patient samples (biological replicates) have been tested in technical duplicates. Comparison between IgA1 and IgA2 co‐culture in non‐IBD (H), and CD (I). These IgA samples were from the same patients throughout each experiment. Data information: Two‐way ANOVA with Holm–Sidak correction after D'Agostino–Pearson nomality test, *P*‐values are as follows: (A) UC curve: 2 h – *P* = 0.0024, 3 h – *P* = 0.003; CD curve: 2 and 3 h *P* < 0.0001 (B) UC curve: 4 h – *P* = 0.0186, 5 h – *P* = 0.0393, 6 h – *P* = 0.039, 8 h – *P* < 0.0001; CD curve: 4 h – *P* = 0.0001, 5 h – *P* = 0.0028, 6 h – *P* = 0.0024, 8 h – *P* < 0.0001. (C) 3 h – *P* = 0.0422, 4 h – *P* = 0.0422, 6 h – *P* = 0.0012, 8 h – *P* < 0.0001. (F) Gray curve: 4 h – *P* = 0.0004, 5 h – *P* = 0.0014; Black curve: 4 and 5 h – *P* < 0.0001. (G) De‐glycosylated CD vs. De‐glycosylated non‐IBD: 4 h – *P* = 0.0003, 5 h – *P* < 0.0001. (H) Native vs. de‐glycosylated: 4 and 5 h – *P* < 0.0001; De‐glycosylated IgA1 vs. De‐glycosylated IgA2: 5 h – *P* = 0.0012. (H) Native vs. de‐glycosylated: 4 h – *P* = 0.001 and 5 h – *P* = 0.006; De‐glycosylated IgA1 vs. De‐glycosylated IgA2: 5 h – *P* < 0.0001.

Polyreactivity confers broadly neutralizing abilities to IgAs (Bunker *et al*, 2017). Because gut microbiota selection appeared differential between IBD groups and compared with non‐IBD samples, we questioned whether IBD IgAs neutralizing abilities would be similar against pathogenic species. We thus cocultured *Salmonella typhimurium* (SL1344) with stool‐purified IgA1 and IgA2 from the same patients of our previous experiments (Fig 5). While IgA1 addition into culture medium did not seem to affect growth (Fig 5A and B), there was a small but strikingly significant increase in bacterial growth in both IBD groups relative to non‐IBD when using IgA2 (Fig 5B). Comparing IgA1 and IgA2 effect on bacterial growth within each group also showed that, while non‐IBD IgA2 were more neutralizing than IgA1 in the non‐IBD group (Fig 5C), this discrepancy was abrogated in both CD (Fig 5D) and UC (Fig 5E) groups. This suggests neutralizing, or at least bacteriostatic, capacities of IgA2 are dysfunctional in IBD.

This experiment has been then replicated on a commensal strain of *Escherichia coli* (25922 strain from ATCC), and after further de‐glycosylation of our patients' IgA. In both CD and non‐IBD groups, de‐glycosylated IgA1 were less neutralizing that native IgA1 (Fig 5F, H and I), albeit more strongly so for non‐IBD IgAs. Yet, there were no differences between CD and non‐IBD IgA1 (Fig 5F), whereas de‐glycosylated IgA2 from CD patients were more neutralizing than their non‐IBD counterparts (Fig 5G). In addition, de‐glycosylated CD IgA2 were still more inhibitory than de‐glycosylated CD IgA1 by the end of the kinetics (Fig 5I).

When taking into account previous results from this study, in our cohort, deglycosylation of CD IgA2 appears to impair the regulation of commensal growth, rendering IgA2 more bacteriostatic for commensals but not pathogens in CD.

### In CD, IgA2‐coated species correlate with disease activity

**Figure 6:**
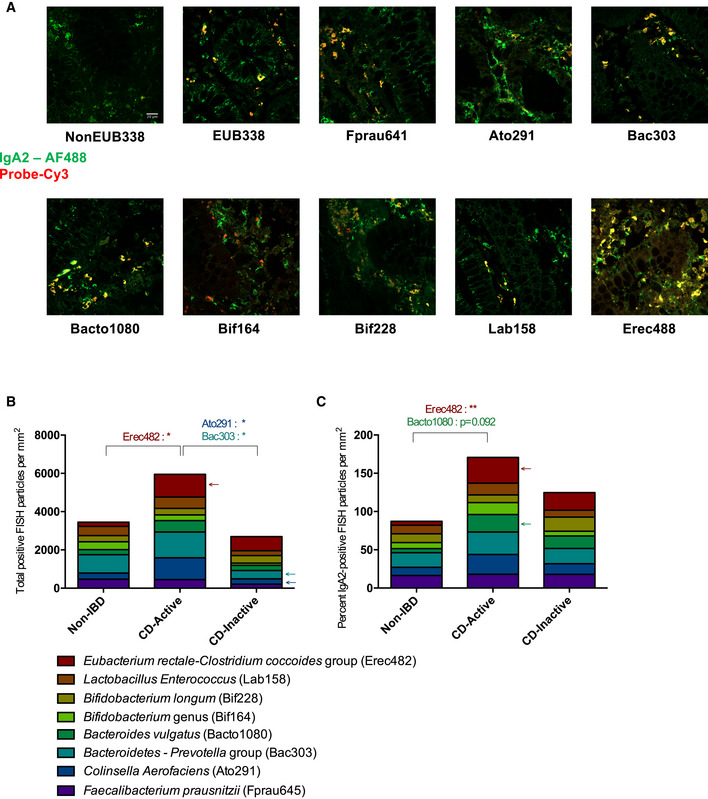
CD IgA2 display biased selectivity in active CD patients ACombined FISH‐IF staining for each probe.BTotal fluorescence detected per probe and per biopsy. Erec482 – *P = 0.00233; Ato291 – *P = 0.0268; Bac303 – *P = 0.0196.CPercent of co‐occurred IgA2 and FISH‐bound particles. Erec482 – *P = 0.0073. Data information: Two‐way ANOVA with Holm–Sidak correction after D'Agostino–Pearson nomality test. Non‐IBD: *n* = 3; Active CD: *n* = 6; Inactive CD: *n* = 4. All patient samples (biological replicates) have been tested in technical duplicates.

The absence of altered abundance in the IgA2‐bound fraction of the microbiota was surprising given the major role IgA2 plays in antigen sampling under homeostatic conditions and its dominance over IgA1 in the colon. More evidence also suggest mucus‐bound microbes might differ from stool bacterial species and differ along the digestive tract in specific ecological niche (reviewed in Wang *et al*, 2021). This led us to hypothesize that—if it did happen—local IgA2‐induced microbial dysbiosis might occur more locally, and possibly trigger immune pathways leading to flares while not participating in the overall maintenance of the inflammation. *In situ* IgA2‐microbe interaction by co‐staining colonic biopsies from CD patients with either active or quiescent disease with a panel of 10 previously published 16S rRNA probes was then evaluated (Appendix Table S14) and anti‐IgA2 fluorescent antibodies (Fig 6A). Due to the friability of their colonic mucosa, no stained biopsies could be obtained from UC patients. Quantitative analysis of the resulting images showed significantly higher abundance of *Eubacterium rectale‐Clostridium coccoides group* (Erec482) bacteria in biopsies of patient with active CD relative to non‐IBD biopsies (Fig 6B). This discrepancy in the Erec482 subgroup was also noted when analyzing co‐occurrence between IgA2 and 16S signals (Fig 6C), suggesting higher local binding of IgA2 to Erec482 bacteria in CD patients with active disease. Co‐occurred IgA2 and *Bacteroides vulgatus* (Bacto1080) also trended upwards in biopsies from active CD patients relative to non‐IBD counterparts (Fig 6B).

In addition, biopsies from patients with quiescent CD also displayed a lower total abundance of *Collinsella aerofaciens* (Ato291) and of bacteria from the *Bacteroidetes Prevotella* group (Bac303) relative to those with active disease (Fig 6B). However, co‐occurrence analysis did not statistically significantly recapitulate these observations, suggesting this is not an IgA2‐driven phenomenon (Fig 6C). Hence, while stool IgA2^+^ microbiota does not seem heavily impacted in our IBD cohorts, local defects related to SIg selection could still be associated with disease recurrence.

## Discussion

The present study meant to characterize the biological significance of IgA subclasses in relations to gut microbiota in a small cohort of IBD patients. However, our results even if remain preliminary concur with prior reports of IgA1‐ and IgA2‐associated selection of the microbiota (Sterlin *et al*, 2020). While these studies have investigated the relevance of IgA subclasses in healthy individuals and using serum IgAs, we explored these observations in dysbiosis conditions and looking specifically mucosal SIgAs. We have previously evidenced the contribution of genotype in promoting IgA RT in CD (Rochereau *et al*, 2021). Here, we further highlight distinct subclass‐dependent impairments of Ig selection and functionality in IBD.

Previous work on stool anti‐commensal IgA1 and IgA2 in healthy individuals showed that dual opsonization of the microbiota by IgA1 and IgA2 tends to segregate *Actinobacteria*, whereas IgA2 identification mostly promotes *Bacteroidetes* selection. Sterlin *et al* thus showed that IgA1 preferentially induces an IL‐10 oriented microenvironment, suggesting IgA1 promotes tolerance to commensals. A recent IgA‐seq study also characterized IgA‐bound microbes in IBD (Shapiro *et al*, 2021), efficiently demonstrating the predictive value of IgA‐bound microbiota over treatment efficacy and disease progression. *Lachnospiraceae*, *B. fragilis*, *Blautia*, and *Streptococcus* were also differentially IgA‐coated in IBD patients compared with healthy controls, consistent with prior results (Viladomiu *et al*, 2017). While it reported similar increases in *Actinobacteria*, *Bacteroidetes*, and *Proteobacteria* to previous studies (Gevers *et al*, 2014; Kostic *et al*, 2014), Shapiro *et al*’s elegant work did not uncover any difference between UC and CD in levels of IgA coating of the microbiota. Their analysis, despite covering a larger cohort, yielded significant results when IBD patients were combined in a single group and compared with healthy controls. Shapiro *et al* but also prior studies (Badr‐el‐Din *et al*, 1988; Leake, 2014; Viladomiu *et al*, 2017; Lin *et al*, 2018; Sterlin *et al*, 2020) concluded that host immune response to specific microbiota could be added to the combination of genetic susceptibility and environmental factors that are generally associated with IBD immunopathogenesis. Accordingly, we hypothesized that diving deeper into specific subclass‐associated functional and microbial discrepancies in IBD could be very useful. First, we show that CD SIgA have increased RT and increased reactivity toward common glycans. Moreover, the IgA1‐bound fraction of the microbiota is enriched in beneficial bacteria.Then, we also observed that UC SIgA do not undergo RT and display a significant loss of reactivity for common glycan structures.

In CD, a putative model could be proposed in which CD might be characterized by IgA1 gain of function, wherein IgA1 RT would promote increased bacterial antigen load in M cells. This would result in aberrant and unchecked immune responses toward beneficial taxa and possibly contribute to their elimination. Such pathogenic transport of IgA1 immune complexes is also evidenced in coeliac disease, although in a CD71‐dependent manner (De Palma *et al*, 2010; Papista *et al*, 2012). Alternative transport models for IgA1 are consistent with CD IgA1 being mostly hypo‐sialylated in our study (Fig 2A), a loss which was proven to increase effector function of agglutinated serum IgA1 (Steffen *et al*, 2020). Sialylations are generally immunosuppressive, and regulate IgA function under homeostatic conditions (Rochereau *et al*, 2013; Steffen *et al*, 2020; Sterlin *et al*, 2020) as they are key in M‐cell recognition of IgA‐bacteria complexes via the RT co‐receptor Siglec‐5. In our study, neither Dectin‐1 binding nor Siglec‐5 binding affinities for IgA1 were significantly different between non‐IBD and IBD group. Tendencies toward hypo‐sialylation in IBD IgA1 imply that CD IgA1 may not be recognized by Siglec‐5. This posits that RT of IgA1 in CD will not depend on Dectin‐1‐Siglec‐5 cooperation, as is the case for IgA2 (Rochereau *et al*, 2013). As such, there may either be an alternative Dectin‐1‐co‐receptor pairing or other IgA receptors may also be taken into consideration for IgA1 transport, such as CD71, FcαR, or Fcα/μR. Of note, it can be remarked that IgA1 and IgA2 share a region of partial sequence identity that contains a N‐glycosylation site: N144 for IgA1 and N131 for IgA2 that our experimental design based on Peptide‐M and Jacalin enrichment resolved.

AMIS in the gut indeed relies heavily on glycan–glycan interactions. Hence, commensals exhibit surface glycans that attract T‐independent SIg, to which extensive glycosylation confers a broad affinity for bacterial species (Pabst, 2012; Donaldson *et al*, 2018; Nakajima *et al*, 2018; Rollenske *et al*, 2018; Neumann *et al*, 2019; Sterlin *et al*, 2020). In addition, anchoring of beneficial bacteria by affinity for mucin glycans promotes symbiosis between commensals (Yamada *et al*, 2019). De‐sialylation itself may support the growth of pathobionts of the microbiota, as increased sialidase activity is known to promotes dysbiosis and gut inflammation (Huang *et al*, 2015b), as well as correlates with lower microbial diversity and microbial translocation (Giron *et al*, 2020). The de‐sialylation of CD IgA1 may thus originate from increased sialic acid catabolism as a consequence of dysbiosis, and finally alter IgA function resulting in an aberrant selection of the microbiota. Analysis of sialic acid catabolism in patients with active disease may then provide a link between IgA glycosylation and microbial dysbiosis in IBD. De‐sialylation of IgA1 in IBD may thus affect the recognition of commensals themselves and is consistent with enrichment of beneficial *Faecalibactierum* in the IgA1^+^ fraction of our CD patients' microbiota (Fig. 4G). Indeed, loss of *Faecalibacterium*, especially *Faecalibacterium prausnitzii*, is critically associated with CD and its severity (Joossens *et al*, 2011; Machiels *et al*, 2014; Yilmaz *et al*, 2019). *Anaerostipes*, *Bifidobacterium*, and *Peptostreptococcaceae*, which were also enriched in our CD IgA1^+^ fractions, are also less abundant in the microbiota of treatment naïve CD patients (Gevers *et al*, 2014) and we suspect their loss might also be attributable to pathogenic IgA1 selection. In addition, decreased *Proteobacteria* selection by CD IgA1 (Fig 4F) and IgA2 (Fig 4J) suggests opportunistic species might be allowed to expand in CD. This also coincides with previous findings that *Proteobacteria* abundance generally increases in CD (Vester‐Andersen *et al*, 2019). Thus, we suggest that IgA1 binding might be pathogenic in CD and could promote elimination of promoters of symbiosis in the microbiota.

Interestingly, this contradicts a recent report that found no extensive shift in IgA reactivity for specific members of the microbiota in CD (Kabbert *et al*, 2020). However, subclasses of human IgA were not considered in this study. Of note, the correlation between IgA1^+^ bacteria frequency with disease duration in our study (Appendix Fig S3D and E) suggests this IgA1 defect is a phenomenon that accumulates over years. Yet, as flow cytometry analysis showed that IgA1 was mostly found in dual binding with IgA2 (Fig 4A–C), a large portion of our magnetically isolated IgA1^+^ fraction may actually be IgA1^+^ IgA2^+^. Given this separation still gets rid of the IgA1^−^IgA2^+^ fraction, IgA1 binding, even concurrent with IgA2, seemingly effectively shifts commensal selection in our CD patients. In our IgA2^+^ fractions, few discrepancies in bacterial abundance were noted between CD and non‐IBD samples, suggesting the eliminated IgA1^+^ IgA2^−^ fraction of the microbiota might still be a driving parameters behind our observations.

Changes in IgA2 biology and functionality were also observed. In healthy individuals, IgA2 tend to favor the selection of terminal αGal and Neu5Ac motifs (Sterlin *et al*, 2020), which was not the case either in the CD or in the UC cohorts. Increased targeting of maltotetraose by CD IgA2 suggests IgA2‐driven dysregulation of glucose metabolism at the mucosal level (An *et al*, 2005). In addition, maltotetraose also inhibits TNF‐α‐induced expression of intercellular adhesion molecule‐1 (ICAM‐1; Shin *et al*, 2016). In a mucosal context, gut neutralization by IgA2 could amplify TNF‐α signaling and immune effectors recruitment during CD, thusly feeding an already strong inflammatory loop. The rest of these antigens were natural oligosaccharides, blood antigens, or milk polysaccharides, suggesting both altered self‐reactivity and reactivity toward dietary antigens, which could align with observation of auto‐immune features in CD. To further study the role of CD IgA2 in microbiota selection, we have also co‐stained IgA2 and various bacterial 16S rRNA probe in CD patients' biopsies with active and inactive disease. While IgA2^+^ stool microbiota did not appear to vary significantly relative to non‐IBD, we observed increased local co‐occurrence between IgA2 and the Erec382 probe in active disease biopsies (Fig 5). The Erec482 probe detects about one‐third of the total human microbiota as it identifies most members of *Clostridium* group XIVa (Franks *et al*, 1998). This group includes *Eubacterium rectale*, a species known—amongst several other *Lachnospiraceae*—to be less abundant in CD (Yamada *et al*, 2019). IgA2 selection might thus influence dysbiosis‐associated disease recurrence. This suggests two distinct events affecting IgA‐microbe interactions: (i) fecal IgA1, possibly through de‐sialylation aberrantly eliminates beneficial commensals in the gut lumen while (ii) tissular IgA2 limits the establishment of symbiotic niches in the mucus. In CD, modification of IgA1 glycosylation may also affect its transport in the mucosa, and combine with IgA2‐mediated immune responses against commensals. In addition, previous results on IgA‐microbiota interactions focused on the level of IgA levels of bacteria coating, as a predictive marker of colitogenic species (Palm *et al*, 2014; Shapiro *et al*, 2021). Our study did not survey this parameter, as we sorted microbes according to the nature of the IgA, and the specific extent to which IgA1 and IgA2 specifically coat the IBD microbiota remains unknown to date.

In UC, a distinct model in which IgA lose their effector functions could be also proposed, as evidenced by almost nonexistent RT (Fig 1H) and substantially lower anti‐glycan reactivity of both UC IgA1 and IgA2 (Fig 3A and B). Changes in Ig‐microbiota interaction were less obvious than in CD, despite distinct profiles from CD (Fig 4). Contrary to CD, in UC, SIg are synthesized but not secreted into the mucosa, or at least at a lower rate (MacDermott *et al*, 1981; Badr‐el‐Din *et al*, 1988; Klemola *et al*, 1988). The loss of reactivity for UC IgA, regardless of subclass, demonstrates that what little IgA manages to reach the intestinal lumen may have reduced functionality, distinctive from our observations in CD. Lack of IgA‐induced selection would prevent the elimination of opportunistic species but also the maintenance of beneficial ones. In addition, whatever microbiota manages to be selected would not be transported into the PP via Dectin‐1 binding, meaning that no ensuing response would be built to discriminate bacterial strains in UC. In this context, it would be interesting to study potential compensatory bacterial selection by SIgM of IgG in the microbiota of these patients. SIgM indeed partially compensates for defective or absent IgA in mouse models of IgA deficiency and in human cohorts of selective IgA deficiency (Fadlallah *et al*, 2018, 2019; Catanzaro *et al*, 2019; Michaud *et al*, 2020; Sterlin *et al*, 2020). IgG coating is also particularly evidenced as a biomarker of IBD, and so the dynamic relationship between IgA‐ and IgG‐dependent would be of particular interest (Lin *et al*, 2018; Rengarajan *et al*, 2019).

**Figure 7:**
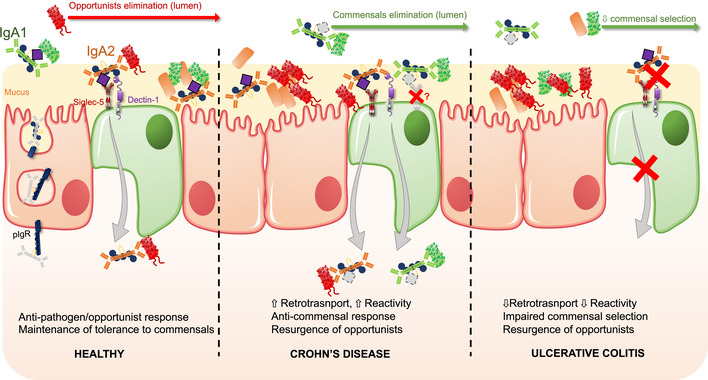
Putative model for altered subclass‐dependent selection of the microbiota by IgA in IBD While in the healthy gut, IgA1 have mostly neutralizing and/or bacteriostatic properties, IgA2 undergoes reverse‐transcytosis via Dectin‐1 and Siglec‐5 to deliver antigens to the immune cells of the PP. This mechanism is dependent on glycosylations, notably sialylations for Siglec‐5 recognition. This allows for efficient elimination of pathogens and opportunists. IgA‐mediated entrapment of commensal bacteria into the mucus is also glycan‐dependent, wherein bacteria, IgA and mucin glycosylation are required. In CD (middle panel), luminal binding of commensal by‐ and RT of IgA1, would promote higher antigen load to the *lamina propria* and establishment of responses against both commensals and opportunists, which would ultimately favor opportunist's growth. Mechanisms of IgA1 retro‐transport remain to be determined. In addition, de‐sialylation of IgA1 limits its effector function, and would affect adequate commensal selection. Tissular IgA2, in turn, is less efficient at neutralizing opportunists, with increased binding to commensals during active disease, which would favor opportunists again. In UC (right panel), absence of RT for IgA2 and overall loss of anti‐glycan reactivity would lead to impaired tolerogenic responses to commensals but also impaired responses against pathogens and opportunists, resulting in opportunist's resurgence within the microbiota.

In the gut, pathogens and opportunists are selected through high‐affinity T‐dependent IgA and eliminated through clump formation (Moor *et al*, 2016; Okai *et al*, 2016; Bansept *et al*, 2019; Fujimoto *et al*, 2019; Hoces *et al*, 2020). However, contradictory evidence suggests that accumulation of somatic mutation, rather than polyreactivity, enforces antibody–microbiota interactions in the gut of both healthy and CD individuals (Kabbert *et al*, 2020); hence, this model remains discussed. Here, we only evaluated broad IgA reactivity and did not distinguish affinity‐matured from polyreactive IgA populations. Evaluating the strength of the overall IgA response in the gut of IBD patients would thus require a more in‐depth study of the mucosal IgA repertoire in IBD. It would also be important to correlate IgA1‐ and IgA2‐microbiota interactions at specific locations of the digestive tubes, especially in IBD, given the various disease phenotypes and given there are distinct ileum‐associated and distinct colon‐associated repertoires in the healthy gut (Fenton *et al*, 2020). This could indicate specifically adapted antigen selection corresponding to differential colonization of the mucosa by site‐specific microbiota (Cheng *et al*, 2019), which might be disrupted in IBD. Indeed, the small ileum and jejunum and PPs are enriched in IgA1^+^ B cells while the terminal ileum and colonic *lamina propria* mostly host IgA2^+^ B cells (Chiba *et al*, 1987; He *et al*, 2007). Of note, circulating IgA2^+^ B cells display high expression of gut homing integrins α4β7 compared to IgA1^+^ counterparts (Pakkanen *et al*, 2010). To add, recent data suggest IgA1 and IgA2 convergence for microbiota binding in the ileum, whereas IgA2 dominates and targets several genera, especially *Bacteroidetes*, in the colon of healthy individuals (Sterlin *et al*, 2020). This proposes that the colon is a site of IgA2 responses while the small intestine elicits both IgA1 and IgA2 responses. On a deeper level, and given our results, there may be fundamental changes in the diversification of B‐cell repertoires and their homing dynamics along the digestive tube of IBD patients, which may preclude commensal dysbiosis and could be interesting biomarkers for early IBD diagnosis. On the basis of this, a putative model for a hypothesis of tissular versus luminal functions of IgA subclasses in IBD is proposed in Fig 7. Yet, there is definite ground to suspect IgA subclass has biological significance in microbiota‐induced immune polarization at mucosal surface; and that impairments in IgA post‐traductionnal handling, uptake, or selection, may promote microbial instability in IBD. This is of particular importance when considering the rising number of studies and clinical trials investigating fecal microbiota transplantation (FMT) as a curative option for IBD dysbiosis. Fecal microbiota transplantation outcomes remain heterogenous, with various approaches for mode of administration, donor graft composition, and patient history that blur comparisons (Allegretti *et al*, 2017; Bak *et al*, 2017; Keshteli *et al*, 2017; Benech & Sokol, 2020). Still, the vast majority of these studies do not look into pre‐existing IgA coating stool microbiota in both donor and receiver, when it could very well predict engraftment success. These findings thus offer new directions on the design of novel therapeutic strategies such as FMT, or treatments aimed at specifically blocking IgA RT to lower the inflammatory burden during acute episodes.

## Materials and Methods

### Patient selection

Eligible IBD patients were followed in our IBD Center for CD and UC. All included patients were treated with a first anti TNF drug in maintenance phase and without any prior treatment optimization. Patients treated with other treatments or in combination with immunosuppressive drugs were excluded. Informed consent was obtained for each included patients, which was approved by the Ethics Committee Board of Saint‐Etienne Hospital and the Centre National Informatique et Liberté (CNIL; Number: 1849323). Consent and experiments hereafter performed comformed to the WMA Declaration of Helsinki the experiments and to the principles set out in the Department of Health and Human Services Belmont Report. Colonoscopies were performed to measure endoscopic activity as internationally recommended or to detect mucosal healing conversely in patients in clinical remission as recommended in clinical practice. Two biopsies at least were performed in inflamed tissues in patients without mucosal healing and systematic biopsies in non‐inflamed tissue in patients with mucosal healing. Twenty‐nine patients (17 CD and 12 UC) were included. Non‐IBD patients were patients who came for a colonoscopy to diagnose cancer and whose colonoscopy did not reveal any lesion or inflammatory syndrome. The biological workup of these negative controls is totally normal. At least two biopsies were performed in normal tissue. Patients with an indication of colonoscopy for other reasons were excluded in this group. We defined active or inactive disease according to endoscopic scores. Thus, we defined an endoscopic activity for CD with an SESCD score > 5 for ileocolic or colic CD or > 3 for ileal CD; for UC, endoscopic activity was associated with an endoscopic mayo score > 1. When data on disease activity were not available, patient data were not included in the graphs. Clinical data of the cohort are available in Appendix Table S1. All colonoscopies were performed by the same endoscopist (XR).

### Stool samples

Stools from CD, UC, or healthy individuals were collected prospectively from informed and consenting non‐IBD, CD, and UC patients (Centre National Informatique et Liberté CNIL, approbation number: 1849323; Ethic committee of the CHU Saint‐Etienne, Terre d'Ethique) then transferred to our facility. Briefly, samples were homogenized in Phosphate‐Buffer Saline (PBS)‐Tween 0.5% containing protease inhibitors (HaltProtease inhibitor cocktail, ThermoFisher, 78429) at a 0.1 g/ml concentration then centrifuged at 1,500 *g* at 4°C for 20 min. Supernatant was collected then centrifuged at 10,000 *g* at 4°C for 10 min. Supernatant was again collected, aliquoted, then stored at −20°C. Pellets were suspended in 3 ml of PBS‐Tween 0.5% and stored at −20°C for magnetic cell separation.

### IgA and SC ELISAs

Nunc Maxisorp 96‐well plates (Fisher) were coated with either anti‐human IgA antibodies (Sigma I1261) or mouse anti‐human SC (courtesy of B. Corthésy) in carbonate buffer (pH 9.6). Plates were sealed and incubated overnight at 4°C. Wells were saturated with BSA 1% in PBS‐tween 0.05% (PBST) at room temperature (RT) for 90 min, then washed three times with PBST; stool samples were added at a 1/100 first dilution and incubated at RT for 1 h 30 min. Plates were then washed with PBST, then rabbit anti‐human SC (courtesy of B. Corthésy), or biotinylated anti‐human IgA1 (Southern Biotech, 9130–08) or anti‐human IgA2 (Southern Biotech, 9140–08) were incubated at RT for 1 h 30 min. After washing in PBST, streptavidin‐HRP (BD, 554066) or anti‐rabbit IgG‐HRP (ThermoFisher, G‐21234) were added for 1 h. Revelation through addition of TMB in wells for 3–10 min was stopped with 1 N HCl. Plates optical density (OD) were read at 450 nm (Sunrise TECAN microplate reader).

### IgA1 and IgA2 purification

IgA purification were performed as described by Moor *et al* (2016). Briefly, a 10 ml aliquot of homogenized stool was thawed, filtered on a 20 μm filter, and loaded on a peristaltic pump connected to a Peptide‐M‐Agarose (Invivogen) filled column (ref C10/10, GE Healthcare) and left running on a closed loop at 4°C overnight. Bound IgA was eluted on an AktaSTART HPLC (GE Healthcare) with pH 2–3 0.1 M Glycine. The resulting eluate was neutralized with pH 7.5 Tris–HCl 1 M, and then placed to run on a Jacalin‐Agarose (Pierce) column. Elution was carried out using α‐D‐Galactose, 1 M (Sigma). Samples were then loaded onto 50 kDa Amicon (ThermoFisher) and buffer‐exchanged in PBS 1X for storage at −20°C in low retention tubes (ThermoFisher). IgA1 purification consistently yielded lower IgA1 concentrations than IgA2, possibly because IgA1 is less abundant in the colon. This limited the number of experiments that could be performed with one batch of stool‐purified IgA1 and the number of patient that could be included in some experiments. UC patients generally had low IgA concentrations that also limited the final amount of purified IgA and thus the number of patient's sample available for use in certain experiments. Throughout this study, the same patients' IgA1 and IgA2 were used, although some samples had to be excluded due to low concentrations (this is specified in each of the procedure description). No other patient's sample from the cohort that had not been previously experimented with was used to compensate whenever concentrations were too low. This was made to avoid working with heterogenous sample groups between experiments, which would limit the consistency of the results.

### *In vitro* model of human FAE and reverse‐transcytosis assay

Six non‐IBD IgA1 and IgA2, 7 CD IgA1and 11 CD IgA2, 4 UC IgA1 and 6 UC IgA2 were purified from stool and RT potential was evaluated *in vitro*. The model was set up as previously published (Rochereau *et al*, 2013). Briefly, transwells inserts (Corning, 1402) were reversed and 300 μl of Matrigel‐enriched DMEM high‐glucose medium was incubated at 37°C for 30 min. After removal of excess liquid, 3.3 × 10^5^ Caco2 cells were seeded on the still reversed insert and incubated at 37°C overnight. Inserts were then placed in 12‐wells plates and both inside and outside chambers were filled with complete DMEM high‐glucose medium and cultured for 7–9 days. When trans‐epithelial resistance (TER) reached about 350 Ohms, 1 × 10^5^ Raji cells were added to the inner chamber and the culture was monitored for a 100–150 Ohm TER drop over the next 2–4 days. Once ready, inserts were fitted in a silicone tube and both top and bottom chambers were filled with HBSS buffer. Samples were added to the top chamber and incubated for 90 min at 37°C. The bottom chamber was collected and tested for Ig passage by ELISA.

### IgA binding assay to Dectin‐1 and Siglec‐5

From the samples used *in vitro* in the FAE model, 6 non‐IBD IgA1 and IgA2, 10 CD IgA1 and IgA2, 4 UC IgA1 and 5 UC IgA2 were tested for Dectin‐1 binding ability. Recombinant Fc‐Dectin‐1 and Fc‐Siglec‐5 (R&D Systems) was coated overnight in carbonate buffer at room temperature in Nunc Maxisorp 96‐wells plates (Fisher). Concentrations were adjusted so that purified IgA could be used at a 10 receptors per 1 IgA ratio later on. Wells were washed three times with PBST, and stool‐purified IgA was added to the wells and incubated 2 h with slight agitation at RT. After three washes with PBST, wells were incubated with biotinylated anti‐human IgA1 (Southern Biotech, 9130–08) or anti‐human IgA2 (Southern Biotech, 9140–08) diluted 1/2,500 and incubated at RT for 90 min. After washing in PBST, streptavidin‐HRP (BD, 554066) was added for 60 min. Revelation through addition of TMB in wells for 3–10 min was stopped with 1 N HCl. Plates optical density (OD) were read at 450 nm (Sunrise TECAN microplate reader). After blank subtraction, only ODs higher than 0.250 were considered strong binding and ODs below were considered weak Dectin‐1 binding.

### Bio‐Layer interferometry

Measurements were performed on the BLItz instrument (FortéBio) using Dip and Read™ anti penta‐HIS (HIS1K) biosensors (FortéBio). Biosensors were hydrated with PBS for 10 min, then placed on the reading tip of the BLItz and incubated 30 s with PBS (initial baseline). Next, biosensors were loaded with 4 μl of His‐tagged Dectin‐1 (R&D Systems, 1859‐DC‐050) for 120 s. After the loading step biosensors were incubated with PBS for 30 s (second baseline), then incubated with 4 μl of stool‐purified IgA1 for 120 s. Finally, Dectin‐1 and IgA1 were dissociated following incubation of biosensors in PBS. Biosensors were discarded after each measurement.

### IgA reactivity against glycans

To evaluate IgA polyreactivity in the samples, we had experimented with, glycan arrays (Raybiotech) were used according to the manufacturer's instruction. Arrays were blocked for 30 min at room temperature, then stool‐purified IgA1 (4 non‐IBD, 4 CD, and 4 UC) or IgA2 (6 non‐IBD, 6 CD, and 6 UC) were incubated overnight at 4°C a final concentration of 50 μg/ml. After washing the slides, biotinylated mouse anti‐human IgA1 or IgA2 (Southern Biotech) diluted 1/500 were incubated at room temperature under slight agitation for 2 h. Slides were washed, then the Streptavidin‐Cy3‐equivalent reagent (Raybiotech) was added for 1 h, after which slides were washed, air‐dried, and stored at −20°C until Cy3‐fluroescence readings and statistical analysis could be performed (Tebu‐bio). For analysis, log2‐transformed signal intensities were standardized (centering and scaling) by subtracting the average and then dividing by the standard deviation as per the manufacturer's recommendations. The Limma R package (The Linear Models for MicroArray) was used to run statistical analysis to compare the variance of biomarkers across groups.

### Glycan profiling and structural characterization by mass spectrometry

#### Sample preparation and protein digestion

IgA1 and IgA2 purified from 3 CD patients and 1 UC patient, stored at −20°C in PBS 1×, were loaded onto a Microcon 10 kDa (Merck Millipore) and exchanged against deionized water. Samples were reduced with 10 mM dithiothreitol (Sigma) for 30 min at 55°C and alkylated with 15 mM iodoacetamide (Sigma) for 30 min in the dark at room temperature. IgA were then digested with sequencing grade trypsin (Promega; enzyme to substrate ratio of 1:50) in 50 mM ammonium bicarbonate buffer, overnight at 37°C with gentle shaking. Tryptic digests were desalted and concentrated using pipette tips packed with reversed‐phase material (Zip‐Tip C18 units, Merck Millipore). Peptides were eluted in a solution of 70% acetonitrile with 0.1% trifluoroacetic acid and then diluted in an aqueous solution with 0.1% formic acid for subsequent nanoLC‐MS/MS analysis.

#### nanoLC‐MS/MS analysis

Peptides were analyzed using an Ultimate 3000 nanoLC system coupled with a Orbitrap Fusion™ Lumos™ Tribrid™ Mass Spectrometer (Thermo Fisher Scientific). One μg of trypsin‐digested IgA was loaded onto a reversed‐phase C_18_ PepMap™ trap column (300 μm‐inner diameter × 5 mm) (Thermo Fisher Scientific) at a flow rate of 10 μl/min before peptides separation on an analytical 75 μm id × 50 cm C18 Pep‐Map column (Thermo Fisher Scientific) with a 5–50% linear gradient of solvent B in 105 min (solvent A: 0.1% formic acid in water; solvent B: 0.1% formic acid in 80% ACN). The separation flow rate was set at 300 nl/min. The mass spectrometer operated in positive ion mode at a 1.8‐kV needle voltage. Data were acquired using Xcalibur 4.3 software in a data‐dependent mode. Full MS were recorded from m/z 350 to m/z 1,800 with orbitrap resolution of 120,000 (@ m/z 200), automatic gain control of 4 × 10^5^ and 275°C capillary temperature.

To characterize the glycosylation patterns, Higher‐energy Collision Dissociation (HCD) and Electron‐Transfer Higher‐energy Collision Dissociation (EThcD) were used. Glycan oxonium ions resulting from HCD MS/MS experiments (e.g., HexNAcHex oxonium ion at m/z 366.140, HexNAc at m/z 204.087, HexNAc fragments ions at m/z 186.076 and m/z 138.055) were used as diagnostic ions to screen the occurrence of glycoproteins and start EThcD acquisition. The fragmentation started from highest charge state and +2 to +8 charged ions were selected. The following MS/MS settings were used: dynamic exclusion during 60 s, normalized HCD collision energy of 28%, resolution 30,000, scan range m/z 120–2,000, isolation window 3 m/z, MSn automatic gain control target: 4 × 10^5^ ions collected during 60 ms.

#### Data processing

Data processing was performed using SEQUEST Proteome Discoverer 2.3 (Thermo Fisher Scientific) against *Homo sapiens* database (Uniprot) for protein identification. The search parameters were: mass tolerance set to 10 ppm and 0.02 Da, respectively, for MS and MS/MS modes, methionine oxidation (+15.995 Da) and N‐terminal acetylation (+42.011 Da) set as variable modifications, and cysteine carbamidomethylation (+57.021 Da) set as fixed modification, two missed cleavages allowed.

Byonic's Glycopeptide Search (Byonic™ 2.6 in Proteome Discoverer, Protein Metrics Inc., San Carlos, CA) was used to characterize the glycoforms against a custom database including Human Ig alpha 1 heavy chain, Ig alpha 2 heavy chain, Immunoglobulin J chain and Polymeric Immunoglobulin Receptor sequences. The search parameters were set as follows: 10 and 20 ppm mass tolerance for MS and MS/MS, respectively, cysteine carbamidomethylation set as fixed modification, methionine oxidation and N‐terminal acetylation as variable modifications. Glycopeptide false discovery rate was 1%. Two internal databases of N‐linked and O‐linked glycans containing 182 common human N‐glycans and 70 human O‐glycans respectively were used during automatic glycan search. Both peptide sequence and glycan compositions were used for glycoprotein identification.

### MACS purification of SIgA1 and SIgA2

From the same stool samples as those used for antibody purification, ELISA and *in vitro* experiments, 4 non‐IBD stools, 7 CD stools, and 4 UC stools were homogenated as described above. Resuspended pellets from stool homogenization were divided into two 1.5 ml fractions. Each were centrifuged at 10,000 *g* for 6 min and washed twice in MACS buffer (Miltenyi). Pellets were resuspended in 500 μl of MACS buffer and incubated with biotinylated anti‐IgA1 or anti‐IgA2 Abs (Southern Biotech) at 4°C with slight agitation for 40 min. After two washes, they were resuspended in 500 μl. To which 50 μl of magnetic streptavidin beads (Miltenyi) were added, and incubated at 4°C for 20 min. Magnetic separation was then performed on LS columns with a MACS Magnet according to the manufacturer's instruction. Eluted fractions were pelleted at 10,000 *g* for 10 min, and pellets were stored at −20°C for 16S‐sequencing.

### Analysis of IgA‐coated microbiota by flow cytometry

Of 7 non‐IBD stool homogenates, 17 CD homogenates and 12 UC homogenates, 2 ml of stool supsensions were thawed and centrifuged at 10,000 *g* for 6 min. Pellets were washed twice in 1 ml FACS buffer (PBS 1×, 2% FBS, 10 mM EDTA) then stained with mouse anti‐human IgA1‐PE (Southern Biotech, 9130–09) and mouse‐anti‐human IgA2‐AlexaFluor647 (Southern Biotech, 9140–31) at 4°C in the dark for 30 min. Cells were washed in FACS buffer once, then re‐suspended in 300 μl of FACS buffer. Samples were then analyzed on a BD FACS Canto II cytometer (BD Bioscience) and resulting data were analyzed using the FlowJo Software (Appendix Fig S1).

### Analysis of the IgA1‐ and IgA2‐coated microbial communities using sequencing

Extraction of total bacterial DNA from purified IgA1 and Iga2 was performed using the QIAamp PowerFecal DNA Kit (Qiagen) and the mechanical bead‐beating disruption method as previously described (Lemaire *et al*, 2018). The V3‐V4 region of the 16S rRNA genes was amplified using MolTaq (Molzym, Plaisir, France) and primers V3F: TACGGRAGGCAGCAG and V4R: ATCTTACCAGGGTATCTAATCCT. The purified amplicons were sequenced using Miseq sequencing technology (Illumina) at the GeT‐PLaGe platform (Toulouse, France). Paired‐end reads obtained from MiSeq sequencing were analyzed using the Galaxy‐supported FROGS (Find, Rapidly, OTUs (Operational Taxonomic Units) with Galaxy Solution) pipeline (Escudié *et al*, 2018). For preprocessing, reads with length ≥ 380 bp were kept. Clustering and chimera removal steps followed the FROGS guidelines. Assignation was performed using SILVA 16S 128. OTUs with abundances lower than 0.005% of the total read set were removed prior to analysis. Then, 16S sequencing data were analyzed using the Phyloseq, DESeq2 and ggplot2 R packages in addition to custom scripts as previously described (Safari *et al*, 2020). Samples were rarefied to even sampling depths (3,187 sequences per sample) before computing within‐samples compositional α‐diversity and between‐samples compositional β‐diversity. Principal coordinate analysis was performed as previously described (Safari *et al*, 2020) using Jaccard and Bray–Curtis indices. A permutational multivariate ANOVA test was performed on the matrices using 9,999 random permutations and at a significance level of 0.01. Raw, unrarefied OTU counts were used to produce relative abundance graphs and to find taxa with significantly different abundances in Non‐IBD, UC, and CD groups, using DESeq2. Values of *P* were corrected for multiple testing using the Benjamini–Hochberg procedure to control the false discovery rate (FDR) as previously described (Burz *et al*, 2021). The 16S datasets produced in this study are available in the following databases: [16S rRNA sequences]: [Portail Data INRAE] [https://doi.org/10.57745/BOUNLH].

### *In vitro* salmonella and *E. coli* neutralization assay

SL1344 *Salmonella enterica* Typhimurium isolates were provided by Dr. Blaise Corthésy. After a 24 h culture at 37°C on streptomycin agars (90 μg/ml), 5% CO_2_, one colony was transferred to 3 ml of liquid LBstrep broth (Streptomycin 90 μg/ml) for another 24 h at 37°C, 5% CO_2_ and under slight agitation. Approximately 100 μl of the resulting culture were diluted into another 3 ml of liquid LBstrep broth, to get to a 0.1 optical density (OD600nm). One hundred and fifty microlitre per well of the suspension were seeded into 96‐well plates and IgA1 or IgA2 from non‐IBD (*n* = 4), CD (*n* = 6) and UC (*n* = 3) at a final concentration of 6 μg/ml and cocultured for 24 h. *Escherichia coli* isolates (25,922 strain from ATCC) were cultured for 24 h at 37°C, 5% CO_2_, on Columbia agars with 5% sheep blood. One colony was transferred to 3 ml liquid LB broth for another 24 h at 37°C, 5% CO_2_, under slight agitation. One hundred and fifty microlitre of the resulting culture were diluted into another 3 ml of liquid LB broth to get to a 0.1 optical density (OD600nm). One hundred and fifty microlitre of the suspension were seeded into 96‐well plates with IgA1 from non‐IBD (*n* = 2) or CD (*n* = 2) or with IgA2 from non‐IBD (*n* = 2) or CD (*n* = 4) at a final concentration of 6 μg/ml and cocultured for 24 h. Deglycosylation of patients IgA was performed using the Enzymatic CarboRelease™ Kit from QA‐Bio®. N‐glycosylations from IgA1 and IgA2 were removed using PNGase while O‐glycosylations from IgA1 were removed using an enzyme cocktail composed of O‐glycosidase, neuraminidase, β‐galactosidase and β‐N‐acetylglucosaminidase. IgA were incubated with the enzymes for 24 h at 37°C, according to the manufacturer's instructions. Optical density measurements were taken hourly at 600 nm on a Sunrise TECAN microplate reader.

### Combined FISH‐IF staining

Biopsies from six active and four inactive CD patients were harvested during routine exploratory colonoscopy and immediately placed in complete cold RPMI medium for transport. They were fixed in 4% paraformaldehyde overnight then embedded and frozen in OCT. Six micrometre sections were cut, then rinsed in PBS three times to eliminate OCT. Tissue permeabilization was performed by incubating slides in 0.6% Triton for 5 min, then incubated in 2× Saline Sodium Citrate (SSC) buffer for 25 min. Slides were washed in DEPC‐treated water (10 dips), then in 0.065 M trietanolamine (TEA, Sigma; 10 dips), and finally twice in 2× SSC. Tissues were hybridized with 150 μlof 16S‐probes (50 pM final; Appendix Table S2) overnight, at room temperature. Slides were washed in successive baths of decreasing SSC concentrations (4×, 2×, 1×, 0.5×, 0.1×) then blocked for 1 h in a 1% BSA, 5% FBS, 0.3% Triton‐X100 PBS solution. All subsequent washing steps were carried out in PBS. Mouse anti‐human IgA2 antibodies (Southern Biotech, 9140–01) were deposited over each section at a 1/500 dilution and incubated for 2 h at room temperature. Slides were washed and secondary antibodies (goat anti‐mouse IgG‐AF488, abcam) were then added for 1 h. Slides were washed in PBS then distilled water, air‐dried, and sealed with Vectashield (Vectra, H1000) for confocal microscopy.

### Statistical analyses

Unless otherwise specified, statistical analyses were performed using the InStat version 2.01 from the GraphPad Software. A nonparametric Mann–Whitney *U*‐test or Kruskal–Wallis test followed by Dunn's correction *post hoc* test were used when datasets failed d'Agostino‐Pearson normality test or if most sample values in a dataset failed normality tests. The limit of significance for *P*‐values was set at 0.05 (marked by * in the plot); ** indicates *P*‐values ≤ 0.01, and *** stands for *P*‐values ≤ 0.005. Statistically significant differences between groups are emphasized by bars connecting the relevant columns under comparison. All experiments were performed using each patient's sample as a biological replicate that were used in technical duplicates, and triplicates when possible; the experiments were not performed in a blinded setting.

## Author contributions

**Eva Michaud:** Formal analysis; validation; investigation; methodology; writing – original draft. **Louis Waeckel:** Formal analysis; investigation; writing – original draft. **Rémi Gayet:** Formal analysis. **Blandine Chanut:** Formal analysis; investigation. **Fabienne Jospin:** Formal analysis; investigation. **Katell Bathany:** Formal analysis; investigation; writing – original draft. **Magali Monnoye:** Formal analysis; investigation; writing – original draft. **Coraline Genet:** Investigation. **Roman Goguyer‐Deschaumes:** Investigation; writing – review and editing. **Amelie Prier:** Investigation. **Caroline Tokarski:** Formal analysis; investigation; writing – original draft. **Philippe Gerard:** Formal analysis; supervision; investigation; methodology; writing – original draft. **Xavier Roblin:** Formal analysis; supervision; validation; investigation; methodology; writing – original draft. **Nicolas Rochereau:** Formal analysis; supervision; investigation. **Stéphane Paul:** Conceptualization; data curation; formal analysis; supervision; funding acquisition; validation; investigation; methodology; writing – original draft; project administration; writing – review and editing.

In addition to the CRediT author contributions listed above, the contributions in detail are:

EM, LW, RG‐D, RG, BC, FJ, KB, MM, CG, LW, AP, CT, PG, XR, NR, and SP performed the research; SP, EM, and NR designed the research study. EM, NR, XR, and SP analyzed the data. EM and SP wrote the paper. All authors approved the final version of the manuscript.

## Disclosure and competing interests statement

Pr Roblin was speaker for MSD, Abbvie, Takeda and participated in an advisory board member for MSD, Takeda, Janssen, Pfizer. Pr Paul was speaker for Theradiag and MSD.

**The paper explained**

**Problem**

Secretory immunoglobulin A (SIgA) select gut commensals via glycan–glycan interactions and dynamic transport to and from the mucosa, but SIgA1 and SIgA2 behavior remain largely unknown during Inflammatory Bowel Diseases (IBD).

**Results**

We show that Crohn's disease (CD) IgA1 gains retro‐transport ability and selects a distinct microbiota while Ulcerative colitis (UC) IgA2 loses its natural retro‐transport function and reactivity. Additionally, CD IgAs have increased anti‐glycan reactivity while UC IgA presents significant loss in anti‐glycan reactivity. Finally, local IgA2 selection of the microbiota in active CD differs from that of inactive CD. suggesting IgA2 may have more of a pathogenic tissular role in CD while IgA1 has a pathogenic luminal role.

**Impact**

This is the first study dedicated to the specific focus on the impact of IgA subclasses and structure on their interaction with the microbiota in IBD. It suggests that repertoires of IgA^+^ microbiota could be predictive of active disease in CD and that analysis of IgA‐specific glycosylations could be important biomarkers for IBD diagnosis and outcome prediction. In addition, characterization of IgA^+^−microbiota fraction could be an important predictive parameter for fecal microbiota transplantation efficacy given its diverse outcome in both CD and UC.

## Supporting information




Appendix

## Data Availability

The 16S datasets produced in this study are available in the following databases: [16S rRNA sequences]: [Portail Data INRAE] [https://doi.org/10.57745/BOUNLH]. The datasets produced in this study are available in the following databases: *Glycan‐based mass spectrometry proteomics data*. ProteomeXchange Consortium (identifier: PXD024060) via the PRIDE partner repository (Perez‐Riverol *et al*, 2019): http://proteomecentral.proteomexchange.org/cgi/GetDataset?ID=PXD024060 [Project Name: Alteration of microbiota antibody‐mediated immune selection contributes to dysbiosis in IBD; Project accession: PXD024060].
