# Supplementary material for: Alteration of microbiota antibody‐mediated immune selection contributes to dysbiosis in inflammatory bowel diseases
Source: EMBO Mol Med. 2022 Jul 4;14(8):e15386. doi: 10.15252/emmm.202115386 (PMC9358401; doi:10.15252/emmm.202115386)
Supplement: Supplementary file 1 — Appendix [file EMMM-14-e15386-s001.pdf]

APPENDIX

Table of Content

Appendix Figure S1. Gating strategy for IgA-bound microbiota by flow cytometry. ____________ *2*

Appendix Figure S2. ____________ *3*

Appendix Figure S3. ____________ *4*

Appendix Figure S4. ____________ *5*

Appendix Table S1. Clinical characteristics of subjects in the cohort. ____________ *7*

Appendix Table S2. ____________ *8*

Appendix Table S3. Correlation between percent IgA1 or IgA2 reverse transcytosis and Dectin-1 binding ____________ *8*

Appendix Table S4. Primary sequences of IgA1/IgA2 heavy chain peptides potentially N/O-glycosylated. ____________ *9*

Appendix Table S5. IgA1 heavy chain N-glycopeptides identified in CD samples. ____________ *9*

Appendix Table S6 - IgA2 heavy chain N-glycopeptides identified in CD and UC samples. _____ *10*

Appendix Table S7. IgA1 heavy chain O-glycoforms identified in CD and UC samples. _______ *11*

Appendix Table S8. Glycan motifs recognition by CD IgAs ____________ *12*

Appendix Table S11. Glycan motifs in IgA1-targeted clusters ____________ *16*

Appendix Table S12. Glycan motifs in IgA2-targeted clusters ____________ *18*

Appendix Table S13. Differentially recognised species in the IgA1^{+} fraction of the CD stool microbiota ____________ *19*

Appendix Table S14. FISH probe sequences (Eurogentec) ____________ *19*

Appendix Figures

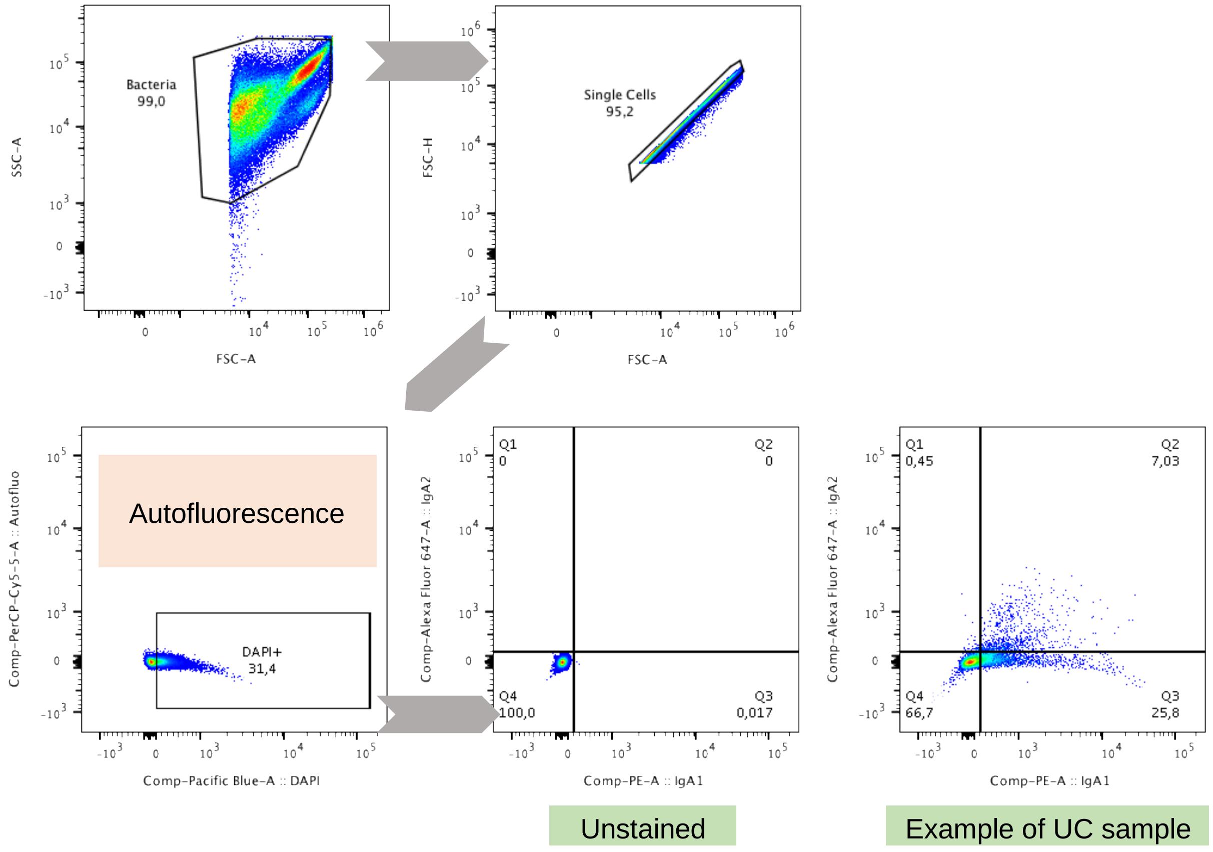

Appendix Figure S1. Gating strategy for IgA-bound microbiota by flow cytometry.

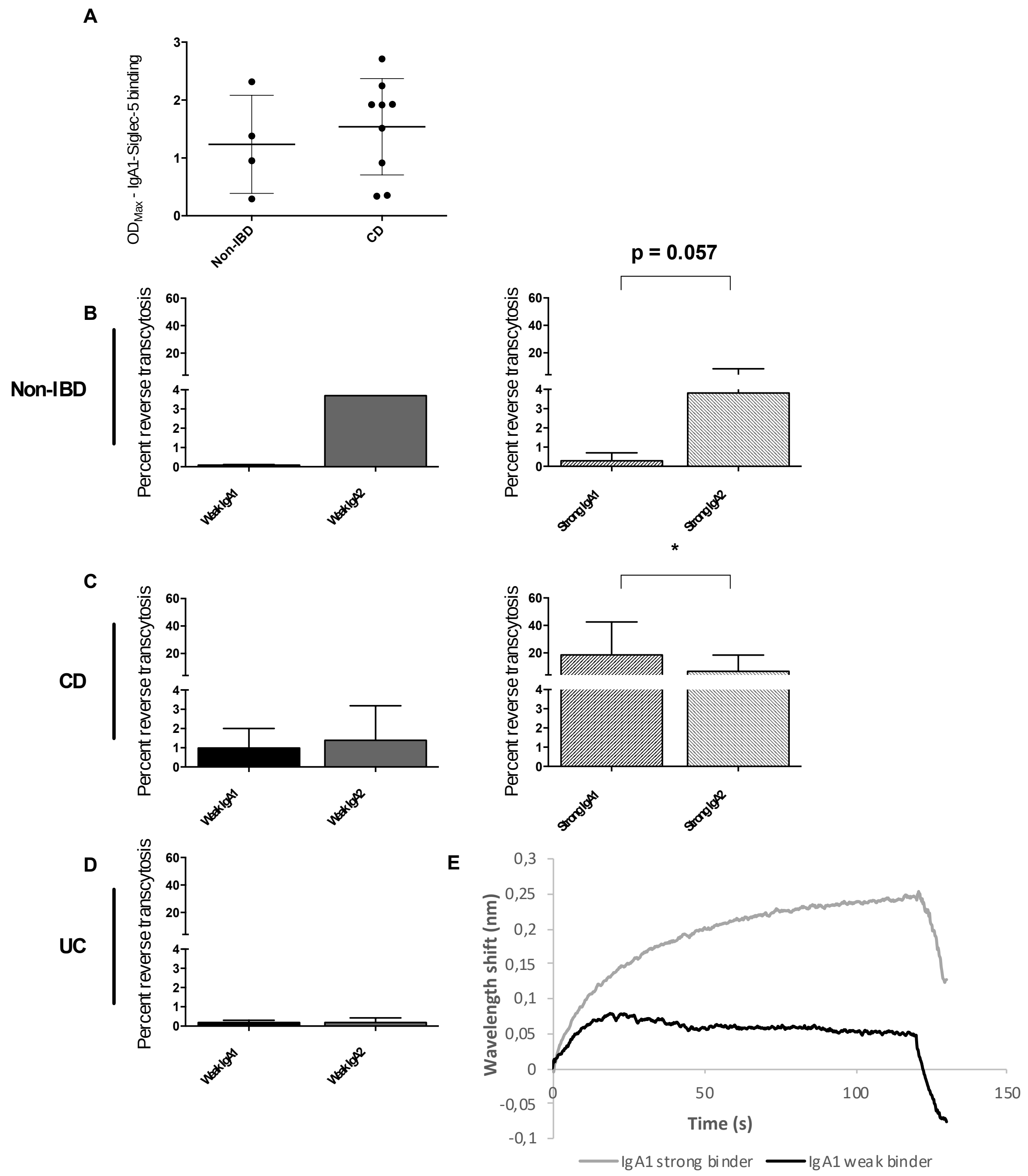

Appendix Figure S2.

(**A**) ELISA assay of IgA1-Siglec-5 binding, at a rate of one receptor per 10 IgA. (**B-D**) ELISA assay of IgA1-Dectin-1 binding, at a rate of one receptor per 10 IgA compared to the percent reverse-tanscytosis on an inverted model of FAE from Caco2 and Raji cells co-culture of purified IgA with either weak or strong Dectin-1 binding in non-IBD patients (**B**), CD (**C**) or UC (**D**) patients. Data were analyzed using the Kruskall-Wallis test when possible, or the Mann-Whitney test. * : p-value < 0,05. Non-IBD : n= 6; CD : n= 8 ; UC : n=5. (**E**) Representative association and dissociation curves of IgA&-Dectin-1 complexes for CD weak (black line) and strong binders (grey line) by biolayer interferometry.

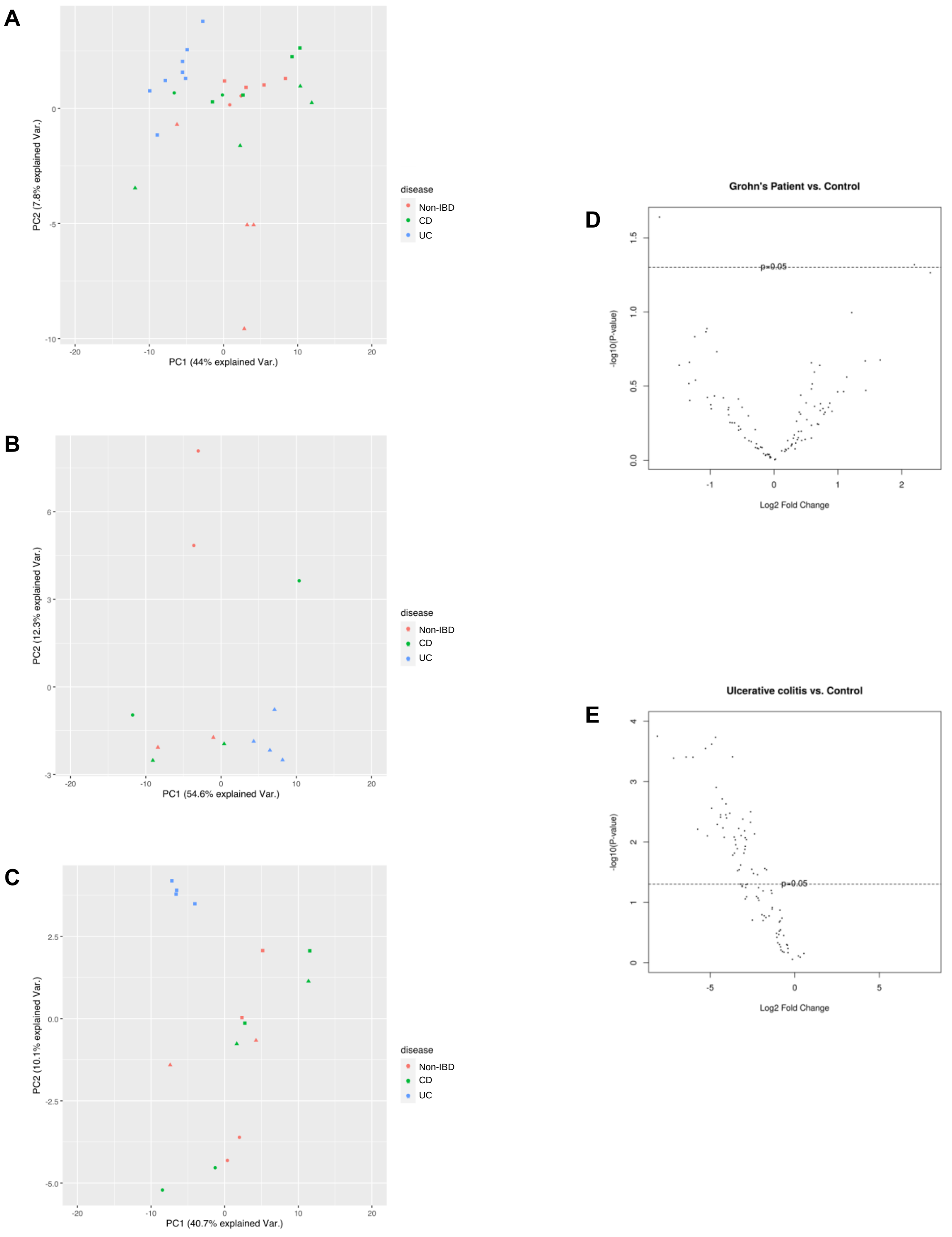

Appendix Figure S3.

Principal component analysis (PCA) plot of 100 glycan targets of purified IgA1 and IgA2 combined (**A**), IgA1 only (**B**) or IgA2 only (**C**) in non-IBD, CD and UC groups. (**D**) Plotted distributions of CD versus non-IBD IgA1 and IgA2 glycan targets after log2 data transformation using non-IBD datasets as controls. (**E**) Plotted distributions of UC versus non-IBD IgA1 and IgA2 glycan targets after log2 data transformation using non-IBD datasets as controls. For IgA1, Non-IBD : n= 4; CD : n= 4; UC : n= 4. For IgA2, Non-IBD : n= 6; CD : n= 6; UC : n= 6. Data were analyzed using the limma package in R and the dotted line indicates significance thresholds.

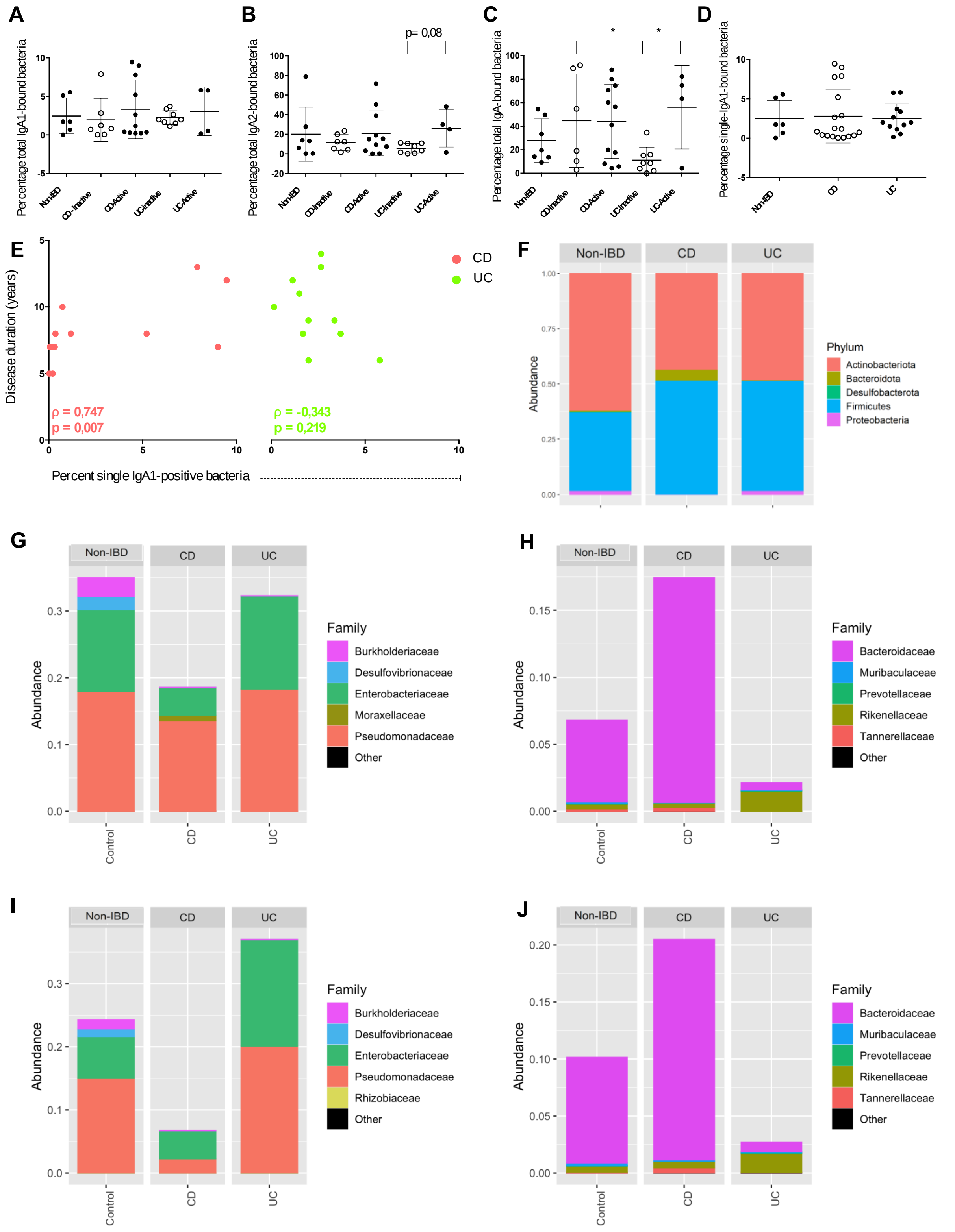

Appendix Figure S4.

(**A-C**) Flow cytometry analysis of IgA-bound bacteria according to disease status at the time of collection; (**A**) Total IgA1^{+} IgA2^{-} bacteria, (**B**) Total IgA1^{-} IgA2^{+} bacteria, (**C**) Total IgA1^{+} IgA2^{+} bacteria. (**D**) Total single-IgA1-positive bacteria frequency. Data were analysed with a Kruskall-Wallis

multiple comparison test. *: p-value < 0,05. (**E**) Plotted data for correlation between disease duration and IgA^{+} $IgA2^{-}$ bacteria counts analyzed by Spearman's correlation test in CD and UC (**F**) Phylum-level composition of fecal microbiota in non-IBD, CD and UC. (**G**) Family-level composition of IgA1-bound *Proteobacteria* in non-IBD, CD and UC. (**H**) Family-level composition of IgA1-bound *Bacteroidetes* in non-IBD, CD and UC. (**I**) Family-level composition of IgA2-bound *Proteobacteria* in non-IBD, CD and UC. (**J**) Family-level composition of IgA2-bound *Bacteroidetes* in non-IBD, CD and UC.

	Non-IBD	CD	UC
**Male/Female** (%)	37 (3) / 63 (5)	47,1 (8) / 52,9 (9)	50 (6) / 50 (6)
**Age** (yr, mean ± SD)	41,4 (±24,3)	43,9 (±18,4)	53,8 (±19,7)
**Age at diagnostic** (yr, mean ± SD)	-	32,5 (±16,9)	48,7 (±15,9)
**Duration** (yr, mean ± SD)	-	8,2 (±3,1)	8,7 (±2,8)
**Phenotype** (%)	-		
CD	-		-
L1		47,1 (8)	
L2		29,4 (5)	
L3		23,5 (4)	
Behavior	-		
B1		76,5 (13)	
B2		17,6 (3)	
B3		5,88 (1)	
UC	-	-	
E1		-	25 (3)
E2		-	25 (3)
E3		-	50 (6)
**Operations (%)**	-	11,8 (2)	0
**Disease status (%)**	-		
Active		70,6 (12)	33,3 (4)
Remission		29,4 (5)	66,7 (8)
**Smoking habits (%)**	-		
Yes		64,7 (11)	unknown
No		35,3 (6)	unknown
**Medication (%)**	-		
Anti-TNF		100 (17)	100 (12)

Appendix Table S1. Clinical characteristics of subjects in the cohort.

Patient from the non-IBD cohort were included following negative result after screening for possible IBD. Disease status was assessed according to endoscopic activity. Endoscopic remission for CD corresponds to an SES-CD score below 4. Endoscopic remission for UC corresponds to a Mayo score of 0 or 1. SD = standard deviation.

	[IgA1]			[IgA2]			[Total IgA]		
	Non-IBD	CD	UC	Non-IBD	CD	UC	Non-IBD	CD	UC
Age	p=0.475	r = 0.006<br>p=0.081	p=0.562	p = 0.778	p = 0.386	p = 0.940	p = 0.475	p = 0.352	p = 0.823
Disease duration	n/a	p=0.739	r = 0,598<br>p=0.073	n/a	p = 0.739	p = 0.551	n/a	p = 0.372	p = 0.192
[IgM]	p=0.103	r = -0.211<br>p=0.010	p=0.116	p = 0.175	r = 0,426<br>p = 0.089	r = 0,<br>p = 0.014	r = -0.714<br>p = 0.033	r = 0.818<br>p = 0.003	r = 0.718<br>p = 0.016
[IgG]	p=0.333	p=0.253	p=0.053	p = 0.417	p = 0.253	p = 0.186	r = -0.999<br>p = 0.083	p = 0.389	r = 0.916<br>p = 0.0002
[SC]	p=0.356	r = -0.074<br>p=0.001	r =0.967<br>p=0.0002	p = 0.497	r = 0.723<br>p = 0.001	r = 0,917<br>p = 0.001	r = -0.371<br>p = 0.058	r = 0.664<br>p = 0.031	r = 0.767<br>p = 0.021
% IgA1+	p=0.658	p=0.410	p=0.635	p=0.419	p = 0.540	p = 0.528	p = 0.595	p = 0.860	p = 0.881
% IgA2+	p=0.175	p=0.906	p=0.104	p=1.000	p = 0.839	p = 0.355	p = 0.302	p = 0.694	p = 0.237
% double +	p=0.497	p=0.362	r = 0.713<br>p=0.005	p=0.658	p = 0.362	p = 0.228	p = 0.302	p = 0.138	p = 0.273

Appendix Table S2.

Correlation results for total total IgA, IgA1 and IgA2 concentration. Brackets indicate ‘concentration’. Age and disease duration are expressed in years. Percentages refer to percentage of bound bacteria by flow cytometry.

Group	Spearman’s ρ	P-value
CTR IgA1 – Dectin-1 binding	0,698	0,506
CD IgA1 – Dectin-1 binding	**0,738**	**0,046**
CTR IgA2 – Dectin-1 binding	0,927	0,244
CD IgA2 – Dectin-1 binding	-0,485	0,180
UC IgA2 – Dectin-1 binding	0,070	0,930

Appendix Table S3. Correlation between percent IgA1 or IgA2 reverse transcytosis and Dectin-1 binding

IgA2 N-glycosylation sites	Position	Peptide sequence	$[M+H]^+$ (Da)
N92	[89-102]	HYTNSSQDVTVPCR	1663.7493
N131	[119-140]	PALEDLLLGSEANLTCTLTGLR	2357.2380
N205	[200-208]	TPLTANITK	958.5567
N327	[319-340]	MAGKPTHVNVSVVMAEVDGTCY	2351.0828

Appendix Table S4. Primary sequences of IgA1/IgA2 heavy chain peptides potentially N/O-glycosylated.

[M+H]+ Monoisotopic m/z are calculated with cysteine carbamidomethylation and free N/S/T amino acids.

Glycan composition[1]				Glycan structure[2]	IgA1 – Site N144				IgA1 - Site N340			
HexNAc	Hex	Fuc	Neu5Ac		$[M+H]^+$ m/z exp[3]	Δm[4] (ppm)	RT[5] (min)	Score[6]	$[M+H]^+$ m/z exp[3]	Δm[4] (ppm)	RT[5] (min)	Score[6]
2	3	0	0	Man3	3855.9242	2.26	74.49	888	3239.4565	-0.86	51.60	560
2	4	0	0	Man4	4017.9672	-0.28	74.37	249	3401.5104	-0.52	51.43	231
2	5	0	0	Man5	4180.0299	2.11	74.14	875	3563.5671	0.61	51.21	562
2	6	0	0	Man6	4342.0763	0.54	73.89	859	3725.6070	-2.88	51.04	290
2	7	0	0	Man7	4504.1310	0.94	73.85	674	3887.6678	-0.71	50.85	325
2	8	0	0	Man8	4666.1916	2.56	73.62	911	4049.7290	1.37	50.73	491
3	3	0	0	Man3B	4059.0001	1.30	74.14	935	3442.5425	1.10	51.40	586
3	4	0	0	Man4B/Hybrid	4221.0539	1.46	74.23	879	3604.5882	-0.90	51.21	319
3	5	0	0	Man5B/Hybrid	4383.1061	1.28	73.99	910	3782.6449	1.50	51.21	197
4	3	0	0	G0	4262.0827	1.98	74.16	769	3645.6174	-0.17	51.22	542
4	3	1	0	G0F					3791.6755	-0.12	51.41	583
5	3	0	0	G0B	4465.1540	0.08	74.06	781	3848.6953	-0.55	51.44	389
5	3	1	0	G0FB					3994.7582	0.71	51.50	452
4	4	0	0	G1	4424.1413	3.21	74.30	639				
4	4	0	1	G1S1	4715.2287	1.31	80.23	397	4098.7658	-0.12	60.75	191
4	4	1	0	G1F					3969.7207	-0.76	51.11	230
5	4	0	0	G1B					4010.7502	0.52	51.08	430
4	5	0	0	G2	4586.1925	2.76	74.33	537				

Appendix Table S5. IgA1 heavy chain N-glycopeptides identified in CD samples.

N-linked glycans are localized on the residues N144 and N340. [1]The glycan composition is provided by Byonic software. [2] Hypothetical glycan structures referenced using IgG glycans naming system. [3] N-glycans are located on the IgA1 tryptic peptides [127-153] (LSLHRPALEDLLLGSEANLTCTLTGLR) and [332-353] (LAGKPTHVNVSVVMAEVDGTCY) respectively for N144 and N340. Experimental $[M+H]^+$ ions are referenced in the Table; 2+, 3+ and 4+ precursor ions were observed in our spectra. [4] Dm is measurement error. [5] RT is retention time. [6] Score is given by Byonic software.

A

Glycan composition[1]				Glycan structure[2]	CD IgA2 N131				UC IgA2 N131			
HexNAc	Hex	Fuc	Neu5Ac		$[M+H]^+$ m/z exp[3]	Δm[4] (ppm)	RT[5] (min)	Score[6]	$[M+H]^+$ m/z exp[3]	Δm[4] (ppm)	RT[5] (min)	Score[6]
2	3	0	0	Man3	3249.5617	1.98	72.82	470				
2	5	0	0	Man5	3573.6628	0.52	72.13	535	3573.6606	-0.09	87.35	298
2	6	0	0	Man6	3735.7108	-0.80	71.93	362	3735.7258	3.22	86.97	200
2	8	0	0	Man8					4059.8188	-0.15	86.46	281
2	9	0	0	Man9					4221.8727	0.11	86.37	301
4	3	0	0	G0	3655.7241	2.75	72.33	267	3655.7157	0.44	87.44	281
5	3	0	0	G0B	3858.8034	2.60	72.23	333	3858.7961	0.70	87.42	665

B

Glycan composition[1]				Glycan structure[2]	CD IgA2 N205				UC IgA2 N205			
HexNAc	Hex	Fuc	Neu5Ac		$[M+H]^+$ m/z exp[3]	Δm[4] (ppm)	RT[5] (min)	Score[6]	$[M+H]^+$ m/z exp[3]	Δm[4] (ppm)	RT[5] (min)	Score[6]
2	3	0	0	Man3	1850.8781	2.22	31.20	682				
4	3	0	0	G0	2257.0333	0.23	25.12	351				
4	3	1	0	G0F	2403.0919	0.52	24.24	464	2403.0965	2.43	30.70	277
5	3	0	0	G0B	2460.1102	-0.77	25.32	62	2460.1133	0.49	30.90	290
5	3	1	0	G0FB	2606.1714	0.54	25.31	374	2606.1703	0.12	30.98	10
4	4	1	0	G1F	2565.1498	2.45	25.57	337				
5	4	1	0	G1FB	2768.2221	-0.25	24.83	138	2768.2238	0.34	30.39	120
5	4	1	1	G1S1FB					3059.3197	0.48	36.89	134
5	5	1	0	G2FB	2930.2751	-0.21	25.18	20				

Appendix Table S6 - IgA2 heavy chain N-glycopeptides identified in CD and UC samples.

(**A)** N-linked glycans localized on the N131 residues. (**B)** N-linked glycans localized on the N205 residues. [1]The glycan composition is provided by Byonic software. [2] Hypothetical glycan structures referenced using IgG glycans naming system. [3] N-glycans are located on the IgA2 tryptic peptides [119-140] (PALEDLLLGSEANLTCTLTGLR) for Table A and [200-208] (TPLTANITK) for Table B. Experimental $[M+H]^+$ ions are referenced in the Table; 2+, 3+ and 4+ precursor ions were observed in our spectra. [4] Dm is measurement error. [5] RT is retention time. MS acquisitions started before/after nanoLC preconcentration step respectively for UC/CD. [6] Score is given by Byonic software.

Glycan composition[1]			CD O-glycoforms				UC O-glycoforms			
GalNAc	Gal	Neu5Ac	$[M+H]^+$ m/z exp[2]	Δm[3] (ppm)	RT[4] (min)	Score[5]	$[M+H]^+$ m/z exp[2]	Δm[3] (ppm)	RT[4] (min)	Score[5]
1	0	0	4339.9767	1.71	41.78	1386	4339.9718	0.59	46.16	1065
1	1	0	4502.0226	0.11	41.32	1218	4502.0285	1.41	45.61	563
1	1	1	4793.1154	-0.45	44.70	1176	#	#	#	#
2	0	0	4543.0475	-0.25	40.89	1109	4543.0412	-1.65	45.61	320
2	1	0	4705.1017	0.05	40.53	1184	#	#	#	#
2	1	1	4996.1984	0.30	43.81	1162	4996.1901	-1.36	48.40	101
2	2	0	4867.1476	-1.38	40.29	946	#	#	#	#
2	2	1	5158.2531	0.65	43.28	1067	#	#	#	#
2	2	2	5449.3546	1.74	46.49	540	#	#	#	#
3	0	0	4746.1265	-0.31	41.97	1175	#	#	#	#
3	1	0	4908.1823	0.29	41.14	1216	#	#	#	#
3	2	0	5070.2389	1.03	40.71	1138	5070.2316	-0.41	43.63	134
3	2	1	5361.3225	-1.24	42.66	177	5361.3332	0.76	46.95	156
3	3	0	5232.2858	-0.14	39.69	941	5232.2824	-0.79	42.90	273
3	3	1	5523.3708	-2.01	42.55	876	5523.3825	0.10	46.04	197
3	3	2					5814.4586	-3.21	49.79	135
3	3	3					6105.5778	0.83	53.91	37
4	0	0	4949.2023	-1.03	40.07	986	#	#	#	#
4	1	0	5111.2746	2.80	40.45	1162	#	#	#	#
4	1	1	5402.3532	-0.46	42.99	720	#	#	#	#
4	2	0	5273.3140	0.19	39.50	1154	5273.3200	1.31	43.72	278
4	2	1	5564.4005	-1.43	42.38	902	5564.4020	-1.16	46.89	165
4	3	0	5435.3629	-0.54	39.15	617	5435.3625	-0.63	43.23	437
4	3	1	5726.4567	-0.80	41.83	808	5726.4611	-0.04	45.95	402
4	3	2	6017.5651	1.40	44.56	848	6017.5587	0.34	49.31	325
4	3	3					6308.6569	0.75	53.12	96
4	4	0	5597.4167	-0.37	38.34	808	5597.4210	0.42	42.31	638
4	4	1	5888.5236	1.61	41.18	857	5888.5063	-1.33	45.16	640
4	4	2	6179.6116	0.34	48.82	460	6179.6165	1.13	48.38	557
4	4	3					6470.7060	0.16	52.60	492
5	1	0	5314.3361	-0.66	39.57	927				
5	2	0	5476.3898	-0.48	38.80	629	5476.4030	1.93	42.91	91
5	2	1	5767.4907	0.49	41.61	949				
5	3	0	5638.4593	2.49	38.06	915	5638.4442	-0.19	42.06	197
5	3	1	5929.5377	-0.49	40.75	656	5929.5299	-1.81	45.13	225
5	3	2	6220.6334	-0.44	43.56	724	6220.6425	1.04	48.42	522
5	3	3					6511.7381	1.02	52.43	89
5	4	0	5800.5137	2.70	37.51	784	5800.5070	1.54	41.36	342
5	4	1	6091.5837	-1.61	40.04	839	6091.6032	1.60	43.97	310
5	4	2	6382.6835	-0.84	43.05	705	6382.7006	1.84	47.25	442
5	4	3	6673.7879	0.54	46.10	251	6673.7877	0.50	51.13	347
5	5	0	5962.5548	0.65	41.02	568	5962.5499	-0.17	41.05	256
5	5	1	6253.6473	0.16	43.92	497	6253.6455	-0.14	43.79	283
5	5	2					6544.7423	0.08	47.19	353
5	5	3					6835.8398	0.50	51.21	303
6	2	0	5679.4601	-2.06	38.51	660				
6	3	0	5841.5197	-0.85	37.78	301	5841.5138	-1.85	41.68	156
6	3	1	6132.6032	-2.75	40.14	21	6132.6090	-1.79	44.33	27
6	3	2					6423.7150	-0.07	47.90	48
6	3	3					6714.7996	-1.67	51.35	167
6	4	0	6003.5851	1.28	36.71	349	6003.5822	0.79	40.92	34
6	4	1					6294.6562	-2.65	43.98	246
6	4	2					6585.7829	2.22	46.85	301
6	4	3					6876.8606	2.41	51.51	259
6	5	2					6747.8392	2.68	46.63	131
6	5	3					7038.9058	2.79	51.72	129

Appendix Table S7. IgA1 heavy chain O-glycoforms identified in CD and UC samples.

[1]The glycan composition is provided by Byonic. [2] N-glycans are located on the IgA1 peptide [89-126] HYTNPSQDVTVPCPVPSTPPTPSPSTPPTPSPSCCHPR. Experimental $[M+H]^+$ ions are referenced in the Table; mainly 3+ and 4+ precursor ions were observed in our spectra. [3] Dm is measurement error. [4] RT is retention time. MS acquisitions started before/after nanoLC preconcentration step respectively for UC/CD. [4] Score is given by Byonic software. # m/z range showing contamination by other multiply charges species covering the glycoforms signals.

Ig	Motif	LogFC	p-value
**All IgA**	Neu5Ac-2,3-Gal-1,4-Glc-Sp1	**-1,80**	**0.019**
	SGP	**2,12**	**0.049**
	Neu5Ac-2,3-Gal-1,4-(Fuc-1,3)-Glc-[3-Sialyl-3-fucosyllactose/F-SL]-Sp1	2,45	0.050
	Sisomicin Sulfate	1,22	0.096
**IgA1**	Gal-1,4-Gal-1,4-Glc-Sp	-2,92	0.080
	4-P-GlcNac-1,4-Man-Sp	-3,57	0.085
	Gal-1,3-(Neu5Ac-2,6)-GalNac-Sp	2,01	0.086
**IgA2**	Neu5Ac-2,3-Gal-1,4-(Fuc-1,3)-Glc-[3-Sialyl-3-fucosyllactose/F-SL]-Sp1	**2,68**	**0.037**
	Maltotetraose-Sp1	**2,74**	**0.044**
	Neu5Ac-2,3-Gal-1,4-Glc-Sp1	**-1,71**	**0.045**
	Gal-1,3-GalNac-1,4-(Neu5Ac-2,3)-Gal-1,4-Glc-Sp1	2,96	0.064
	GalNAc-1,3-Gal-1,4-Gal-1,4-Glc-Sp	2,06	0.068

Appendix Table S8. Glycan motifs recognition by CD IgAs

ID	logFC	AveExpr	t	P.Value	adj.P.Val
Fuc- -1,2-Gal- -1,4-Glc- -[Blood H antigen trisaccharide]-Sp1	-6.590417	4.744172	-4.949344	0.0002511	0.0249564
Gal- -1,3-GalNAc- -Sp1	-6.681671	3.355220	-4.571872	0.0004991	0.0249564
Neu5Gc- -2,3-Gal- -1,3-(Fuc- -1,4)-GlcNAc- -[Sialyl Lewis A]-Sp	-6.567199	4.202897	-4.178340	0.0010400	0.0334627
GalNAc- -1,4-GlcNAc- -Sp2	-7.467455	6.047595	-4.045053	0.0013385	0.0334627
Gal- -1,3-(Fuc- -1,4)-GlcNAc- -1,3-Gal -1,4-(Fuc- -1,4)-Glc- -[Lewis A]-Sp1	-6.552701	3.830655	-3.755552	0.0023276	0.0465520
Maltohexaose- -Sp1	-7.377784	5.335618	-3.494442	0.0038523	0.0496073
Maltoheptaose- -Sp1	-7.061841	7.419834	-3.478684	0.0039716	0.0496073
(Fuc- -1,2)-Gal- -1,4-(Fuc- -1,3)-GlcNAc- -[Lewis Y]-Sp1	-6.175025	2.763122	-3.408702	0.0045484	0.0496073
Neu5Ac- -2,8-Neu5Ac- -2,8-Neu5Ac- -2,3-Gal- -1,4-Glc- -Sp3	-6.548126	4.466696	-3.387310	0.0047410	0.0496073
Fuc- -1,2-Gal- -1,3-GlcNAc- -1,3-Gal- -1,4-Glc- -[LNFP I]-Sp1	-6.452538	3.393452	-3.363955	0.0049607	0.0496073
GlcNAc- -1,6-(Gal- -1,3)-GalNAc- -OSer-Sp4	-6.935546	5.091995	-3.227770	0.0064617	0.0565658
GalNAc- -1,3-(Fuc- -1,2)-Gal- -1,4-Glc -[Blood A antigen tetrose]-Sp1	-7.697148	4.492655	-3.202404	0.0067879	0.0565658
Neu5Ac- -2,3-Gal- -1,4-Glc- -Sp	-4.779441	6.314388	-2.907011	0.0120374	0.0925955
-1,4-Xylotetrose-Sp1	-5.341835	5.604743	-2.770825	0.0156575	0.1014439
KDN- -2,8-Neu5Ac- -2,3-Gal- -1,4-Glc -Sp	-5.709778	2.882824	-2.748008	0.0163606	0.1014439
Gal- -1,3-GalNAc- -Sp	-5.216042	6.845270	-2.721336	0.0172218	0.1014439
Maltotetraose- -Sp1	-6.441777	3.893764	-2.672397	0.0189184	0.1014439
Gal- -1,4-Gal- -1,4-GlcNAc- -Sp1	-6.479006	5.655039	-2.666874	0.0191198	0.1014439
Neu5Ac- -2,3-Gal- -1,4-(Fuc- -1,3)-GlcNAc- -[Sialyl Lewis X]-Sp	-5.118366	4.747289	-2.654310	0.0195857	0.1014439
Glc- -1,2-Gal- -1,3-Glc- -Sp	-6.556003	4.283860	-2.635895	0.0202888	0.1014439
Acarbose- -Sp1	-5.097598	4.739067	-2.582288	0.0224775	0.1070359
Neu5Ac- -2,6-Gal- -1,3-GlcNAc- -Sp	-4.559677	5.611334	-2.547821	0.0240039	0.1091084
Tobramycin	-3.245836	7.071676	-2.492330	0.0266736	0.1113384
GlcNAc- -1,2-Man- -Sp	-6.276621	3.361695	-2.475860	0.0275196	0.1113384
(Fuc- -1,2)-Gal- -1,3-(Fuc- -1,4)-GlcNAc- -[Lewis B]-Sp1	-4.699540	5.078640	-2.469850	0.0278346	0.1113384
Neu5Ac- -2,6-Gal- -1,3-GalNAc- -Sp	-5.780578	3.955847	-2.419030	0.0306407	0.1140370
Maltotriose- -Sp1	-5.818373	5.082151	-2.416453	0.0307900	0.1140370
Gal- -1,4-(6S)GlcNAc- -Sp	-4.571710	5.413838	-2.371448	0.0335119	0.1179979
Gal- -1,4-Gal- -1,4-Glc- -Sp	-5.064456	5.439550	-2.360321	0.0342194	0.1179979
GlcA- -1,4-GlcNAc- -1,4-GlcA- -Sp	-5.819592	5.396282	-2.301035	0.0382351	0.1196513
Neu5Ac- -2,3-Gal- -1,3-(Fuc- -1,4)-GlcNAc- -[Sialyl Lewis A]-Sp	-4.010125	6.211924	-2.301022	0.0382360	0.1196513
Neu5Ac- -2,8-Neu5Ac- -2,3-Gal -1,4-Glc- -Sp	-4.406680	4.975613	-2.300288	0.0382884	0.1196513
Gal- -1,4-GlcNAc- -1,3-Gal- -1,4-Glc- -[LNnT]-Sp	-4.669214	5.184491	-2.193256	0.0467021	0.1415214
Gal- -1,3-(Fuc- -1,2)-Gal- -1,4-Glc- -[Blood B antigen tetrasaccharide]-Sp1	-4.634118	3.734973	-2.152613	0.0503300	0.1480293
GalNAc- -1,3-(Fuc- -1,2)-Gal- -[Blood A antigen trisaccharide]-Sp1	-4.786949	3.108536	-2.054911	0.0601539	0.1718683
GlcNAc- -1,4-GlcA- -1,4-GlcNAc- 1,4-GlcA- -Sp	-3.964664	6.740448	-1.995736	0.0669376	0.1838174
Neu5Ac- -2,3Gal- -1,4-(6S)GlcNAc- -Sp	-4.433621	4.947328	-1.981944	0.0686166	0.1838174
Neu5Gc- -2,3-Gal- -1,4-Glc- -Sp	-3.900122	5.942836	-1.972003	0.0698506	0.1838174
GalNAc- -1,4-(Neu5Ac- -2,3)-Gal- -1,4-Glc- -[GM2]-Sp	-3.567133	6.498104	-1.893280	0.0803616	0.2060553
GlcNAc- -1,3-(Glc- -1,2-Glc- -1,2)-Gal -1,3-Glc- -Sp	-5.720180	10.592203	-1.869301	0.0838373	0.2095933
Neu5Ac- -2,6-Gal- -1,3-(Neu5Ac- -2,6)-GalNAc- -Sp	-3.390867	7.414372	-1.837297	0.0886875	0.2163110
-Man-Sp	-3.365275	7.772559	-1.794510	0.0955652	0.2233625
Neu5Gc- -2,3-Gal- -1,3-GlcNAc- -Sp	-3.987989	4.953645	-1.791624	0.0960459	0.2233625
Gal- -1,3-GalNAc- -1,4-(Neu5Gc- -2,3)-Gal- -1,4-Glc- -Sp1	-3.683544	4.782399	-1.772913	0.0992146	0.2238621

Appendix Table S9. Glycan motif recognition by UC IgA1.

ID	logFC	AveExpr	t	P.Value	adj.P.Val
Maltoheptaose- -Sp1	-10.177366	9.623456	-4.822570	0.0002347	0.0185735
Neu5Ac- -2,6-Gal- -1,4-Glc- -Sp	-5.004681	4.953134	-4.533753	0.0004127	0.0185735
KDN- -2,8-Neu5Ac- -2,3-Gal- -1,4-Glc -Sp	-6.544193	4.365996	-4.305224	0.0006490	0.0185735
-Glc-Sp	-7.045675	8.327813	-4.237485	0.0007429	0.0185735
GlcNAc- -1,6-(Gal- -1,3)-GalNAc- -OSer-Sp4	-5.984347	6.130118	-3.891166	0.0014907	0.0280501
Neu5Ac- -2,3-Gal- -1,4-(Fuc- -1,3)-GlcNAc- -[Sialyl Lewis X]-Sp	-4.362897	4.964691	-3.831200	0.0016830	0.0280501
GalNAc- -1,4-GlcNAc- -Sp2	-6.211433	9.111515	-3.682706	0.0022747	0.0324951
Fuc- -1,2-Gal- -1,4-GlcNAc- -[Blood H antigen trisaccharide]-Sp1	-5.359058	5.406370	-3.512394	0.0032163	0.0343368
GalNAc- -1,3-(Fuc- -1,2)-Gal- -1,4-Glc -[Blood A antigen tetrose]-Sp1	-8.904305	6.902106	-3.409500	0.0039659	0.0343368
Gal- -1,4-Gal- -1,4-Glc- -Sp	-4.605782	5.176067	-3.405713	0.0039966	0.0343368
Neu5Gc- -2,3-Gal- -1,3-GlcNAc- -Sp	-4.255889	5.410857	-3.386049	0.0041598	0.0343368
GlcNAc- -1,4-GlcNAc- -Sp1	-7.342213	4.266910	-3.380756	0.0042049	0.0343368
Glc- -1,2-Gal- -1,3-Glc- -Sp	-5.520974	4.826140	-3.333903	0.0046258	0.0343368
Gal- -1,4-Gal- -1,4-GlcNAc- -Sp1	-6.555633	6.403433	-3.283301	0.0051276	0.0343368
Gal- -1,4-(Fuc- -1,3)-GlcNAc- -1,3-Gal -Sp1	-4.027449	3.699707	-3.281106	0.0051505	0.0343368
Gal- -1,4-Gal- -1,3-GlcNAc- -Sp	-4.092856	8.086921	-3.184532	0.0062682	0.0391764
-GalNAc-Sp	-4.441128	7.319521	-2.872723	0.0117816	0.0693033
Chitin-trisaccharide-Sp1	-3.968479	5.821790	-2.832935	0.0127628	0.0709044
Maltohexaose- -Sp1	-7.286832	6.954482	-2.663873	0.0178951	0.0941846
Neu5Ac- -2,8-Neu5Ac- -2,8-Neu5Ac- -2,3-Gal- -1,4-Glc- -Sp3	-4.289376	5.679682	-2.590908	0.0206819	0.1012286
Gal- -1,3-Gal- -1,4-Glc- -Sp	-3.402094	6.056665	-2.574005	0.0213846	0.1012286
GlcNAc- -1,3-(Glc- -1,2-Glc- -1,2)-Gal -1,3-Glc- -Sp	-5.978445	6.826053	-2.553446	0.0222703	0.1012286
Neu5Ac- -2,3-Gal- -1,4-Glc- -Sp	-2.617600	5.469975	-2.529765	0.0233341	0.1014525
Neu5Ac- -2,6-Gal- -1,3-(Neu5Ac- -2,6)-GalNAc- -Sp	-2.659482	8.521179	-2.421496	0.0288470	0.1201958
Neomycin trisulfate	-2.334842	8.730945	-2.345848	0.0334105	0.1287887
-Fuc-Sp	-4.193148	6.204700	-2.344695	0.0334851	0.1287887
Kanamycin sulfate	-2.194632	8.217499	-2.300333	0.0364761	0.1350967
GalNAc- -1,3-(Fuc- -1,2)-Gal- -[Blood A antigen trisaccharide]-Sp1	-3.545229	3.367845	-2.247054	0.0403999	0.1442854
4-P-GlcNAc- -1,4-Man- -Sp	-3.869121	6.887956	-2.219258	0.0426005	0.1468984
GlcNAc- -1,2-Man- -Sp	-3.714347	5.194681	-2.173203	0.0464942	0.1549807
Gal- -1,3-Gal- -1,3-GlcNAc- -Sp	-2.149561	7.971651	-2.137848	0.0497047	0.1592826
Gal- -1,4-(6S)GlcNAc- -Sp	-3.731318	5.822946	-2.117029	0.0516899	0.1592826
GalNAc- -1,4-(Neu5Ac- -2,3)-Gal- -1,4-Glc- -[GM2]-Sp	-2.247424	6.458179	-2.108104	0.0525632	0.1592826
-GlcNAc-Sp	-3.022555	7.461608	-2.091166	0.0542581	0.1595825
GlcNAc- -1,4-GlcA- -1,4-GlcNAc- 1,4-GlcA- -Sp	-4.374304	5.347898	-2.033823	0.0603758	0.1708416
Neu5Gc- -2,3-Gal- -1,3-(Fuc- -1,4)-GlcNAc- -[Sialyl Lewis A]-Sp	-3.246485	5.175709	-2.023842	0.0615030	0.1708416
Maltotetraose- -Sp1	-3.178338	5.378883	-1.930619	0.0729950	0.1972838
Gal- -1,3-GalNAc- -1,4-(Neu5Gc- -2,3)-Gal- -1,4-Glc- -Sp1	-3.689783	5.236531	-1.830513	0.0874624	0.2286985
Neu5Ac- -2,3Gal- -1,4-(6S)GlcNAc- -Sp	-1.706805	6.931192	-1.819553	0.0891924	0.2286985
Glc- -1,6-Glc- -1,6-Glc- -Sp1	-3.052352	5.422809	-1.774218	0.0966757	0.2416892

Appendix Table S10. Glycan motif recognition by UC IgA2.

Ord	Biomarker	Ord	Biomarker	Ord	Biomarker	Ord	Biomarker	Ord	Biomarker
Cluster1									
1	Gal- -1,3-GalNAc- -1,4-(Neu5Ac- -2,3)-Gal- -1,4-Glc- -Sp1	4	Gal- -1,3-(Neu5Ac- -2,6)-GalNAc- -Sp	7	SGP	10	Neu5Ac- -2,6-(Neu5Ac- -2,3)-Gal- -1,3-GalNAc- -Sp	13	Gal- -1,4-(Fuc- -1,3)-Glc- -Sp
2	-Gal-Sp	5	Neu5Ac- -2,8-Neu5Ac- -2,6-Gal- -1,4-Glc-Sp5	8	-Fuc-Sp	11	Gal- -1,3-Gal- -Sp1	14	Sisomicin Sulfate
3	GalNAc- -1,3-Gal- -1,4-Gal- -1,4-Glc- -Sp	6	Neu5Ac- -2,3-Gal- -1,4-(Fuc- -1,3)-Glc -[3-Sialyl-3-fucosyllactose/F-SL]-Sp1	9	Neu5Ac- -2,6-Gal- -1,4-Glc- -Sp	12	Neu5Ac- -2,3-Gal- -1,3-(Neu5Ac- -2,6)-GalNAc- -Sp		
Cluster2									
1	Gal- -1,4-(Fuc- -1,3)-GlcNAc- -1,3-Gal -Sp1	6	Neu5Ac- -2,6-Gal- -1,3-GalNAc- -Sp	11	Kanamycin sulfate	16	D-pentamannuronic acid- -Sp1	21	Gal- -1,4-GlcNAc- -1,3-Gal- -1,4-Glc- -[LNnT]-Sp
2	Neu5Gc- -2,6-Gal- -1,3-GlcNAc- -Sp	7	GlcNAc- -1,2-Man- -Sp	12	Neomycin trisulfate	17	Gentamicin Sulfate	22	GlcNAc- -1,6-(Gal- -1,3)-GalNAc- -OSer-Sp4
3	Geneticin Disulfate Salt (G418)	8	(Fuc- -1,2)-Gal- -1,4-(Fuc- -1,3)-GlcNAc- -[Lewis Y]-Sp1	13	Gal- -1,3-Gal- -1,3-GlcNAc- -Sp	18	-Man-Sp	23	Maltotriose- -Sp1
4	Gal- -1,4-GlcNAc- -Sp	9	KDN- -2,8-Neu5Ac- -2,3-Gal- -1,4-Glc -Sp	14	GlcNAc- -1,4-GlcNAc- -Sp1	19	Glc- -1,6-Glc- -1,4-Glc- -Sp1		
5	Gal- -1,3-GalNAc- -1,3-Gal- -Sp1	10	Gal- -1,3-GlcNAc- -Sp	15	GalNAc- -1,4-(Neu5Ac- -2,3)-Gal- -1,4-Glc- -[GM2]-Sp	20	Glc- -1,6-Glc- -1,6-Glc- -Sp1		
Cluster3									
1	(Fuc- -1,2)-Gal- -1,3-(Fuc- -1,4)-GlcNAc- -[Lewis B]-Sp1	6	-GlcNAc-Sp	11	Gal- -1,3-GalNAc- -1,4-(Neu5Gc- -2,3)-Gal- -1,4-Glc- -Sp1	16	Gal- -1,3-(Fuc- -1,4)-GlcNAc- -[Lewis A]-Sp	21	Gal- -1,3-GalNAc- -Sp1
2	Acarbose- -Sp1	7	Gal- -1,4-Glc- -Sp	12	GalNAc- -1,4-(Neu5Ac- -2,8-Neu5Ac- -2,3)-Gal- -1,4-Glc- -[GD2]-Sp	17	Fuc- -1,2-Gal- -1,3-GlcNAc- -1,3-Gal- -1,4-Glc- -[LNFP I]-Sp1		
3	Fuc- -1,2-Gal- -1,4-GlcNAc- -[Blood H antigen trisaccharide]-Sp1	8	Neu5Ac- -2,3-Gal- -1,4-Glc- -Sp	13	Gal- -1,4-(6S)GlcNAc- -Sp	18	Gal- -1,3-(Fuc- -1,4)-GlcNAc- -1,3-Gal -1,4-(Fuc- -1,4)-Glc- -[Lewis A]-Sp1		
4	Neu5Ac- -2,3-Gal- -1,3-(Fuc- -1,4)-GlcNAc- -[Sialyl Lewis A]-Sp	9	Neu5Gc- -2,8-Neu5Ac- -2,3-Gal- -1,4-Glc- -Sp	14	Neu5Ac- -2,8-Neu5Ac- -2,8-Neu5Ac- -2,3-Gal- -1,4-Glc- -Sp3	19	Neu5Gc- -2,3-Gal- -1,3-(Fuc- -1,4)-GlcNAc- -[Sialyl Lewis A]-Sp		

Ord	Biomarker	Ord	Biomarker	Ord	Biomarker	Ord	Biomarker	Ord	Biomarker
5	Gal- -1,3-(Fuc- -1,2)-Gal- -[Blood B antigen trisaccharide]-Sp1	10	Neu5Ac- -2,6-Gal- -1,3-GlcNAc- -Sp	15	Neu5Gc- -2,3-Gal- -1,3-GlcNAc- -Sp	20	Fuc- -1,2-Gal- -1,4-Glc- -[Blood H antigen trisaccharide]-Sp1		
Cluster4									
1	Chitin-trisaccharide-Sp1	3	Neu5Ac- -2,8-Neu5Gc- -2,3-Gal- -1,4-Glc- -Sp	5	Neu5Ac- -2,3-Gal- -1,3-GlcNAc- -Sp	7	GalNAc- -1,3-(Fuc- -1,2)-Gal- -[Blood A antigen trisaccharide]-Sp1	9	Neu5Ac- -2,3-Gal- -1,4-(Fuc- -1,3)-GlcNAc- -[Sialyl Lewis X]-Sp
2	Gal- -1,4-(Fuc- -1,3)-GlcNAc- -[LewisX]–Sp	4	GlcNAc- -1,6-GlcNAc- -Sp	6	Neu5Gc- -2,3-Gal- -1,4-Glc- -Sp	8	L-pentaguluronic acid- -Sp1	10	Gal- -1,3-(Fuc- -1,2)-Gal- -1,4-Glc- -[Blood B antigen tetrasaccharide]-Sp1
Cluster5									
1	Neu5Ac- -2,3Gal- -1,4-(6S)GlcNAc- -Sp	8	-GalNAc-Sp	15	Neu5Ac- -2,6-Gal- -1,3-(Neu5Ac- -2,6)-GalNAc- -Sp	22	-1,4-Xylotetrose-Sp1	29	-D-Rha-Sp
2	Neu5Ac- -2,8-Neu5Ac- -2,3-Gal -1,4-Glc- -Sp	9	Glc- -1,4-Glc- -Sp1	16	Gal- -1,3-GalNAc- -Sp	23	Gal- -1,3-Gal- -1,4-Glc- -Sp	30	Glc- -1,2-Gal- -1,3-Glc- -Sp
3	GalNAc- -1,3-Gal- -1,4-Glc- -Sp	10	Gal- -1,4-Gal- -1,4-Glc- -Sp	17	GlcNAc- -1,4-GlcA- -1,4-GlcNAc- 1,4-GlcA- -Sp	24	-Rha-Sp	31	Gal- -1,4-Gal- -1,4-GlcNAc- -Sp1
4	GlcNAc- -1,3-Gal- -1,4-Glc- -Sp	11	GlcA- -1,4-GlcNAc- -1,4-GlcA- -Sp	18	Tobramycin	25	Gal- -1,4-Gal- -1,3-GlcNAc- -Sp	32	Maltohexaose- -Sp1
5	Neu5Ac- -2,6-Gal- -1,4-Glc- -Sp	12	4-P-GlcNAc- -1,4-Man- -Sp	19	GlcNAc- -1,3-(Glc- -1,2-Glc- -1,2)-Gal -1,3-Glc- -Sp	26	-Glc-Sp		
6	Neu5Ac- -2,3-Gal- -Sp1	13	GalNAc- -1,3-(Fuc- -1,2)-Gal- -1,4-Glc -[Blood A antigen tetrose]-Sp1	20	GalNAc- -1,4-GlcNAc- -Sp2	27	D-cellose- -Sp1		
7	Glc- -1,2-Gal- -Sp	14	Maltotetraose- -Sp1	21	Maltoheptaose- -Sp1	28	-D-GlcA-Sp		

Appendix Table S11. Glycan motifs in IgA1-targeted clusters

Ord	Biomarker	Ord	Biomarker	Ord	Biomarker	Ord	Biomarker	Ord	Biomarker
Cluster1									
1	Gal- -1,4-(Fuc- -1,3)-GlcNAc- -[LewisX]–Sp	4	Neu5Ac- -2,6-Gal- -1,4-Glc- -Sp	7	SGP	10	Gal- -1,3-(Neu5Ac- -2,6)-GalNAc- -Sp		
2	Neu5Ac- -2,3-Gal- -1,3-(Fuc- -1,4)-GlcNAc- -[Sialyl Lewis A]-Sp	5	Gal- -1,4-(Fuc- -1,3)-Glc- -Sp	8	Geneticin Disulfate Salt (G418)	11	D-pentamannuronic acid- -Sp1		
3	GalNAc- -1,3-Gal- -1,4-Gal- -1,4-Glc- -Sp	6	Neu5Ac- -2,3-Gal- -Sp1	9	Acarbose- -Sp1				
Cluster2									
1	(Fuc- -1,2)-Gal- -1,4-(Fuc- -1,3)-GlcNAc- -[Lewis Y]-Sp1	4	GlcNAc- -1,2-Man- -Sp	7	KDN- -2,8-Neu5Ac- -2,3-Gal- -1,4-Glc -Sp	10	(Fuc- -1,2)-Gal- -1,3-(Fuc- -1,4)-GlcNAc- -[Lewis B]-Sp1	13	Maltotetraose- -Sp1
2	Gal- -1,3-GalNAc- -1,4-(Neu5Ac- -2,3)-Gal- -1,4-Glc- -Sp1	5	Gal- -1,4-(6S)GlcNAc- -Sp	8	Neu5Ac- -2,8-Neu5Ac- -2,8-Neu5Ac- -2,3-Gal- -1,4-Glc- -Sp3	11	Gentamicin Sulfate	14	GalNAc- -1,3-(Fuc- -1,2)-Gal- -[Blood A antigen trisaccharide]-Sp1
3	Neu5Gc- -2,3-Gal- -1,3-GlcNAc- -Sp	6	Gal- -1,3-GalNAc- -1,4-(Neu5Gc- -2,3)-Gal- -1,4-Glc- -Sp1	9	Glc- -1,6-Glc- -1,6-Glc- -Sp1	12	Neu5Ac- -2,3-Gal- -1,4-(Fuc- -1,3)-Glc -[3-Sialyl-3-fucosyllactose/F-SL]-Sp1	15	Maltotriose- -Sp1
Cluster3									
1	Fuc- -1,2-Gal- -1,3-GlcNAc- -1,3-Gal- -1,4-Glc- -[LNFP I]-Sp1	3	-Rha-Sp	5	Gal- -1,3-Gal- -1,4-Glc- -Sp	7	Neu5Ac- -2,6-Gal- -1,3-(Neu5Ac- -2,6)-GalNAc- -Sp	9	Gal- -1,4-Glc- -Sp
2	Neu5Ac- -2,8-Neu5Ac- -2,6-Gal- -1,4-Glc-Sp5	4	D-cellose- -Sp1	6	Neomycin trisulfate	8	Kanamycin sulfate	10	Neu5Gc- -2,8-Neu5Ac- -2,3-Gal- -1,4-Glc- -Sp
Cluster4									
1	-Fuc-Sp	11	Gal- -1,4-(Fuc- -1,3)-GlcNAc- -1,3-Gal -Sp1	21	Gal- -1,4-GlcNAc- -1,3-Gal- -1,4-Glc- -[LNnT]-Sp	31	GlcA- -1,4-GlcNAc- -1,4-GlcA- -Sp	41	GlcNAc- -1,4-GlcA- -1,4-GlcNAc- -1,4-GlcA- -Sp
2	Glc- -1,2-Gal- -1,3-Glc- -Sp	12	Neu5Ac- -2,8-Neu5Ac- -2,3-Gal -1,4-Glc- -Sp	22	Neu5Ac- -2,3Gal- -1,4-(6S)GlcNAc- -Sp	32	Gal- -1,3-GlcNAc- -Sp	42	-Gal-Sp
3	Neu5Ac- -2,6-Gal- -1,4-Glc- -Sp	13	Gal- -1,4-GlcNAc- -Sp	23	Gal- -1,3-(Fuc- -1,2)-Gal- -[Blood B antigen trisaccharide]-Sp1	33	Neu5Ac- -2,6-Gal- -1,3-GlcNAc- -Sp	43	-Man-Sp
4	-Glc-Sp	14	-GlcNAc-Sp	24	Gal- -1,3-GalNAc- -Sp	34	Neu5Ac- -2,6-(Neu5Ac- -2,3)-Gal- -1,3-GalNAc- -Sp	44	GlcNAc- -1,6-GlcNAc- -Sp

Ord	Biomarker	Ord	Biomarker	Ord	Biomarker	Ord	Biomarker	Ord	Biomarker
5	Gal- -1,4-Gal- -1,4-Glc- -Sp	15	Neu5Ac- -2,3-Gal- -1,3-(Neu5Ac- -2,6)-GalNAc- -Sp	25	Sisomicin Sulfate	35	Neu5Gc- -2,3-Gal- -1,4-Glc- -Sp	45	-GalNAc-Sp
6	Fuc- -1,2-Gal- -1,4-GlcNAc- -[Blood H antigen trisaccharide]-Sp1	16	GlcNAc- -1,4-GlcNAc- -Sp1	26	Neu5Ac- -2,8-Neu5Gc- -2,3-Gal- -1,4-Glc- -Sp	36	Gal- -1,3-GalNAc- -1,3-Gal- -Sp1	46	Fuc- -1,2-Gal- -1,4-Glc- -[Blood H antigen trisaccharide]-Sp1
7	Neu5Gc- -2,3-Gal- -1,3-(Fuc- -1,4)-GlcNAc- -[Sialyl Lewis A]-Sp	17	Maltohexaose- -Sp1	27	Glc- -1,4-Glc- -Sp1	37	GlcNAc- -1,3-(Glc- -1,2-Glc- -1,2)-Gal -1,3-Glc- -Sp		
8	GlcNAc- -1,6-(Gal- -1,3)-GalNAc- -OSer-Sp4	18	GalNAc- -1,3-(Fuc- -1,2)-Gal- -1,4-Glc -[Blood A antigen tetrose]-Sp1	28	Glc- -1,6-Glc- -1,4-Glc- -Sp1	38	Maltoheptaose- -Sp1		
9	GalNAc- -1,4-GlcNAc- -Sp2	19	Gal- -1,4-Gal- -1,4-GlcNAc- -Sp1	29	GlcNAc- -1,3-Gal- -1,4-Glc- -Sp	39	4-P-GlcNAc- -1,4-Man- -Sp		
10	Neu5Ac- -2,3-Gal- -1,4-(Fuc- -1,3)-GlcNAc- -[Sialyl Lewis X]-Sp	20	Gal- -1,4-Gal- -1,3-GlcNAc- -Sp	30	GalNAc- -1,3-Gal- -1,4-Glc- -Sp	40	Chitin-trisaccharide-Sp1		
Cluster5									
1	Gal- -1,3-(Fuc- -1,4)-GlcNAc- -1,3-Gal -1,4-(Fuc- -1,4)-Glc- -[Lewis A]-Sp1	5	Gal- -1,3-Gal- -Sp1	9	Glc- -1,2-Gal- -Sp	13	GalNAc- -1,4-(Neu5Ac- -2,3)-Gal- -1,4-Glc- -[GM2]-Sp	17	Gal- -1,3-Gal- -1,3-GlcNAc- -Sp
2	-D-Rha-Sp	6	Gal- -1,3-(Fuc- -1,2)-Gal- -1,4-Glc- -[Blood B antigen tetrasaccharide]-Sp1	10	Neu5Ac- -2,6-Gal- -1,3-GalNAc- -Sp	14	GalNAc- -1,4-(Neu5Ac- -2,8-Neu5Ac- -2,3)-Gal- -1,4-Glc- -[GD2]-Sp	18	Neu5Ac- -2,3-Gal- -1,4-Glc- -Sp
3	-D-GlcA-Sp	7	-1,4-Xylotetrose-Sp1	11	Neu5Gc- -2,6-Gal- -1,3-GlcNAc- -Sp	15	Tobramycin		
4	L-pentaguluronic acid- -Sp1	8	Gal- -1,3-GalNAc- -Sp1	12	Neu5Ac- -2,3-Gal- -1,3-GlcNAc- -Sp	16	Gal- -1,3-(Fuc- -1,4)-GlcNAc- -[Lewis A]-Sp		

Appendix Table S12. Glycan motifs in IgA2-targeted clusters

Genus, Species (Order, Cluster)	Abundance vs. control	p-value
Bacteroides, Unknown (*Bacteroidales*, 33)	Increased	$5.97.10^{-15}$
Bacteroides, Unknown (*Bacteroidales*, 56)	Increased	0,011
Lachnoclostridium, Unknown (*Clostridiales*, 122)	Increased	0,019
Unknown *Lachnospirceae,* Unknown (*Clostridiales*, 136)	Increased	0,019
Lachnoclostridium, Unknown (*Clostridiales*, 275)	Increased	0,042
Anaerostipes, Unknown (*Clostridiales*, 34)	Increased	0,011
Blautia, Unknown (*Clostridiales*, 239)	Increased	0,042
Lachnospira, Unknown (*Clostridiales*, 245)	Increased	0,027
Bifidobacterium, Unknown (*Bifidobacteriales*, 25)	Increased	0,019
Butyricicoccus, Unknown (*Clostridiales*, 68)	Increased	0,019
Blautia, Unknown (*Clostridiales*, 102)	Increased	0,019
Faecalibacterium, Unknown (*Clostridiales*, 4)	Increased	0,019
Streptococcus, Streptococcus salivarius (*Lactobacillales*, 275)	Increased	0,043

Appendix Table S13. Differentially recognised species in the IgA1^{+} fraction of the CD stool microbiota

Name	Sequence
NonEUB 338 (Negative control)	ACATCCTACGGGAGGC
EUB338 (Positive control)	GCTGCCTCCCGTAGGAGT
Ato291 (*Collinsella aerofasciens*)	GGTCGGTCTCTCAACCC
Bac303 (*Bacteroides – Prevotella* group)	CCAATGTGGGGGACCTT
Bacto1080 (*Bacteroides vulgatus*)	GCACTTAAGCCGACACCT
Bif164 (*Bifidobacterium* genus)	CATCCGGCATTACCACCC
Bif228 (*Bifidobacterium longum*)	GATAGGACGCGACCCCAT
Erec482 (*Eubacterium rectale-Clostridium coccoides* group)	GCTTCTTAGTCARGGTACCG
Fprau645 (*Faecalibacterium prausnitzii*)	CCTCTGCACTACTCAAGAAAAAC
Lab158 (*Lactobacillus – Enterococcus* group)	GGTATTAGCAYCTGTTTCCA

Appendix Table S14. FISH probe sequences (Eurogentec)
