# Supplementary material for: Alteration of microbiota antibody‐mediated immune selection contributes to dysbiosis in inflammatory bowel diseases
Source: EMBO Mol Med. 2022 Jul 4;14(8):e15386. doi: 10.15252/emmm.202115386 (PMC9358401; doi:10.15252/emmm.202115386)
Supplement: Supplementary file 2 [file EMMM-14-e15386-s002.pdf]

EMBO
Molecular Medicine

Alteration of microbiota antibody-mediated immune selection contributes to dysbiosis in IBD

Eva Michaud, Louis Waeckel, Rémi Gayet, Blandine Chanut, Fabienne Jospin, Katell Bathany, Magali Monnoye, Coraline Genet, Roman Goguyer-Deschaumes, Amelie Prier, Caroline Tokarski, Philippe Gerard, Xavier Roblin, Nicolas Rochereau, and Stéphane Paul
DOI: 10.15252/emmm.202115386

Corresponding author: Stéphane Paul (stephane.paul@chu-st-etienne.fr)

Review Timeline:

Submission Date:	6th Nov 21
Editorial Decision:	17th Dec 21
Revision Received:	21st Apr 22
Editorial Decision:	25th May 22
Revision Received:	15th Jun 22
Accepted:	15th Jun 22

Editor: Zeljko Durdevic

Transaction Report:

(Note: With the exception of the correction of typographical or spelling errors that could be a source of ambiguity, letters and reports are not edited. Depending on transfer agreements, referee reports obtained elsewhere may or may not be included in this compilation. Referee reports are anonymous unless the Referee chooses to sign their reports.)

17th Dec 2021

Dear Prof. Paul,

Thank you for the submission of your manuscript to EMBO Molecular Medicine. We have now received feedback from the three reviewers who agreed to evaluate your manuscript. As you will see from the reports below, the referees acknowledge the interest of the study but also raise important and partially overlapping concerns that should be addressed in a major revision.

Further consideration of a revision that addresses reviewers' concerns in full will entail a second round of review. EMBO Molecular Medicine encourages a single round of revision only and therefore, acceptance or rejection of the manuscript will depend on the completeness of your responses included in the next, final version of the manuscript. For this reason, and to save you from any frustrations in the end, I would strongly advise against returning an incomplete revision.

We would welcome the submission of a revised version within three months for further consideration. Please let us know if you require longer to complete the revision.

Please use this link to login to the manuscript system and submit your revision: Link Not Available

I look forward to receiving your revised manuscript.

Yours sincerely,

Zeljko Durdevic

Zeljko Durdevic
Editor
EMBO Molecular Medicine

*****

***** Reviewer's comments *****

Referee #1 (Comments on Novelty/Model System for Author):

The quality and rigor of the data in many places are low. It is not always clear that they are comparing relevant groups. The authors sometimes seem to have pre-selected populations to test

Referee #1 (Remarks for Author):

Although both Crohns disease (CD) and ulcerative colitis (UC) are characterized as inflammatory bowel diseases (IBD), clinical data shows significant differences in the potential function of IgA in pathogenesis. Using patient cohorts encompassing both active and remittent disease, the authors were able to show changes in the structure and function of IgA1 and IgA2 and their binding to the microbiota. Although there weren't significant differences in the total quantities of IgA and IgA bound bacteria in the stool of their patient cohort, when differentiated into IgA1 and IgA2 CD patients appeared to experience higher levels of IgA1 binding with UC experiencing lower IgA binding overall. These changes appeared to also reflect alterations in the IgA bound bacteria of the stool as characterized by 16S sequencing analysis in which CD and UC microbiota varied modestly. Glycan modifications on IgA has previously been described to be associated with function in other autoimmune pathologies, and the authors identified for the first time differences in the pattern of modifications not only between healthy and IBD patients, but also between CD and UC. However, these glycosylation differences couldn't be directly related to IgA function such as reverse transcytosis (RT) which may alter the ability to interface with the immune system once bound with bacteria. Altogether, while this is a novel topic of study and interesting patient cohort the rigor of the experimentation and analysis often falls short. There are numerous places where the differences that are discovered are exaggerated or not explained clearly, making it difficult for this reviewer to really appreciate what the important findings are. The effect sizes are typically very low and decrease confidence in the biological meanings of the findings. There are also numerous misstatements of the literature. The most major issues are discussed below:

1. A major caveat of this study is the differing number of patient samples used in the analysis due to the limiting quantity of IgA1 that was available across CD and UC patient samples. Although the authors write an explanation for this, they do not specify which of the cohort were included/not included in each analysis and whether this enriched for or underreported active disease over remittent disease. In addition, the non-IBD patient samples were all experiencing irritable intestine syndrome that was not categorized as IBD. Comparison to healthy controls would have given a more complete understanding of the role of IgA in pathogenesis.

2. If figure 1 why doesn't the amount of IgA1(1A) and IgA2(1B) add up to the total IgA (1C)? This reduces my confidence in the findings.

3. The authors observed altered glycosylation patterns on IgA1 and IgA2 between CD and UC and attempted to relate it back to ability to RT ability. Bulk CD IgA1 and IgA2 showed very strong differences in CD IgA1 and IgA2 ability to RT which would indicate that something about the antibody structure was different. Although they were able to relate IgA1 RT to strong binding of dectin-5, they were unable to see changes in IgA2 dectin-5 binding. In the text they explain other potential binding interactions between IgA and M cells but do not follow up. In light of the changes in bacterial binding between IgA1 and IgA2 it would have been interesting to make some connection to glycosylation and bacterial binding.

4. Neutralizing activity of IgA was performed on a single organism (salmonella enterica typhimurium) which undercuts the authors assertion that IgA1 and IgA2 may be performing different functions in the intestine depending on the ability to RT and the type of bound bacteria. This assay does not really make sense - were any of the patients ever infected with Salmonella? If not, why would they neutralize this pathogen? This whole result relies on two timepoints of the control for non IBD IgA2 and it not convincing

5. 16S analysis was performed on magnetically isolated samples which the authors discuss was likely impure fractions of IgA1+ and IgA2+. Although they make some discussion on how binding of IgA1 is sufficient to indicate a phenotype even when in the presence of IgA2, they do not make any more mention of the extremely small percentage of IgA1 single positive bound bacteria so I'm not sure how this argument is possible. For example virtually ALL IgA1 bound bacteria is also bound by IgA2 and there separation does not discriminate the two. Thus, the fact that one sample (IgA1) shows a significant difference while the other does not, should not be possible. Indeed, the stacked abundance charts did not look different to me at all. Why are the total and IgA negative microbiotas sequenced and analyzed as well? Without this this analysis is not meaningful. Finally a DNA dye such as SytoBC should be used to discriminate bacteria in fecal samples.

6. The direct visualization of patient samples by antibody and FISH co-staining was potentiall an important addition to the paper. However, the samples use for this analysis seemed inappropriate. The authors had previously completed all analysis on IgA1 and IgA2 binding in both groups, but for the FISH-IF staining they limited their analysis to CD IgA2 binding. Although the data appeared to show important changes in binding of IgA2 in CD patients, this analysis seems taken out of context and not well controlled.

Referee #2 (Comments on Novelty/Model System for Author):

i would think larger n is required for several of the figures--this is a bit unclear.

Referee #2 (Remarks for Author):

This is an interesting and preliminary study of the IgA subclasses and their binding to microbiota in patients with IBD. The impact of IgA coated microbiota in IBD has been well studied, but the potential differential impact of IgA subclasses in under studied and considered novel. The authors utilize a relatively small cohort of non-IBD, CD and UC samples to define IgA1 and IgA2 levels. They find that CD trends to higher IgA1 and UC with lower IgA2. Functionally, the IgA1 from CD is able to achieve RT whereas IgA2 from UC does not. The authors perform MS of N and O-glycosylations on CD IgA1, but it is unclear if this directly impacts RT. Anti-glycan analysis is provided in figure 3 showing larger differences in UC vs. non-IBD. 16S analysis in a limited cohort (n=6 per group) suggest increased IgA1 coating of SCFA producing bacteria. IgA2 in UC and CD are less neutralizing in a Salmonella infection assay and co-localization suggests increased binding of Eubacterium-Clostridium group.

Although this is an important topic and clinically relevant, the sample size is noted to be a limitation. It is very difficult to ascertain how many samples were tested in the different figures. The glycosylation analysis is very exciting but the direct relationship to RT, antigen binding, or neutralization was not tested.

Specific comments:
1. For Figure 1A/B, the data appear bimodal (high vs. low)? Is that meaningful? Also, for Fig. 1F, difference appears driven by a single outlier only.
2. For 1G-N, how many samples are analyzed? Can the dots be shown to be clear? How many weak or strong binders?
3. Figure 5, is it clear that the neutralization is meaningful in 5B? Can the authors show positive and negative controls to give a sense of real neutralization.
4. What is the relationship between the anti-glycan profiles (figure 3) and the altered IgA2 neutralization (Figure 5), microbiome binding (figure 4) or FISH (figure 6). Can any of this be tested mechanistically?
5. What is the relationship between glycosylation (figure 2) and RT data presented in figure 1. Is it causative?
6. Need to be much more clear about the n evaluated in each figure.

Referee #3 (Comments on Novelty/Model System for Author):

Michaud et al. in their manuscript entitled "Alteration of microbiota antibody-mediated immune selection contributes to dysbiosis in IBD" present a comprehensive analysis of secretory immunoglobulins in IBD and show that IgA1 and IgA2 functionality is altered in CD vs UC. In the context of CD, IgA1 at least as measured in stool gains retro-transport ability. IgA1s in CD display increased glycan reactivity and select distinct microbes. On the other hand, UC stool samples lose IgA2 retro-transport function and display impaired glycan reactivity. These data provide a novel take on the impact of different IgA subclasses, their glycan reactivity and selection in IBD and may impact clinical practice as highlighted by the authors.

The weaknesses of the manuscript which hopefully are easily addressed include:
1. the small cohort of patients analyzed that should be discussed in particularly. Of the non-IBD group (N=8).
2. Clinical manifestations and affected tissues are different in CD and UC which may ultimately influence IgA subclasses, their distribution and function. Given that IgA1 is less abundant in the distal intestine and colon, the authors should provide a discussion of its implications. The authors attempt this here "The absence of altered abundance in the IgA2-bound fraction of the

microbiota was surprising given the major role IgA2 plays in antigen sampling under homeostatic conditions and its dominance over IgA1 in the colon" but this should be done incorporating all their data in the end.
Given that microbes bound by IgA1 but not are different in CD (Fig 4) but local responses of IgA2 are altered, how does selection of microbes by IgA1 and IgA2 work locally? Can authors speculate on a working model? What happens to local IgA1 responses? Is the CD IgA1 biased selection the microbiota preserved locally?
3. Are there any changes in microbial species identified by 16S in non-IgA bound (IgA1-neg and IgA2-neg) in CD and UC?
4. Minor: gating strategy in Fig S1 is a bit confusing. Could authors utilize a serial gating flow clarified by arrows?
5. Supplementary Table S2 suggests a correlation with IgG levels that's shows the opposite trend in non-IBD vs UC that was largely absent in CD? Could authors comment on this? What drives this correlation since it is not there for IgA1 or IgA2? Is it present only on a subset of UC patients.

Revision EMM-2021-15386

Dear Editor and Reviewers,
Thank you for taking the time to review this article. We greatly appreciate your work to improve our manuscript. We have considered all your comments and areas for improvement. We sincerely hope that this revised version is clearer and will meet your expectations. Please find below a point-by-point response to each of the referee comments and a description of changes made.

Referee #1 (Comments on Novelty/Model System for Author):
The quality and rigor of the data in many places are low. It is not always clear that they are comparing relevant groups. The authors sometimes seem to have pre-selected populations to test.

We have clarified the data set and indicated our experimental approaches wherever necessary.

Referee #1 (Remarks for Author):
Although both Crohns disease (CD) and ulcerative colitis (UC) are characterized as inflammatory bowel diseases (IBD), clinical data shows significant differences in the potential function of IgA in pathogenesis. Using patient cohorts encompassing both active and remittent disease, the authors were able to show changes in the structure and function of IgA1 and IgA2 and their binding to the microbiota. Although there weren't significant differences in the total quantities of IgA and IgA bound bacteria in the stool of their patient cohort, when differentiated into IgA1 and IgA2 CD patients appeared to experience higher levels of IgA1 binding with UC experiencing lower IgA binding overall. These changes appeared to also reflect alterations in the IgA bound bacteria of the stool as characterized by 16S sequencing analysis in which CD and UC microbiota varied modestly. Glycan modifications on IgA has previously been described to be associated with function in other autoimmune pathologies, and the authors identified for the first time differences in the pattern of modifications not only between healthy and IBD patients, but also between CD and UC. However, these glycosylation differences couldn't be directly related to IgA function such as reverse transcytosis (RT) which may alter the ability to interface with the immune system once bound with bacteria. Altogether, while this is a novel topic of study and interesting patient cohort the rigor of the experimentation and analysis often falls short. There are numerous places where the differences that are discovered are exaggerated or not explained clearly, making it difficult for this reviewer to really appreciate what the important findings are. The effect sizes are typically very low and decrease confidence in the biological meanings of the findings. There are also numerous misstatements of the literature. The most major issues are discussed below:

Thanks for these remarks. We have rewritten many passages of our results and discussion to clarify our data as much as possible and to reduce over-interpretation.

1. A major caveat of this study is the differing number of patient samples used in the analysis due to the limiting quantity of IgA1 that was available across CD and UC patient samples. Although the authors write an explanation for this, they do not specify which of the cohort were included/not included in each analysis and whether this enriched for or underreported active disease over remittent disease. In addition, the non-IBD patient samples were all experiencing irritable intestine syndrome that was not categorized as IBD. Comparison to healthy controls would have given a more complete understanding of the role of IgA in pathogenesis.

We fully understand this remark and apologize for the lack of clarity in the description of our patient cohorts. In particular, our control cohort does not include IBS patients. These are patients who came for a colonoscopy to diagnose cancer and whose colonoscopy did not reveal any lesion or inflammatory syndrome. The biological workup of these negative controls is totally normal.

2. If figure 1 why doesn't the amount of IgA1(1A) and IgA2(1B) add up to the total IgA (1C)? This reduces my confidence in the findings.

We have modified the scale of Fig. 1C to make it more comparable to 1A and 1B, and in doing so realized our statistical analysis had not been run on the proper dataset. Fig. 1C does indeed show statistical significance between CD and UC total IgA concentration and between Non-IBD and UC. We apologize for this mistake. In addition, we have compiled some raw data in a table below to compare expected values and actual values. They do not differ from more than 19%, which is the highest.

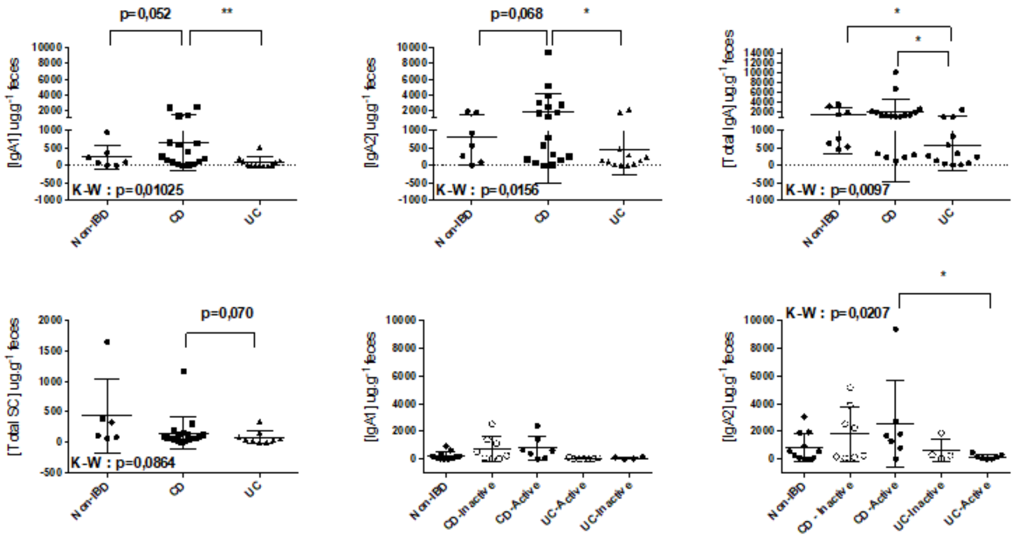

Patient ID	IgA1 (ug/mL)	IgA2 (ug/mL)	Theoric total	Actual total	Delta
**Non-IBD 1**	100	271	371	460,2072	19,3841383
**CD2**	659	804	1463	1400,222	-4,4834319
**CD10**	599	1303	1902	2056,552	7,51510295
**UC10**	524	2229	2753	2578,088	-6,7845628
**UC11**	213	103	316	339,9892	7,05587119

However if you prefer we can also put the data on total IgA in supplemantary.

3. The authors observed altered glycosylation patterns on IgA1 and IgA2 between CD and UC and attempted to relate it back to ability to RT ability. Bulk CD IgA1 and IgA2 showed very strong differences in CD IgA1 and IgA2 ability to RT which would indicate that something about the antibody structure was different. Although they were able to relate IgA1 RT to strong binding of dectin-5, they were unable to see changes in IgA2 dectin-5 binding. In the text they explain other potential binding interactions between IgA and M cells but do not follow up. In light of the changes in bacterial binding between IgA1 and IgA2 it would have been interesting to make some connection to glycosylation and bacterial binding.

We totally agree with this important comment/suggestion. We have previously evidenced that de-glycosylation or de-sialylation of IgA inhibit RT (Rochereau, et al. PLoS Biol, 2013). In the present study we also show the same observation towards de-sialylation of these IgA1 in both CD and UC. This implicates two main things: 1) Sialylation have a general immunosuppressive function and that remains true for IgA (Streffen et al., 2020), their removal would thus polarize immune response towards inflammation; 2) Siglec-5, the co-receptor for IgA2 RT is a sialic acid receptor, therefore there may be either another co-receptor recruited for IgA1 RT in CD or another receptor/co-receptor system (such as the IgA1-CD71 binding observed in coeliac disease) and this has not been described in IBD to date. The discussion on that topic has been reorganized to bring more emphasis to the reliance on glycan interactions for IgA-mediated selection of the microbiota.

4. Neutralizing activity of IgA was performed on a single organism (salmonella enterica typhimurium) which undercuts the authors assertion that IgA1 and IgA2 may be performing different functions in the intestine depending on the ability to RT and the type of bound bacteria. This assay does not really make sense - were any of the patients ever infected with Salmonella? If not, why would they neutralize this pathogen? This whole result relies on two time points of the control for non IBD IgA2 and it not convincing.

We agree again with this really important comment. SIgA have dual function at mucosal surface: 1) selection of commensals and 2) elimination of pathogens. Both are known to exploit IgA polyreactivity, either to immobilize the bacteria in the mucus (commensal) or to inhibit its virulence. Polyreactivity of microbiota-binding IgA was notably evidencedpreviously (Bunker, et al. Science 2017). Here we wanted to assess the functionality of IgA as broadly neutralizing antibodies and how they differed in IBD. We simply aimed at modelizing IgA-pathogen interaction to evidence modifications of functionality in subclasses of IgA. The rationale was thus not to assess patient's responses with regards to a history of salmonella infections. In addition, Bunker, et al. Science (2017) show that microbiota-binding IgA had enhanced reactivity to microbial glycans, including Salmonella typhimurium. We agree that using only a pathogen may have made our conclusions limited, so we also performed those experiments on a commensal E. coli strain. To that experiment we also added deglycosylated patient's samples (the same IgA used in the initial assay were treated with PNGase and Neuraminidase) to address links between glycosylation and selection of the microbiota (Fig 5. F-I). When taking into account previous results from this study, in our cohort, deglycosylation of CD IgA2 appears to impair the regulation of commensal growth, rendering IgA2 more bacteriostatic for commensals but not pathogens in CD. We think this an important observation.

5. 16S analysis was performed on magnetically isolated samples which the authors discuss was likely impure fractions of IgA1+ and IgA2+. Although they make some discussion on how binding of IgA1 is sufficient to indicate a phenotype even when in the presence of IgA2, they do not make any more mention of the extremely small percentage of IgA1 single positive bound bacteria so I'm not sure how this argument is possible. For example virtually ALL IgA1 bound bacteria is also bound by IgA2 and there separation does not discriminate the two. Thus, the fact that one sample (IgA1) shows a significant difference while the other does not, should not be possible. Indeed, the stacked abundance charts did not look different to me at all.

Literature on IgA1 coating of gut microbes remains limited. In our protocol, the 'IgA1^{+}' fraction may indeed be contaminated by double positive bacteria, but we still get rid of single IgA2^{+} bacteria, thus limiting IgA2-associated variability. That 'IgA1^{+}' fraction thus contains both single positive and double positive bacteria, and it very well may be that IgA1 coating alone, might counter balance the effect of IgA2, especially if its structure, such as glycosylations, is already altered in CD.

Additionally, we have looked at MFI (Median Fluorescence Intensity) for IgA1 and IgA2 in double-positive bacteria, and while there was not significantly more IgA1 bound to these bacteria, there was significantly less IgA2, which meant there was a lower IgA/IgA1 ratio in CD on those bacteria, effectively favoring IgA1 binding and selection.

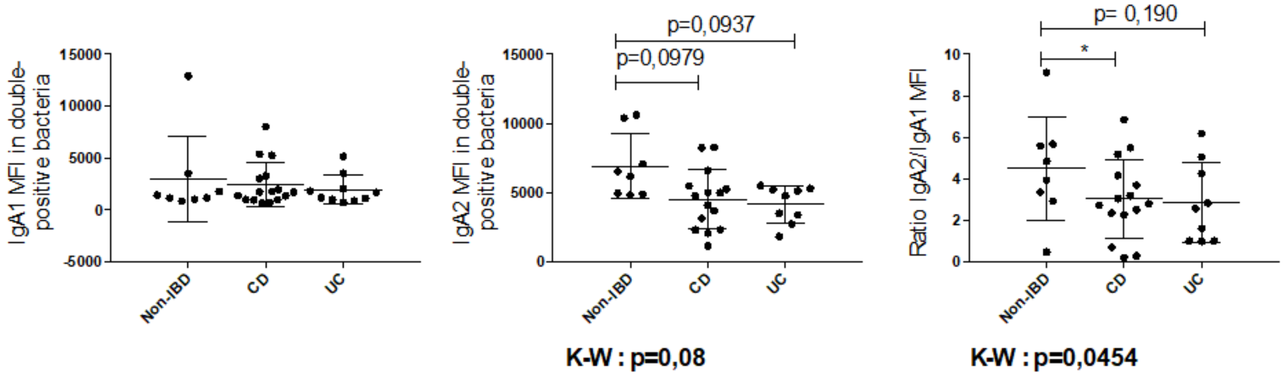

We have isolated the single IgA1-positive data in a graph shown below and added in suppl. Fig. 4D. Scales for A and B were also modified to facilitate reading. Again, single IgA1^{+} bacteria seem to form two cluster of high and low frequency in CD, as was the case for IgA concentrations. Why are the total and IgA negative microbiotas sequenced and analyzed as well? Total microbiota was sequenced and is presented in Suppl. Fig. 4. Without this this analysis is not meaningful. Finally, a DNA dye such as SytoBC should be used to discriminate bacteria in fecal samples. DAPI was used as a DNA marker in the flow cytometry experiments as described by Zimmerman, et al. EJI (2016). This is clarified in Suppl fig. 1, as the flow cytometry gating strategy initially did not make it obvious.

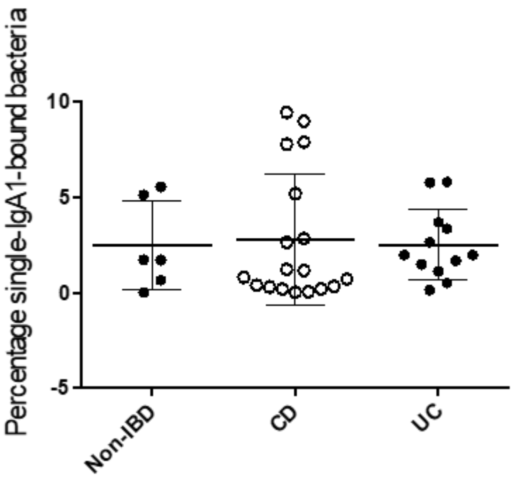

GIMAP EA 3064
Campus Santé Innovations
Faculté de Médecine Jacques Lisfranc
10, rue de la Marandière
42270 Saint-Priest-en-Jarez
(33)04 77 42 14 67
gimap@univ-st-etienne.fr
gimap.univ-st-etienne.fr

6. The direct visualization of patient samples by antibody and FISH co-staining was potentially an important addition to the paper. However, the samples use for this analysis seemed inappropriate. The authors had previously completed all analysis on IgA1 and IgA2 binding in both groups, but for the FISH-IF staining they limited their analysis to CD IgA2 binding. Although the data appeared to show important changes in binding of IgA2 in CD patients, this analysis seems taken out of context and not well controlled.

We totally agree with this comment. We did perform IgA1 staining on CD patients, however despite using the very same staining protocol and repetitions of the experiments, the IgA1 staining remain very poor (we provide examples of this below). We suspect IgA1 concentration in the colon may just be too limited compared to that of IgA2 as it has been described in the literature.

Representative image of FISH-IF staining for non-IBD and Crohn gut biopsies. The red 16S-FISH channel was removed to evidence IgA1 and IgA2 staining and images were overlayed with phase contrast snapshots. Scale bar (bottom right picture) represents 20µm. Crohn samples for both IgA1 and IgA2 staining were treated the same day, and non-IBD samples at a later date, for similar outcome in IgA1 staining. Contrasts for green staining were pushed a little more so images would

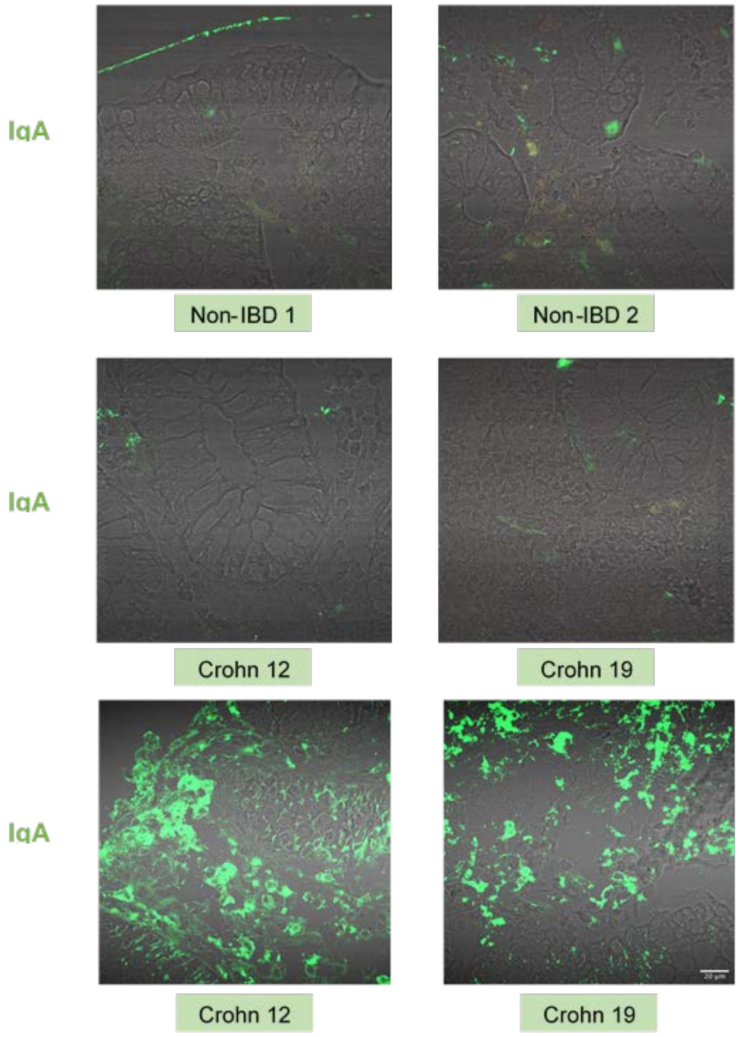

Referee #2 (Comments on Novelty/Model System for Author):

i would think larger n is required for several of the figures--this is a bit unclear.

We have added the n in the figures. The purification of IgA1 IgA2 fractions is not always possible depending on the samples. The methodology is very cumbersome and that is why the number of samples is limited in this study.

This is an interesting and preliminary study of the IgA subclasses and their binding to microbiota in patients with IBD. The impact of IgA coated microbiota in IBD has been well studied, but the potential differential impact of IgA subclasses in under studied and considered novel. The authors utilize a relatively small cohort of non-IBD, CD and UC samples to define IgA1 and IgA2 levels. They find that CD trends to higher IgA1 and UC with lower IgA2. Functionally, the IgA1 from CD is able to achieve RT whereas IgA2 from UC does not. The authors perform MS of N and O-glycosylations on CD IgA1, but it is unclear if this directly impacts RT. Anti-glycan analysis is provided in figure 3 showing larger differences in UC vs. non-IBD. 16S analysis in a limited cohort (n=6 per group) suggest increased IgA1 coating of SCFA producing bacteria. IgA2 in UC and CD are less neutralizing in a Salmonella infection assay and co-localization suggests increased binding of Eubacterium-Clostridium group. Although this is an important topic and clinically relevant, the sample size is noted to be a limitation. It is very difficult to ascertain how many samples were tested in the different figures. The glycosylation analysis is very exciting but the direct relationship to RT, antigen binding, or neutralization was not tested.

We thank the reviewer for this positive opinion on our study. The number of patients tested is indeed limited and this is a limitation of the study. However, it must be underlined that the methodology to purify these IgA1 and IgA2 sub-fractions is very cumbersome and not always possible depending on the samples. In spite of the limited number, we were able to highlight important new data on the differential role of the IgA subclasses.

Specific comments:
1. For Figure 1A/B, the data appear bimodal (high vs. low)? Is that meaningful? Also, for Fig. 1F, difference appears driven by a single outlier only.

We also noticed this 'clustering' in our data, and have tried to correlate it with age, disease duration, Ig concentration or % bound bacteria in the microbiota, but none of these factors were associated to these sub-group (Suppl. table. 2). We also looked at whether the degree of inflammation could account for these differences. Unfortunately, we do not always have comparable biological data to draw a clear conclusion, despite an interesting trend. This is really an important point that we will try to explore on a new cohort.

2. For 1G-N, how many samples are analyzed? Can the dots be shown to be clear? How many weak or strong binders?

The figure has been modified accordingly. Total numbers of patients for which we had purified IgA1 and IgA2 are mentioned in the legends and of these figures and were respectively:

IgA1

- ***Non-IBD: 3 strong and 3 weak binders***
- ***CD: 2 strong and 6 weak binders***
- ***UC: 0 strong and 4 weak binders***

IgA2

- ***Non-IBD: 4 strong and 1 weak binders***
- ***CD: 5 strong and 3 weak binders***
- ***UC: 0 strong and 4 weak binders***
-

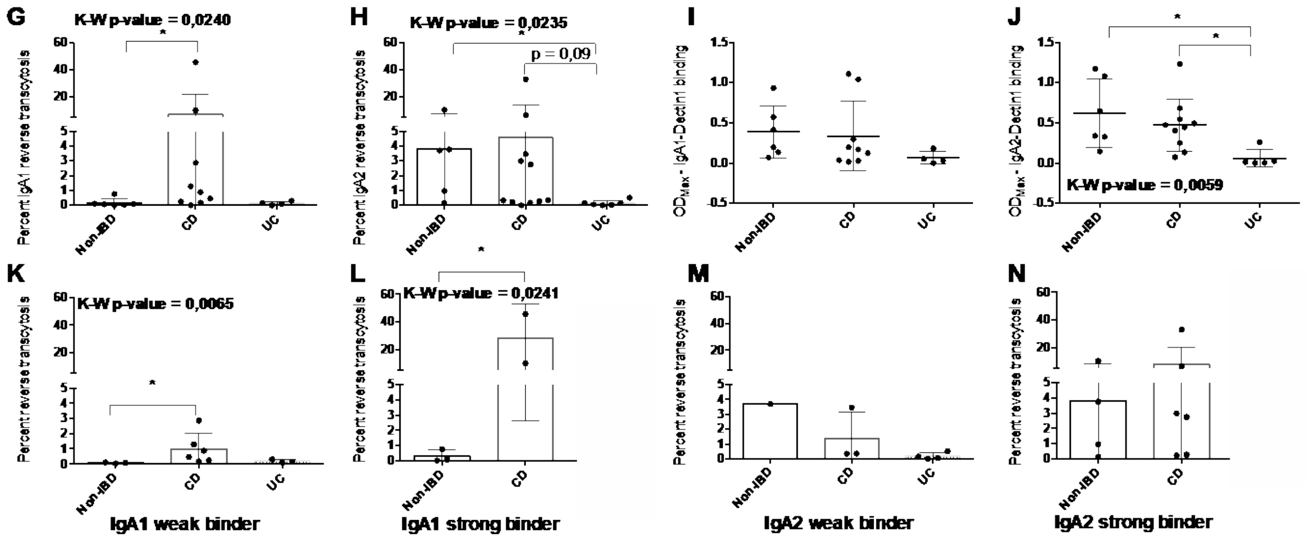

3. Figure 5, is it clear that the neutralization is meaningful in 5B? Can the authors show positive and negative controls to give a sense of real neutralization.

The figure 5 has been totally modified and we added also neutralization of a commensal strain as suggested by the reviewer 1. A no antibody condition was added to this test initially (no effect of the growth), it was just removed to facilite reading the figures. No human neutralizing IgA as a positive control was available at the time. As can be seen above, Only Non-IBD IgA2 was significantly more neutralizing than the negative control: CD and UC IgA1 and IgA2 were not.

4. What is the relationship between the anti-glycan profiles (figure 3) and the altered IgA2 neutralization (Figure 5), microbiome binding (figure 4) or FISH (figure 6). Can any of this be tested mechanistically?

Very good point. We performed co-culture assays with a commensal E. coli after de-glycosylation of IgA and show that non-IBD IgA1 require glycosylation for bacteria neutralization while CD IgA1 does not (Fig. 5 F-I). When studying IgA polyreactivity, Bunker, et al. Science (2017) have shown the IgA can be microbiota-binding or non-microbiota binding. They notably demonstrate that microbiota-binding IgA had enhanced reactivity to microbial glycans, including Salmonella Typhimurium.

5. What is the relationship between glycosylation (figure 2) and RT data presented in figure 1. Is it causative?

Previous work from our team evidences the link between glycosylation of the IgA and their ability to undergo RT (Rochereau, et al. PLoS Biol, 2013); wherein de-glycosylated or mutant IgA2 lost its ability for RT. This was dependent on Dectin-1 and siglec-5 binding, both of which bind IgA glycans. We have performed the FAE RT model using de-glycosylated IgA from CD patients (from the same patients that were used in the present study and in the RT experiments) as shown below. Results are expressed as Fold change from non-IBD RT. While the results do not show statistical significance when comparing glycosylated vs deglycosylated there is a tendency to lower RT levels in de-glycosylated IgA, which confirms on a small number of patients results that were previously obtained with commercial IgA. We suspect, in the context of IBD, that there are distinct functions associated to either fecal IgA1 or tissular IgA2.

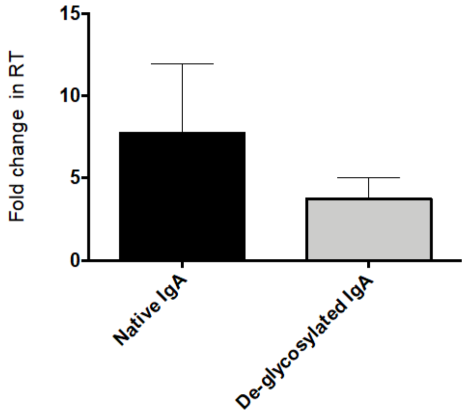

6. Need to be much more clear about the n evaluated in each figure.

Thank you for this comment, n have been added in the graph themselves when possible, and precisions on patients' sample exclusion have been added to the legends as well as the material and methods. This legibility problem on the n is particularly evident in Fig. 1 and fig. 4 so we have mostly modified these ones accordingly.
Please note that regardless of the change in number of samples, it was always the same patients throughout all experiments which is also very crucial. If ever one couldn't be used (usually because of low concentrations post-purification, this was very frequent with IgA1

and most UC samples), we accepted a lower n rather than add an unknown sample for which we may not have that amount of information. We never randomly added an IBD sample to complete the dataset so as to keep all correlations consistent within that small group of patients. We think that seems clear from the material and methods but we can add more precisions if reviewers feel this needs to be made more obvious.

Referee #3 (Comments on Novelty/Model System for Author):

Michaud et al. in their manuscript entitled "Alteration of microbiota antibody-mediated immune selection contributes to dysbiosis in IBD" present a comprehensive analysis of secretory immunoglobulins in IBD and show that IgA1 and IgA2 functionality is altered in CD vs UC. In the context of CD, IgA1 at least as measured in stool gains retro-transport ability. IgA1s in CD display increased glycan reactivity and select distinct microbes. On the other hand, UC stool samples lose IgA2 retro-transport function and display impaired glycan reactivity. These data provide a novel take on the impact of different IgA subclasses, their glycan reactivity and selection in IBD and may impact clinical practice as highlighted by the authors.

We thank the reviewer for this very positive assessment.

The weaknesses of the manuscript which hopefully are easily addressed include:
1. the small cohort of patients analyzed that should be discussed in particularly. Of the non-IBD group (N=8).

The number of patients tested is indeed limited and this is a limitation of the study. However, it must be underlined that the methodology to purify these IgA1 and IgA2 sub-fractions is very cumbersome and not always possible depending on the samples. In spite of the limited number, we were able to highlight important new data on the differential role of the IgA subclasses.

2. Clinical manifestations and affected tissues are different in CD and UC which may ultimately influence IgA subclasses, their distribution and function. Given that IgA1 is less abundant in the distal intestine and colon, the authors should provide a discussion of its implications.

This is a very good point and we have added this point in our discussion.

Given that microbes bound by IgA1 but not are different in CD (Fig 4) but local responses of IgA2 are altered, how does selection of microbes by IgA1 and IgA2 work locally? Can authors speculate on a working model? What happens to local IgA1 responses? Is the CD IgA1 biased selection the microbiota preserved locally?

Very good point. We have proposed a working hypothetical model in Figure 7. We did perform IgA1 staining on CD patients, however despite using the very same staining protocol and repetitions of the experiments, the IgA1 staining remain very poor (see reviewer 1). We suspect IgA1 concentration in the colon may just be too limited compared to that of IgA2 as it has been described in the literature.

3. Are there any changes in microbial species identified by 16S in non-IgA bound (IgA1-neg and IgA2-neg) in CD and UC?

We agree with the reviewer that changes in microbial species in non-IgA bound fractions would be relevant additional informations. In our study, we have analyzed total microbiota and IgA-bound fraction only. Therefore we would need to know the exact part of the total microbiota which is IgA bound in our samples to deduct them from total microbiota and then determine the non-IgA bound fraction changes. Because it is known that the part of the microbiota which is IgA bound may greatly variate, we believe that it would be too speculative to perform such analysis whose results would be inaccurate (Pabst et al., Nat Rev Hep. Gastro. 2022; Goguyer-Deschaumes et al., Trends Immunol. 2022; Rollenske et al., Nature 2021.)

4. Minor: gating strategy in Fig S1 is a bit confusing. Could authors utilize a serial gating flow clarified by arrows?

Yes of course.

5. Supplementary Table S2 suggests a correlation with IgG levels that's shows the opposite trend in non-IBD vs UC that was largely absent in CD? Could authors comment on this? What drives this correlation since it is not there for IgA1 or IgA2? Is it present only on a subset of UC patients.

This is an interesting point, our opinion is that IgG in UC stools are supplemented by seric IgG that 'contaminates' the inflammation site due to the high level of ulceration. We thus suspect increased IgG would correlate with increased IgA levels as a result of increased disease activity. Suppl. fig. 4C shows increased levels of IgA-bound bacteria in patient with active disease that suggest disease activity correlates with functionality.

We deeply hope that the point-by-point reply to the Reviewers, will provide convincing evidence of the importance of the role of our datas in the physiopathology of IBD.

Sincerely,

Stéphane PAUL

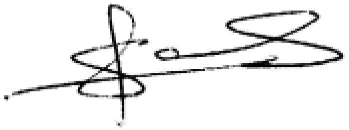

GIMAP EA 3064
Campus Santé Innovations
Faculté de Médecine Jacques Lisfranc
10, rue de la Marandière
42270 Saint-Priest-en-Jarez

(33)04 77 42 14 67
gimap@univ-st-etienne.fr
gimap.univ-st-etienne.fr

25th May 2022

Dear Prof. Paul,

Thank you for the submission of your revised manuscript to EMBO Molecular Medicine. I am pleased to inform you that we will be able to accept your manuscript pending the following final amendments:

1) In the main manuscript file, please do the following:
- Limit keywords to max 5.
- Remove text highlight colour.
- In M&M, you have deleted the paragraph "Analysis of the IgA1- and IgA2-coated microbial community using sequencing", however, you show the results of 16S RNA sequencing in Figure 4 and refer to it in the abstract and results. Please clarify and correct.
- Indicate in legends exact p= values, not a range, along with the statistical test used. Also, please define nature of replicates biological or technical for all experiments.
- In M&M, add statistical paragraph that should reflect all information that you have filled in the Authors Checklist, especially regarding randomization, blinding, replication.
- In M&M, include a statement that informed consent was obtained from all human subjects and that in addition to the WMA Declaration of Helsinki the experiments also conformed to the principles set out in the Department of Health and Human Services Belmont Report.
- Raw data from large-scale datasets (16S RNA sequencing) should be deposited in one of the relevant databases and made freely available prior the publication of the manuscript. Use the following format to report the accession number of your data:

The datasets produced in this study are available in the following databases:
[data type]: [full name of the resource] [accession number/identifier] ([doi or URL or identifiers.org/DATABASE:ACCESSION])

Please check "Author Guidelines" for more information.
https://www.embopress.org/page/journal/17574684/authorguide#availabilityofpublishedmaterial
-
2) Author Checklist: We updated the "Author Checklist". Please complete the new version and submit it with the revised manuscript. You can find it here: https://www.embopress.org/page/journal/17574684/authorguide
3) Appendix: Please add Table of Content and check callouts of Appendix Supplementary Figures and Tables in the main text.
4) Synopsis: Every published paper now includes a 'Synopsis' to further enhance discoverability. Synopses are displayed on the journal webpage and are freely accessible to all readers. They include separate synopsis image and synopsis text.
- Synopsis text: Please provide a short stand first (maximum of 300 characters, including space) as well as 2-5 one sentence bullet points that summarise the paper as a .doc file. Please write the bullet points to summarise the key NEW findings. They should be designed to be complementary to the abstract - i.e. not repeat the same text. We encourage inclusion of key acronyms and quantitative information (maximum of 30 words / bullet point). Please use the passive voice.
- Synopsis image: Please provide a striking image or visual abstract as a high-resolution jpeg file 550 px-wide x (250-400)-px high to illustrate your article.
- Please check your synopsis text and image and submit their final versions with your revised manuscript. Please be aware that in the proof stage minor corrections only are allowed (e.g., typos).
5) For more information: There is space at the end of each article to list relevant web links for further consultation by our readers. Could you identify some relevant ones and provide such information as well? Some examples are patient associations, relevant databases, OMIM/proteins/genes links, author's websites, etc...
6) Source data: We encourage you to include the source data for figure panels that show essential data. Numerical data should be provided as individual .xls or .csv files (including a tab describing the data). For blots or microscopy, uncropped images should be submitted (using a zip archive if multiple images need to be supplied for one panel). Please check "Author Guidelines" for more information. https://www.embopress.org/page/journal/17574684/authorguide#sourcedata
7) As part of the EMBO Publications transparent editorial process initiative (see our Editorial at http://embomolmed.embopress.org/content/2/9/329), EMBO Molecular Medicine will publish online a Review Process File (RPF) to accompany accepted manuscripts. This file will be published in conjunction with your paper and will include the anonymous referee reports, your point-by-point response and all pertinent correspondence relating to the manuscript. Let us know whether you agree with the publication of the RPF and as here, if you want to remove or not any figures from it prior to publication. Please note that the Authors checklist will be published at the end of the RPF.
8) Please provide a point-by-point letter INCLUDING my comments as well as the reviewer's reports and your detailed responses (as Word file).

I look forward to reading a new revised version of your manuscript as soon as possible.

Yours sincerely,

Zeljko Durdevic

Zeljko Durdevic
Editor
EMBO Molecular Medicine

***** Reviewer's comments *****

Referee #3 (Comments on Novelty/Model System for Author):

The authors have greatly improved the manuscript and increased transparency of their data. In this revised version, relevant controls are included, data are re-analyzed where necessary, technical limitations of the study are stated and some figures are completely reconfigured to better characterize function of SIgA in the context of CD and UC. Although the dataset is still rather small, they now clearly discuss limitations of their results, provide relevant discussion and highlight implications of their findings. With all revised material included, the manuscript is suitable for publication.

The authors performed the requested editorial changes.

We are pleased to inform you that your manuscript is accepted for publication and is now being sent to our publisher to be included in the next available issue of EMBO Molecular Medicine. Please be aware that all deposited datasets should be made freely available upon acceptance, without restriction.

EMBO Press Author Checklist

Corresponding Author Name: Stephane Paul	
Journal Submitted to: EMBO Molecular Medicine	
Manuscript Number:	

USEFUL LINKS FOR COMPLETING THIS FORM
The EMBO Journal - Author Guidelines
EMBO Reports - Author Guidelines
Molecular Systems Biology - Author Guidelines
EMBO Molecular Medicine - Author Guidelines

Reporting Checklist for Life Science Articles (updated January 2022)
This checklist is adapted from Materials Design Analysis Reporting (MDAR) Checklist for Authors. MDAR establishes a minimum set of requirements in transparent reporting in the life sciences (see Statement of Task: 10.31222/osf.io/9sm4x). Please follow the journal's guidelines in preparing your manuscript.
Please note that a copy of this checklist will be published alongside your article.

Abridged guidelines for figures
1. Data
The data shown in figures should satisfy the following conditions:
- the data were obtained and processed according to the field's best practice and are presented to reflect the results of the experiments in an accurate and unbiased manner.
- ideally, figure panels should include only measurements that are directly comparable to each other and obtained with the same assay.
- plots include clearly labeled error bars for independent experiments and sample sizes. Unless justified, error bars should not be shown for technical replicates.
- if n<5, the individual data points from each experiment should be plotted. Any statistical test employed should be justified.
- Source Data should be included to report the data underlying figures according to the guidelines set out in the authorship guidelines on Data Presentation.

2. Captions
Each figure caption should contain the following information, for each panel where they are relevant:
- a specification of the experimental system investigated (eg cell line, species name).
- the assay(s) and method(s) used to carry out the reported observations and measurements.
- an explicit mention of the biological and chemical entity(ies) that are being measured.
- an explicit mention of the biological and chemical entity(ies) that are altered/varied/perturbed in a controlled manner.
- the exact sample size (n) for each experimental group/condition, given as a number, not a range;
- a description of the sample collection allowing the reader to understand whether the samples represent technical or biological replicates (including how many animals, litters, cultures, etc.).
- a statement of how many times the experiment shown was independently replicated in the laboratory.
- definitions of statistical methods and measures:
  - common tests, such as t-test (please specify whether paired vs. unpaired), simple χ2 tests, Wilcoxon and Mann-Whitney tests, can be unambiguously identified by name only, but more complex techniques should be described in the methods section;
  - are tests one-sided or two-sided?
  - are there adjustments for multiple comparisons?
  - exact statistical test results, e.g., P values = x but not P values < x;
  - definition of 'center values' as median or average;
  - definition of error bars as s.d. or s.e.m.

Please complete ALL of the questions below.
Select "Not Applicable" only when the requested information is not relevant for your study.

Materials

Newly Created Materials	Information included in the manuscript?	In which section is the information available? (Reagents and Tools Table, Materials and Methods, Figures, Data Availability Section)
New materials and reagents need to be available; do any restrictions apply?	Not Applicable	

Antibodies	Information included in the manuscript?	In which section is the information available? (Reagents and Tools Table, Materials and Methods, Figures, Data Availability Section)
For **antibodies** provide the following information:<br>- Commercial antibodies: RRID (if possible) or supplier name, catalogue number and or/clone number<br>- Non-commercial: RRID or citation	Yes	Materials and Methods

DNA and RNA sequences	Information included in the manuscript?	In which section is the information available? (Reagents and Tools Table, Materials and Methods, Figures, Data Availability Section)
**Short novel DNA or RNA including primers, probes**: provide the sequences.	Yes	Materials and Methods

Cell materials	Information included in the manuscript?	In which section is the information available? (Reagents and Tools Table, Materials and Methods, Figures, Data Availability Section)
**Cell lines:** Provide species information, strain. Provide accession number in repository **OR** supplier name, catalog number, clone number, and/**OR** RRID.	Select response	
**Primary cultures:** Provide species, strain, sex of origin, genetic modification status.	Select response	
Report if the cell lines were recently **authenticated** (e.g., by STR profiling) and tested for mycoplasma contamination.	Not Applicable	

Experimental animals	Information included in the manuscript?	In which section is the information available? (Reagents and Tools Table, Materials and Methods, Figures, Data Availability Section)
**Laboratory animals or Model organisms:** Provide species, strain, sex, age, genetic modification status. Provide accession number in repository **OR** supplier name, catalog number, clone number, **OR** RRID.	Not Applicable	
**Animal observed in or captured from the field:** Provide species, sex, and age where possible.	Not Applicable	
Please detail **housing and husbandry conditions**.	Not Applicable	

Plants and microbes	Information included in the manuscript?	In which section is the information available? (Reagents and Tools Table, Materials and Methods, Figures, Data Availability Section)
**Plants:** provide species and strain, ecotype and cultivar where relevant, unique accession number if available, and source (including location for collected wild specimens).	Not Applicable	
**Microbes:** provide species and strain, unique accession number if available, and source.	Yes	Materials and Methods

Human research participants	Information included in the manuscript?	In which section is the information available? (Reagents and Tools Table, Materials and Methods, Figures, Data Availability Section)
If collected and within the bounds of privacy constraints report on age, sex and gender or ethnicity for all study participants.	Yes	Materials and Methods + Appendix Table S1

Core facilities	Information included in the manuscript?	In which section is the information available? (Reagents and Tools Table, Materials and Methods, Figures, Data Availability Section)
If your work benefited from core facilities, was their service mentioned in the acknowledgments section?	Not Applicable	

Design

Study protocol	Information included in the manuscript?	In which section is the information available? (Reagents and Tools Table, Materials and Methods, Figures, Data Availability Section)
If study protocol has been **pre-registered, provide DOI in the manuscript**. For clinical trials, provide the trial registration number **OR** cite DOI.	Not Applicable	
Report the **clinical trial registration number** (at ClinicalTrials.gov or equivalent), where applicable.	Not Applicable	

Laboratory protocol	Information included in the manuscript?	In which section is the information available? (Reagents and Tools Table, Materials and Methods, Figures, Data Availability Section)
Provide DOI OR other citation details if **external detailed step-by-step protocols** are available.	Yes	Materials and Methods

Experimental study design and statistics	Information included in the manuscript?	In which section is the information available? (Reagents and Tools Table, Materials and Methods, Figures, Data Availability Section)
Include a statement about **sample size** estimate even if no statistical methods were used.	Not Applicable	
Were any steps taken to minimize the effects of subjective bias when allocating animals/samples to treatment (e.g. **randomization procedure**)? If yes, have they been described?	Not Applicable	
Include a statement about **blinding** even if no blinding was done.	Not Applicable	
Describe **inclusion/exclusion criteria** if samples or animals were excluded from the analysis. Were the criteria pre-established?<br><br>If sample or data points were omitted from analysis, report if this was due to attrition or intentional exclusion and provide justification.	Yes	Materials and Methods + Figure legends when relevant
For every figure, are **statistical tests** justified as appropriate? Do the data meet the assumptions of the tests (e.g., normal distribution)? Describe any methods used to assess it. Is there an estimate of variation within each group of data? Is the variance similar between the groups that are being statistically compared?	Yes	Materials and Methods + Figure legends

Sample definition and in-laboratory replication	Information included in the manuscript?	In which section is the information available? (Reagents and Tools Table, Materials and Methods, Figures, Data Availability Section)
In the figure legends: state number of times the experiment was **replicated** in laboratory.	Yes	Materials and methods + Figure legends
In the figure legends: define whether data describe **technical or biological replicates**.	Yes	Figure legends

Ethics

Ethics	Information included in the manuscript?	In which section is the information available? (Reagents and Tools Table, Materials and Methods, Figures, Data Availability Section)
Studies involving **human participants**: State details of **authority granting ethics approval** (IRB or equivalent committee(s), provide reference number for approval.	Yes	Materials and Methods
Studies involving **human participants**: Include a statement confirming that **informed consent** was obtained from all subjects and that the experiments conformed to the principles set out in the WMA Declaration of Helsinki and the Department of Health and Human Services Belmont Report.	Yes	Materials and Methods
Studies involving **human participants:** For publication of **patient photos**, include a statement confirming that consent to publish was obtained.	Not Applicable	
Studies involving experimental **animals**: State details of **authority granting ethics approva**l (IRB or equivalent committee(s), provide reference number for approval. Include a statement of compliance with ethical regulations.	Not Applicable	
Studies involving **specimen and field samples:** State if relevant **permits** obtained, provide details of authority approving study; if none were required, explain why.	Not Applicable	

Dual Use Research of Concern (DURC)	Information included in the manuscript?	In which section is the information available? (Reagents and Tools Table, Materials and Methods, Figures, Data Availability Section)
Could your study fall under dual use research restrictions? Please check biosecurity documents and list of **select agents and toxins** (CDC): https://www.selectagents.gov/sat/list.htm	Not Applicable	
If you used a select agent, is the security level of the lab appropriate and reported in the manuscript?	Not Applicable	
If a study is subject to dual use research of concern regulations, is the name of the **authority granting approval and reference number** for the regulatory approval provided in the manuscript?	Not Applicable	

Reporting

The MDAR framework recommends adoption of discipline-specific guidelines, established and endorsed through community initiatives. Journals have their own policy about requiring specific guidelines and recommendations to complement MDAR.

Adherence to community standards	Information included in the manuscript?	In which section is the information available? (Reagents and Tools Table, Materials and Methods, Figures, Data Availability Section)
State if relevant guidelines or checklists (e.g., **ICMJE, MIBBI, ARRIVE, PRISMA**) have been followed or provided.	Not Applicable	
For **tumor marker prognostic studies,** we recommend that you follow the **REMARK** reporting guidelines (see link list at top right). See author guidelines, under 'Reporting Guidelines'. Please confirm you have followed these guidelines.	Not Applicable	
For **phase II and III randomized controlled trials**, please refer to the **CONSORT** flow diagram (see link list at top right) and submit the CONSORT checklist (see link list at top right) with your submission. See author guidelines, under 'Reporting Guidelines'. Please confirm you have submitted this list.	Not Applicable	

Data Availability

Data availability	Information included in the manuscript?	In which section is the information available? (Reagents and Tools Table, Materials and Methods, Figures, Data Availability Section)
Have **primary datasets** been deposited according to the journal's guidelines (see 'Data Deposition' section) and the respective accession numbers provided in the Data Availability Section?	Yes	Materials and methods
Were **human clinical and genomic datasets** deposited in a public access-controlled repository in accordance to ethical obligations to the patients and to the applicable consent agreement?	Not Applicable	
Are **computational models** that are central and integral to a study available without restrictions in a machine-readable form? Were the relevant accession numbers or links provided?	Not Applicable	
If publicly available data were reused, provide the respective **data citations in the reference list**.	Not Applicable	
